# EGFR/MET promotes hepatocellular carcinoma metastasis by stabilizing tumor cells and resisting to RTKs inhibitors in circulating tumor microemboli

**DOI:** 10.1038/s41419-022-04796-8

**Published:** 2022-04-15

**Authors:** Shouyang Song, Zhen Yu, Yajing You, Chenxi Liu, Xiaoyu Xie, Huanran Lv, Feng Xiao, Qiang Zhu, Chengyong Qin

**Affiliations:** 1 Shandong Provincial Hospital, Cheeloo College of Medicine, Shandong University, Jinan, 250021 Shandong P.R. China; 2 Shandong Provincial Hospital Affiliated to Shandong First Medical University, Jinan, 250021 Shandong P.R. China; 3 The First Affiliated Hospital of Xinjiang Medical University, Urumqi, 830054 Xinjiang P.R. China; 4 Shandong Provincial Engineering and Technological Research Center for Liver Diseases Prevention and Control, Jinan, 250021 Shandong P.R. China

**Keywords:** Liver cancer, Metastasis

## Abstract

The receptor tyrosine kinases (RTKs) family is well-recognized as vital targets for the treatment of hepatocarcinoma cancer (HCC) clinically, whereas the survival benefit of target therapy sorafenib is not satisfactory for liver cancer patients due to metastasis. EGFR and MET are two molecules of the RTK family that were related to the survival time of liver cancer patients and resistance to targeted therapy in clinical reports. However, the mechanism and clinical therapeutic value of EGFR/MET in HCC metastasis are still not completely clarified. The study confirmed that EGFR/MET was highly expressed in HCC cells and tissues and the phosphorylation was stable after metastasis. The expression of EGFR/MET was up-regulated in circulating tumor microemboli (CTM) to accelerate IL-8 production and resistance to the lethal effect of leukocytes. Meanwhile, highly expressed EGFR/MET effectively regulated the Ras/MAPK pathway and stabilized suspended HCC cells by facilitating proliferation and inhibiting apoptosis. Moreover, EGFR/MET promoted phosphorylation of hetero-RTKs, which was dependent on high-energy phosphoric acid compounds rather than their direct interactions. In conclusion, highly expressed EGFR/MET could be used in CTM identification and suitable for preventing metastasis of HCC in clinical practice.

## Introduction

Liver cancer is one of the most lethal cancers, causing the 6th carcinoma incidence and 4th cancer mortality in the world [1]. Hepatocellular carcinoma (HCC) is the most common type of liver cancer in clinical with high relapse and mortality rates [2]. Respectively, as the first-line drug for the treatment of liver cancer targeted to PDGFR/VEGFR family, sorafenib extends the survival time of patients by 2.8 or 2.3 months in two clinical trials among Caucasians and Asians, providing modest survival benefit [3, 4]. Recurrence and metastasis are responsible for the death of HCC patients [5]. Research has confirmed that the lung was the most common site of extrahepatic metastasis and significantly reduces the survival time of patients [6]. However, the mechanism in extrahepatic metastases of HCC remains unclear. According to several studies, tumor cells in the circulating system are precursors of metastasis and may contribute to tumor progression or metastasis [7, 8]. Also, anoikis resistance is essential for tumor metastasis [9, 10]. Multiply molecules and pathways including the Ras/MAPK pathway, contribute to stabilizing tumor cells in suspension [11]. However, mechanisms of survival and hematogenous metastasis of suspended HCC cells need to be completely explored.

Receptor tyrosine kinases (RTKs) are currently considered as critical roles in malignant transformation and cancer metastasis [12]. The human genome encodes for 58 RTKs, which can be divided into 20 subfamilies, and certain RTKs like VEGFR/PDGFR families, are used for clinical target therapy of primary HCC [13]. They belong to the same membrane protein family which can catalyze transferring of the γ phosphate of ATP to hydroxyl groups of tyrosinases on target proteins and play important roles in the control of most fundamental cellular processes including cell cycle, migration, metabolism, proliferation, differentiation and survival [14]. We have confirmed activation of VEGFR-1, one of the RTKs which leads to tumor angiogenesis and induces MMP-9-dependent invasion in HCC [15]. Some RTKs namely EGFR/MET can participate in metastasis of tumors by forming a molecular complex with integrin, mutation, amplification, and drug resistance [16–18]. RTKs may be vital for tumor cell survival in the circulating system and distal metastasis. For example, over-phosphorylation of EGFR in tumor cells cultured in suspension was observed [7, 10]. EGFR and Her2 are also identified as a biomarker on the surface of circulating tumor cells [19]. Additionally, MET-amplification has been discovered in the CTC of NSCLC patients [20]. However, the function of RTKs expressed in CTM is not completely clear. Expression and phosphorylation of RTKs are quite different in various tumors [21]. It is therefore that targeting the most expressional and functional RTKs is the key to the treatment of HCC.

In the present study, we determined the expression of RTKs proteins in CTM and cells and verified the important roles of EGFR/MET and the possibility of being used as a clinical treatment target. Furthermore, we identified the mechanism that EGFR/MET promotes metastasis and stabilizes HCC cell lines in hematogenous metastasis. These findings provide mechanistic evidence for us to design HCC medications targeting EGFR/MET, thereby benefiting patients with HCC in reducing the incidence of metastasis and prolonging survival time.

## Results

### EGFR/MET is highly expressed in human HCC tissues and cell lines compared to other RTKs families

**Fig. 1:**
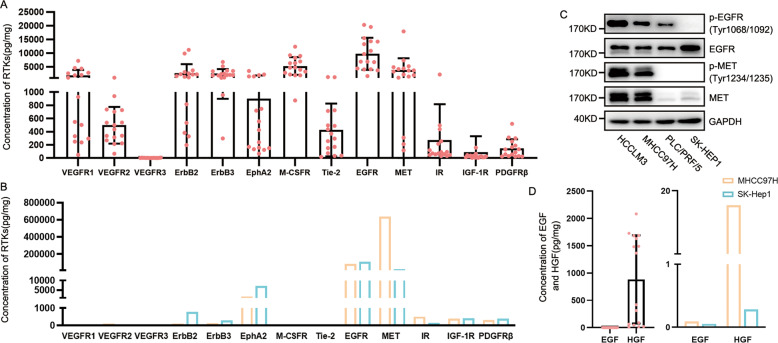
EGFR/MET were highly expressed in HCC tissue and HCC cell lines compared to other RTKs. **A**, **B** Luminex and ELISA assay were used for detection of expression of RTKs in HCC tissues and HCC cell lines. Data of tissues are shown as the mean ± SD. **C** Expression and phosphorylation of EGFR/MET in several HCC cell lines were determined by Western blot. **D** Expression of EGF and HGF in carcinoma tissue and HCC cells. Tissues and cells were lysis and Luminex was used for detection of concentration. Data of tissues are shown as the mean ± SD. Patient information for detecting concentration of RTKs and EGF/HGF in tissues is suppled in Table 4.

We firstly explored the expressions of RTKs in HCC tissues and HCC cell lines. Interestingly, the expressions of RTKs in HCC tissues and HCC cell lines varied greatly. Certain RTKs namely M-CSFR were highly expressed in HCC tissues whereas poorly expressed in cell lines. However, other RTKs namely the HER family were highly expressed in both HCC tissues and cell lines. Compared to the other RTKs we detected, the concentration of EGFR/MET was high expressional RTKs both in HCC tissues and HCC cell lines. Especially in HCC cells, expressions of EGFR and MET in MHCC97H were 54,548.83 and 607,470.8 pg/mg, respectively, and those of SK-HEP1 were 103,099.9 and 38,652.84 pg/mg, respectively, higher in expression than other RTKs (Fig. 1A, B). Additionally, both EGFR/MET in MHCC97H/LM3 cell lines with MET amplification were highly phosphorylated. In contrast, low phosphorylation of EGFR/MET was detected in PLC/PRF/5 and SK-HEP1 (Fig. 1C). Interestingly, the high expression level of HGF and low expression of EGF were observed in HCC tissue, though low concentration of EGF and HGF were expressed in HCC cells of tissues (Fig. 1D, Supplementary Fig. 1A). Low concentrations of EGF and HGF were observed in HCC cell lines, while HGF expression in MHCC97H was higher than SK-Hep 1 (Fig. 1D, Supplementary Fig. 1B). In brief, the EGFR/MET of the RTKs family was significantly expressed in HCC tissues and cells, and phosphorylation of EGFR/MET was related to the expression of MET.

### Expression of EGFR/MET is up-regulated in CTM but down-regulated in lung metastasis

**Fig. 2:**
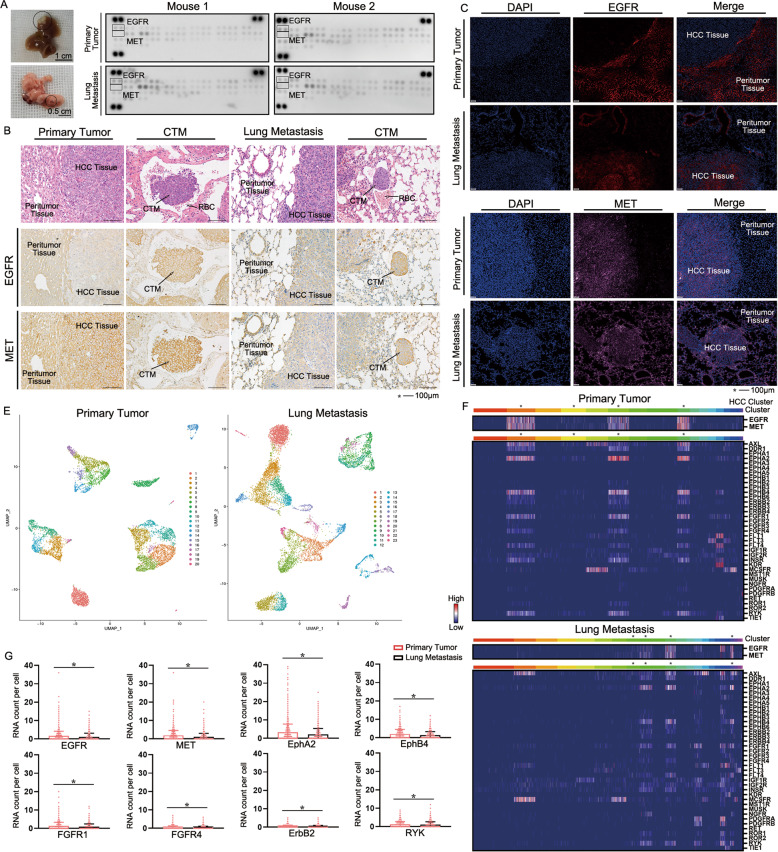
Expression of EGFR/MET is higher in CTM but lower in lung metastasis. **A** Gross image of HCC and lung metastasis in mice 60 days after injection of Hepa1-6 cell line. **B** Mice Phosphorylation Array was used for detection of RTK phosphorylation of HCC tissue separated from liver and lung. **C** Immunofluorescence analyzed the differences of EGFR/MET expression in cancer tissues and peritumor tissues in liver and lung. **D** Immunohistochemistry analyzed the differences of EGFR/MET expression in cancer tissues and CTM. **E** UMAP showed different clusters in carcinoma tissues of liver and lung. **F** Distribution of RTKs were showed in each cell of primary tumor and lung metastasis with Single cell RNA sequence by heatmap. **G** Difference of RTKs with high expression in carcinoma tissue between primary tumor and lung metastasis were showed. Data are shown as the mean ± SD, *P < 0.05.

Next, we verified whether the expressions of EGFR/MET using a xenograft model for lung metastasis of mice were stable during metastasis. In this model, Hepa1-6 cells were successfully metastasized from the left lobe of the liver to the lungs (Fig. 2A). Compared with the primary tumor, there was no significant difference in expression and phosphorylation levels of EGFR/MET in metastatic tumors (Fig. 2B). The expression of EGFR in HCC was markedly lower than that in peritumor tissues, while the expression of MET was substantially higher. However, the expression in lung metastases was just reversed compared with normal lung tissues (Fig. 2C). Interestingly, we found the presence of CTM in mice with widespread lung metastasis, and the expression level of EGFR/MET was higher than that in primary tumor and lung metastasis (Fig. 2D). In scRNA-seq, cells were divided into clusters for analysis (Fig. 2E). We found EGFR/MET were mainly expressed in HCC cells of carcinoma tissue, which differed from some RTKs for clinical targets expressed mainly in peritumor tissue such as the PDGFR family (Fig. 2F). However, expression of EGFR/MET in lung metastasis was lower than that in primary tumor, which was in agreement with most of the highly expressed RTKs (Fig. 2G). The ratios of RTKs expression in carcinoma tissue compared to whole tissues in primary tumor and lung metastasis were provided in Table 1. Collectively, expression of EGFR/MET increased during metastasis and decreased after metastasis, but phosphorylation of EGFR/MET kept stable after metastasis. It suggested that EGFR/MET was an important marker for the detection of CTM. As stable phosphorylation of EGFR/MET is suitable for the treatment of lung metastasis, targeting EGFR/MET in CTM may be more effective in the treatment of HCC.

**Table 1:** Expression of RTKs in carcinoma tissue.

	Liver			Lung			
GENE	Expressed in tumor	Expressed totally	Ratio	Expressed in tumor	Expressed totally	Ratio	P Value
****EGFR	**4536**	**4722**	**0.96061**	**1780**	**2703**	**0.658528**	**<0.0001**
****MET	**5143**	**5396**	**0.953113**	**1766**	**2174**	**0.812328**	**<0.0001**
AXL	3536	5604	0.630978	1991	8497	0.234318	
*DDR1	**2276**	**2641**	**0.861795**	**1259**	**1997**	**0.630446**	**0.0317**
EPHA1	16	29	0.551724	3	28	0.107143	
****EPHA2	**9029**	**9507**	**0.949721**	**3394**	**4141**	**0.819609**	**<0.0001**
EPHA3	0	14	0	3	114	0.026316	
EPHA4	10	41	0.243902	30	285	0.105263	
EPHA5	2	6	0.333333	5	8	0.625	
EPHB1	0	6	0	0	37	0	
EPHB2	148	159	0.930818	53	318	0.166667	
EPHB3	66	75	0.88	113	262	0.431298	
****EPHB4	**5616**	**6007**	**0.934909**	**2236**	**2613**	**0.855721**	**<0.0001**
EPHB6	847	914	0.926696	378	485	0.779381	
**ERBB2	**1031**	**1085**	**0.95023**	**489**	**632**	**0.773734**	**0.0011**
ERBB3	174	271	0.642066	192	306	0.627451	
ERBB4	0	6	0	0	3	0	
****FGFR1	**3603**	**4064**	**0.886565**	**1512**	**2961**	**0.510638**	**<0.0001**
FGFR2	165	199	0.829146	79	265	0.298113	
FGFR3	135	230	0.586957	96	280	0.342857	
****FGFR4	**1257**	**1326**	**0.947964**	**555**	**606**	**0.915842**	**<0.0001**
FLT1	119	1597	0.074515	108	2701	0.039985	
FLT3	31	882	0.035147	26	890	0.029213	
FLT4	1415	2320	0.609914	916	1255	0.72988	
IGF1R	20	761	0.026281	45	2199	0.020464	
IGF2R	660	1477	0.446852	464	2272	0.204225	
INSR	2362	3133	0.75391	1022	2073	0.493005	
KDR	32	2086	0.01534	17	707	0.024045	
MCSFR	186	3752	0.049574	228	7354	0.031004	
MST1R	22	23	0.956522	65	161	0.403727	
MUSK	11	12	0.916667	28	39	0.717949	
NGFR	45	77	0.584416	431	499	0.863727	
PDGFRA	191	235	0.812766	119	2844	0.041842	
PDGFRB	5	200	0.025	23	1477	0.015572	
RET	0	28	0	2	73	0.027397	
ROR1	463	496	0.933468	360	755	0.476821	
ROR2	41	47	0.87234	110	263	0.418251	
***RYK	**3315**	**3880**	**0.854381**	**1766**	**2935**	**0.601704**	**0.0007**
TIE1	11	345	0.031884	15	435	0.034483	

RTKs Ratio > 85% expression in carcinoma cells of liver and copy > 1000 was used for comparing difference in carcinoma tissue before and after metastasis. Mann-Whitney test was used for analysis. * < 0.05, ** < 0.01, *** < 0.001, **** < 0.0001.

### Anoikis resistance is related to up-regulation in expression and phosphorylation of EGFR/MET

**Fig. 3:**
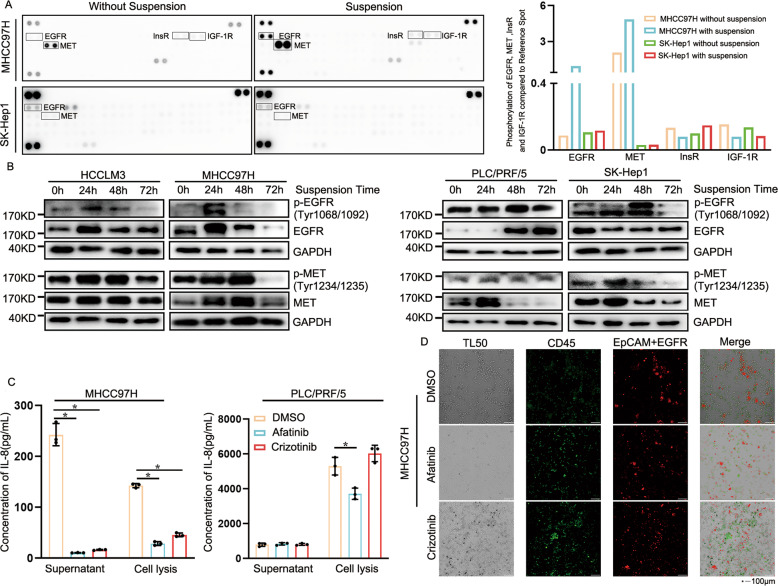
Expression and phosphorylation of EGFR/MET was up-regulated in suspended HCC cells. **A** RTK array was used to measure phosphorylation of RTKs in suspended HCC cell lines. **B** Western blot was used to analyze phosphorylation of EGFR/MET in HCC cell lines with 0, 24, 48, 72 h of suspension. Low concentration of suspended HCC cells was used in prevention of cluster formation. **C** ELISA was used to detect IL-8 secretion of MHCC97H and PLC/PRF/5 cell lines with EGFR/MET inhibitor. **D** Immunofluorescence analyzed the interactions between MHCC97H cells and leukocyte with or without EGFR/MET inhibitor. Data are shown as the mean ± SD, *n* = 3. *P < 0.05.

Since metastasis of CTM was closely related to anoikis resistance, we next observed the role of EGFR/MET in HCC cell lines of suspension. Phosphorylation RTKs array reported that phosphorylation of EGFR/MET in suspended HCC cell lines was enhanced and Western blot assays indicated that peak point appeared at 24–48 h (Fig. 3A, B). IL-8 synthesis/secretion could be interrupted by EGFR/MET inhibitor, which was observed in MHCC97H cell lines with high phosphorylation of EGFR/MET. However, in PLC/PRF/5 HCC cell lines with lower phosphorylation of EGFR/MET, the inhibitory effect of EGFR/MET inhibitors was greatly reduced, and secretion of IL-8 could interrupt by EGFR inhibitor was relatively obvious (Fig. 3C). Also, inhibition of EGFR/MET made HCCLM3 cell lines more easily killed by leukocytes and more leukocytes could survive (Fig. 3D), which were not beneficial to hematogenous metastasis of HCC cell lines. The findings suggested that EGFR/MET played an important role in the metastasis of HCC cells.

### EGFR/MET stabilizes suspended HCC cells and avoids the killing of leukocytes by regulating the Ras/MAPK pathway

**Fig. 4:**
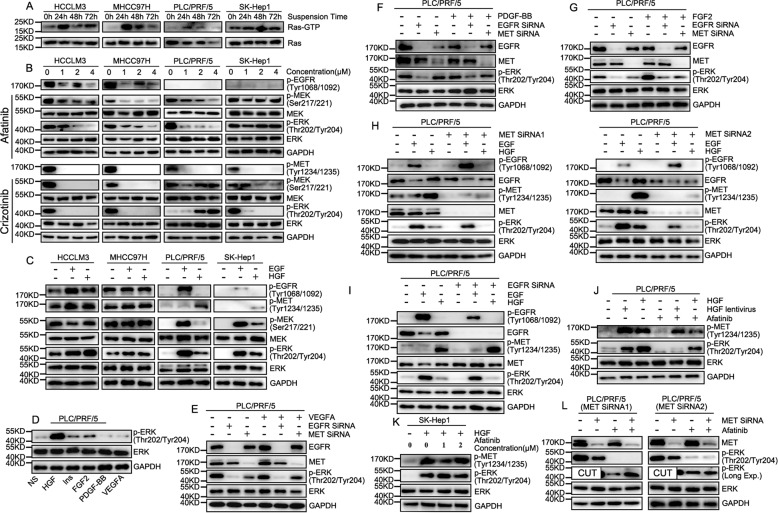
EGFR/MET were powerful in regulation of Ras/MAPK pathway based on high expression. **A** Ras Activation Assay Kit was used for detection of Ras-GTP in suspended HCC cell lines. Low concentration of suspended HCC cells was prevented from cluster formation and used for detection of Ras activation in 0, 24, 48 and 72 h. **B** Western Blot was used to confirm the regulation of EGFR/MET in Ras/MAPK pathway, including sensitivity of Ras/MAPK pathway to EGFR/MET inhibitors and **C** EGF/HGF in activation of Ras/MAPK pathway in different HCC cell lines. **D** 50 ng/ml of recombined ligands was used for RTKs phosphorylation and cells were harvested after 1 h for comparing activation of Ras/MAPK pathways. (**E**) VEGF, (**F**) PDGF-BB and (**G**) FGF2 were verified in restoration of Ras/MAPK pathway activation in PLC/PRF/5 cell line with EGFR/MET knock-down. **H** EGF/HGF was detected in activation of Ras/MAPK pathway of PLC/PRF/5 with MET knock-down and **I** EGFR knock-down. **J** Western Blot showed the effect of EGFR on MET down-regulation in EGFR inhibitor in PLC/PRF/5 cell line with exogenous/endogenous HGF stimulation. **K** EGFR inhibitor was used in detection of MET inhibition in SK-Hep1 cell line, which is not sensitive to EGFR inhibitor. **L** Effect of EGFR inhibitor was verified on PLC/PRF/5 cell line with MET knock-down.

As the Ras/MAPK pathway regulated by RTKs played an important role in tumor metastasis, the function of EGFR/MET in regulating the Ras/MAPK pathway will be further investigated in our subsequent research. Firstly, like phosphorylation of EGFR/MET, we found Ras-GTP was up-regulated in HCC cell lines in suspension (Fig. 4A). Both EGF and HGF could activate the corresponded receptor and lead to the activation of the Ras/MAPK pathway in HCC cell lines with low phosphorylation of EGFR/MET and activation of EGFR were was substantially promoted in the regulation of the Ras/MAPK pathway. However, HCC cell lines with high phosphorylation of EGFR/MET were not sensitive to EGF and HGF. In contrast, the inhibitor of EGFR/MET could down-regulate the Ras/MAPK pathway in HCC cell lines with high phosphorylation of EGFR/MET instead of a condition of low phosphorylation of EGFR/MET. In brief, the Ras/MAPK pathway could be regulated by the basal phosphorylation levels of EGFR/MET (Fig. 4B, C). While fully activating the corresponding RTKs by a sufficient amount of ligand, we found that MET worked more effectively inactivating the Ras/MAPK pathway than other RTKs in treating clinical HCC (Fig. 4D). However, simple knockdown of EGFR/MET, EGFR in particular, could directly inhibit the Ras/MAPK pathway, which could barely be restored by activating other RTKs (FGFR, VEGFR, and PDGFR) based on their low expression. Moreover, the Ras/MAPK pathway could not respond to activation of RTKs (PDGFR and VEGFR) poorly expressed (Fig. 4E–G). However, based on high expression and activation of EGFR or MET could offset the effect of knockdown in each other on the Ras/MAPK pathway to some degree (Fig. 4H, I). EGFR and MET synergistically regulated the Ras/MAPK pathway. EGFR inhibitors could decrease the phosphorylation level of MET that was activated endogenously or exogenously in HCC cell lines which were sensitive to EGFR inhibitors, but it did not work effectively in HCC cell lines with low phosphorylation of EGFR (Fig. 4J, K). Moreover, MET knockdown could weaken the effect of EGFR inhibitors on the interruption of the Ras/MAPK pathway (Fig. 4L). Different expressions of EGF/HGF indicated that activation of EGFR/MET relied on different ways in vivo and higher levels of HGF in HCC tissue allowed MET to play an important role in the regulation of the Ras/MAPK pathway.

**Fig. 5:**
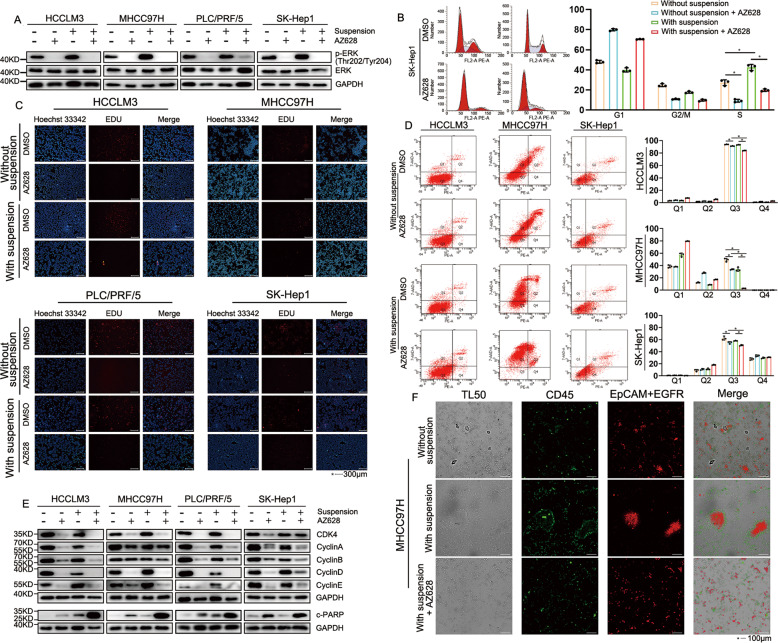
Ras/MAPK pathway stabilized suspended HCC cell lines and avoid killing of leukocytes. Cells were divided in 4 groups: (a) cultured without suspension (b) cultured with suspension (c) cultured without suspension, 10 µM of AZ628 were used for Ras/MAPK pathway inhibition(d) cultured with suspension, 10 µM of AZ628 were used for Ras/MAPK pathway inhibition. Cells in different group were detected in (**A**) phosphorylation of ERK by Western blot, (**B**) cell cycle by flow cytometry, (**C**) DNA synthesis by EDU, (**D**) apoptosis by flow cytometry and (**E**) Cyclin, CDK4 and PARP by Western blot. **F** Immunofluorescence was used in determining interactions between MHCC97H and leukocyte with or without Ras/MAPK inhibitor. Data of flow cytometry are shown as the mean ± SD, *n* = 3. *P < 0.05.

Next, we analyzed the activation of ERK in suspended HCC cell lines. The findings were identical to Ras-GTP indicating that the phosphorylation level of ERK was up-regulated in suspended HCC cell lines (Fig. 5A). Following the inhibition of the Ras/MAPK pathway, DNA synthesis and G2/S-phase of the cell cycle were decreased and the G1-phase of the cell cycle increased in HCC with or without suspension. Moreover, S-phase increased in suspended HCC cells, indicating that the Ras/MAPK pathway maintained the growth of HCC cell lines (Fig. 5B, C). Flow cytometry confirmed that inhibition of the Ras/MAPK pathway in HCC cells with or without suspension could promote the increase of apoptosis in HCC cell lines, but the effect in HCC cells with suspension was significant (Fig. 5D). We also found all cyclin proteins were down-regulated followed by Ras/MAPK inhibition. In detail, Cyclin A/B were less interfered with by Ras/MAPK inhibitors and poorly expressed in suspended HCC cell lines. Conversely, Cyclin E was highly expressed in suspended HCC cell lines and could be easily down-regulated by Ras/MAPK inhibitors. Both cultured in suspension and Ras/MAPK inhibitors could activate PARP, a protein major in DNA repairing in HCC cell lines [22]. Moreover, a combination of Ras/MAPK inhibitor and suspension caused higher activation of PARP in HCC cell lines, which was related to higher apoptosis of HCC cell lines (Fig. 5E). Also, inhibition of the Ras/MAPK pathway made suspended HCC cells more easily to be killed by leukocytes (Fig. 5F). Collectively, the data indicated that the Ras/MAPK pathway induced by EGFR/MET stabilized suspended HCC cell lines and prevented the lethal effect of leukocytes. Moreover, it was more difficult for suspended HCC cells to survive under a condition of poorly phosphorylated ERK.

### EGFR/MET participates in the phosphorylation of multiple RTKs family members

**Fig. 6:**
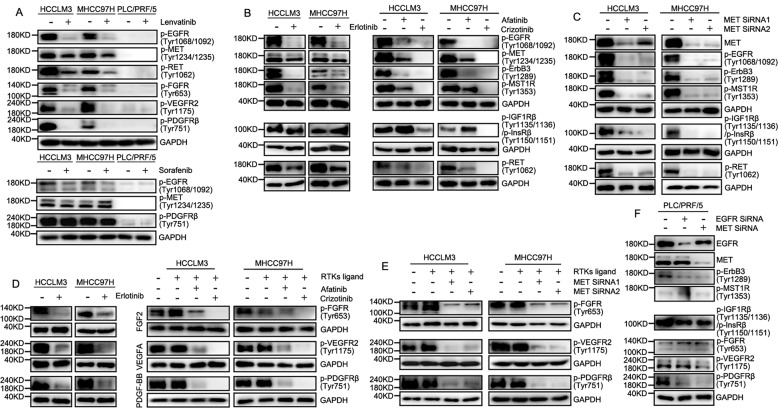
EGFR/MET participated in regulation of phosphorylation of hetero-RTKs. **A** Western Blot was used to observe phosphorylation of RTKs in HCCLM3, MHCC97H and PLC/PRF/5 cell lines with Lenvatinib or Sorafenib added. **B** Western blot was used to determine the effect of down-regulation of EGFR/MET on hetero-RTKs, including: Phosphorylation of EGFR, MET, Her3, RON, IR/IGF-1R and RET in HCC cell lines with EGFR/MET inhibitor added. **C** Phosphorylation of EGFR, Her3, RON, IR/IGF-1R and RET in HCC cell lines with MET knock-down. **D** Phosphorylation of FGFR1, VEGFR2 and PDGFRβ with ligand stimulated in HCC cell lines with EGFR/MET inhibitor added. **E** Phosphorylation of FGFR1, VEGFR2 and PDGFRβ with ligand stimulated in HCC cell lines with MET knock-down. **F** Phosphorylation of RTKs in PLC/PRF/5 cell line with EGFR or MET knock-down.

Based on the high expression of EGFR/MET in HCC tissue/cell lines and stable expression in metastasis, the regulatory effects of EGFR/MET on the phosphorylation level of other RTKs were investigated. In this study, Lenvatinib, as an inhibitor of PDGFRβ/VEGFR/FGFR/RET, was effective in suppressing EGFR/MET and targeting RTKs in MHCC97H and HCCLM3, but less effective on PLC/PRF/5. In contrast, Sorafenib, as an inhibitor of PDGFRβ/VEGFR/Raf, had less effect on EGFR/MET, and barely suppressed the function of PDGFRβ in MHCC97H and LM3 (Fig. 6A). Also, we found that high phosphorylation of EGFR/MET could maintain the hyperphosphorylation status of some RTKs which were clinical targets for HCC treatment, such as PDGFRβ and FGFR1, and it was obvious in HCC cell lines with MET amplification. Briefly, both EGFR/MET inhibitors could reduce phosphorylation of ErbB family, MET family, and RET, and MET inhibitors could additionally interfere with the InsR family (Fig. 6B). MET knockdown exhibited the same effect as MET inhibitors in the suppression of phosphorylation of multiple RTKs (Fig. 6C). Additionally, EGFR/MET inhibitors could effectively interrupt phosphorylation of FGFR, VEGFR, and PDGFRβ which were for clinical therapy, even stimulated by RTK ligand (Fig. 6D). Similarly, MET knock-down also inhibited phosphorylation of those RTKs (Fig. 6E). EGFR/MET had an impaired ability to influence the phosphorylation of hetero-RTKs in HCC cell lines with low phosphorylation (Fig. 6F). In brief, EGFR/MET also affected the inhibitory effect of clinical anti-HCC drugs on RTKs, which might lead to treatment failure and extrahepatic metastasis.

### Phosphorylation of EGFR/MET is related to high-energy phosphate compounds, not EGFR/MET hetero-dimer

**Fig. 7:**
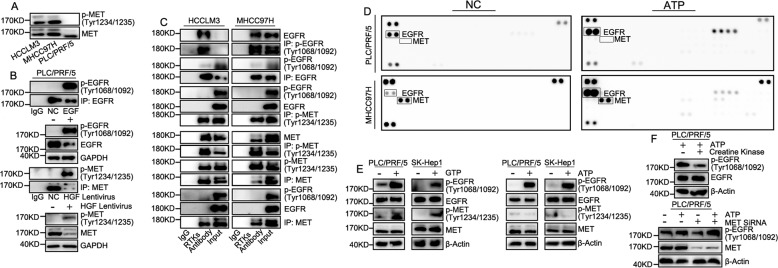
Phosphorylation of EGFR/MET was related to high-energy phosphate compounds but not EGFR/MET hetero-dimer. **A** MET phosphorylation ratio was compared in different HCC cell. Co-immunoprecipitation was used for detection of crosstalk in (**B**) EGFR/p-EGFR or MET/p-MET after stimulation of ligand and (**C**) EGFR-EGFR, MET-MET and EGFR-MET in cells with MET amplification. **D** RTKs Phosphorylation Array was used for detection of phosphorylation of RTKs after incubation with 0.2 mM ATP in PLC/PRF/5 and MHCC97H cell lines. **E** Western Blot was used for detection of phosphorylation of EGFR/MET stimulated by ATP/GTP. 0.2 mM of ATP/GTP was incubated with cell lysate of PLC/PRF/5 with kinase buffer for 1 h at 37 °C. **F** Phosphorylation of EGFR in PLC/PRF/5 cell lysis with 0.2 mM of ATP with creatine kinase or MET knock-down was detected by Western Blot.

**Fig. 8:**
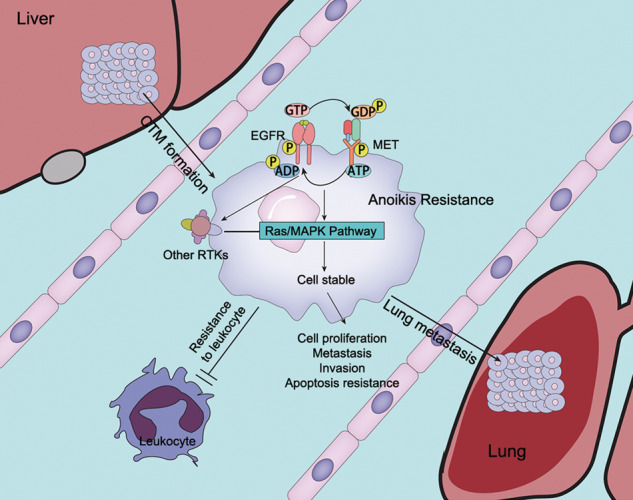
A proposed model of EGFR/MET-mediated in HCC and metastasis. After metastasis to blood, both expression and phosphorylation of EGFR/MET was up-regulated in CTM. Based on high expression, EGFR/MET could induce phosphorylation of other RTKs via ATP production, and over-phosphorylation of RTKs were independent to RTKs ligand and resistant to RTKs inhibitors. Compared to other RTKs, EGFR/MET could more effectively activate Ras/MAPK pathway. Activation of Ras/MAPK pathway participate in cell proliferation, resistance to apoptosis and stabilization of suspended HCC cells. Also, EGFR/MET mediated in helping CTM to avoid killing effect of leukocytes.

Next, we focused on possible mechanisms related to the phosphorylation of EGFR/MET. Interestingly, we found that phosphorylation of MET was affected by MET amplification (Fig. 7A). Phosphorylated EGFR/MET, either endogenous or induced by ligands, tended to form homo-dimers and it was almost impossible for EGFR/MET to form hetero-dimer, whether EGFR/MET was phosphorylated or not (Fig. 7B, C). It suggested that phosphorylation of EGFR/MET was not affected by hetero-dimer. In further research, we found sufficient ATP in the solution could effectively promote phosphorylation of most RTKs, even in HCC cell lines with MET-amplification, but MET was not affected (Fig. 7D). MET phosphorylation was affected by GTP rather than ATP (Fig. 7E). Phosphorylation of EGFR induced by ATP was not affected by MET, but the factor creatine kinase that degraded ATP could reduce the level of EGFR phosphorylation (Fig. 7F). It suggested that EGFR could be directly phosphorylated by high-energy phosphate compounds in the absence of ligands, but MET relied on GTP, MET amplification, or ligand induction (Fig. 8).

## Discussion

RTKs nowadays are considered to be critical in tumorigenesis, intrahepatic metastasis, and distal metastasis of liver cancer [23]. The correlation exists between multiply RTKs and recurrence times of liver cancer [13]. Currently, the clinical targeted drugs for liver cancer are mainly aimed at RTKs. For example, sorafenib, as a multi-RTKs inhibitor that exerts an antiangiogenic effect by VEGFR 2/3 and PDGFR-β, is typically used as the first-line therapy in patients with primary advanced HCC [24, 25]. However, sorafenib resistance can cause therapeutic failure and metastasis of HCC [26]. Research has shown that RTKs EGFR/MET are responsible for sorafenib resistance [27, 28] and they were highly phosphorylated in multiply carcinoma cells including the lungs, breasts, and pancreas [21]. Interestingly, highly expressed RTKs VEGFR1 and PDGFRβ in HCC tissues were barely expressed in HCC cell lines in the present study. Instead, RTKs EGFR/MET, which was targeted for HCC treatment, were highly expressed in both HCC tissues and HCC cell lines. Especially in HCC cell lines, the EGFR/MET levels were higher than other common RTKs. It suggested that some RTKs inhibitors sorafenib, tended to be effective to primary HCC mass by interruption of neoplastic angiogenesis, but low expression of VEGFR/PDGFRβ or compensation effect from EGFR/MET in HCC cell lines were invalid in metastasis. Thus, discovering the mechanism of EGFR/MET promoting distal metastasis may be helpful for the treatment and recurrence of HCC.

Extrahepatic metastasis is easily observed in patients with HCC [29]. Because of the high level of expression, EGFR/MET has been reported as a hopeful target for HCC and combination therapy may benefit patients with prolonged survival time by interaction with multiply molecules like PARP [22]. Lung metastasis is most frequently observed in extrahepatic metastasis [6]. In the C57 xenograft model, CTM with high EGFR/MET expression was detected in the blood vessels of lung metastasis mice, which was higher than that in tumor tissues, indicating EGFR/MET as a suitable marker for CTC/CTM detection. Meanwhile, stable phosphorylation of EGFR/MET makes targeted therapy possible for lung metastases. However, expression of EGFR/MET in CTM is higher than that in lung metastasis, suggesting that it is necessary to target CTM. EGFR/MET may participate in the metastasis of carcinoma through circulating tumor cells. For example, EGFR has been identified as a biomarker on the surface of circulating tumor cells [19]. Over phosphorylation of EGFR has been observed in suspended carcinoma cells such as lung and gastric cancer cells [7, 10]. Also, a clinical trial has shown that MET inhibition is related to reducing CTC [30]. Cancer cells with anoikis resistance are suitable for entering the circulating or lymphatic system, which is essential for successful cancer metastasis [31], and this may be one mechanism of tumor metastasis promotion. Interference function of EGFR/MET attenuated anoikis resistance of tumor cells [32, 33]. Similarly, our data showed the phosphorylation of EGFR/MET phosphorylation was keeping stable or up-regulated in HCC cell lines cultured at beginning of suspension in this study. As the suspension time passed by, phosphorylated EGFR/MET decreased. Most tumor cells in the circulating system survive for only a few minutes and living cells are prone to combine with other tumor cells and non-tumor cell types in peripheral blood, which are more aggressive than single cells in cancer metastasis [34, 35]. EGFR/MET was also related to cytokines synthesis/secretion and resistance to leukocytes of HCC cell lines and inhibition of EGFR/MET could interrupt metastasis of HCC cell lines, especially in CTM with higher expression of EGFR/MET.

The Ras/MAPK pathway is considered a survival factor for carcinoma cells, and RTKs produce a similar mechanism in regulating the Ras/MAPK pathway [14, 36]. Increased expression and activity of the Ras/MAPK pathway have been observed in mouse HCC models and human HCC tissues and they have been reported as important factors for anoikis resistance and metastasis [36, 37]. Over-activated Ras/MAPK pathway is considered to be associated with tumorigenesis and metastasis [38]. We found that the ability of RTKs to regulate the Ras/MAPK pathway was correlated with phosphorylation and expression levels in cells. Moreover, the RTKs family has cross-regulation on the Ras/MAPK pathway and RTKs inhibitors can sometimes cause Ras/MAPK re-activation, namely IR/IGF-1R knockdown leading to up-regulation of EGFR [39]. On the other hand, for RTKs with extremely low expression levels, even sufficient ligands cannot trigger the regulation of the Ras/MAPK pathway. Thus, EGFR and MET play important roles in the regulation of the Ras/MAPK pathway based on their high expression in the RTKs family. Same as the result of phosphorylation of EGFR/MET, we found that ERK was highly phosphorylated in suspended HCC cell lines. Inhibition of Ras/MAPK pathway promoted apoptosis, declination of DNA synthesis, and interfered cell cycle, which was observed in HCC cell lines both cultured without and with suspension. Also, over-activation of ERKs in suspended HCC cell lines could offset but not completely reverse the damage from the suspension environment, thereby maintaining the stability of suspended cells and preventing lethal effect of leukocytes in degree with mild activation of PARP. Based on the previously described results, EGFR/MET stabilized HCC cell lines and promoted distal metastasis by activating the Ras/MAPK pathway.

RTKs have a similar effect on the activation of downstream signaling pathway Ras/MAPK pathway [14]. Our previous experiments have confirmed the advantage in the concentration of EGFR/MET in highly aggressive HCC cell lines and MET amplification is one of the important reasons for the hyper-phosphorylation of EGFR. It has been reported that EGFR/MET may be involved in resistance of RTKs target therapy [40, 41], and MET amplification is important in tumor metastasis [42]. In our result, EGFR/MET could indirectly regulate the phosphorylation of multiple RTKs and the effect was superior to their ligands in cells with MET amplification. Thus, highly phosphorylated EGFR or MET could easily induce resistance to RTKs inhibitors. Interestingly, EGFR/MET didn’t directly induce phosphorylation of hetero-RTKs via the action of kinases. High-energy phosphate compounds represented by ATP played important roles in RTKs phosphorylation [13]. Also, EGFR/MET over-phosphorylation might cause regulation disorder of high-energy phosphate compounds, thereby promoting hetero-RTKs phosphorylation. EGFR could be easily phosphorylated by ATP/GTP in the absence of EGF, that is, EGFR might be more dependent on high-energy phosphate compounds in the cell than ligands in phosphorylation. Moreover, MET was not sensitive to ATP, and HGF not EGF was over-expressed in cell lines with MET amplification and HCC tissues, suggesting that MET was easier to perform biological functions than EGFR in terms of ligand-dependent phosphorylation. Thus, dysfunctional RTKs could definitely affect the functions of the Ras/MAPK pathway.

In conclusion, we have demonstrated that EGFR/MET are as the two of the most expressed in the RTKs family in both HCC tissue and cells. The study also clarifies the relationship between EGFR/MET and CTM. Activation of EGFR/MET caused by complicated factors helps suspended HCC cell lines to maintain cell stability and distal metastasis. Targeting EGFR/MET may provide opportunities for the prevention of metastasis of HCC.

## Materials and methods

### Cell preparation

Human HCC cell lines MHCC-97H (Zhong Qiao Xin Zhou Biotechnology Co., Ltd, China), PLC/PRF/5 (Hongbo Biotechnology, China), HCCLM3, SK-Hep1, and mouse HCC cell line (Procell, China) were cultured in DMEM containing high glucose (Biological Industries, Israel) with 10% fetal bovine serum (Biological Industries, Israel) at 37 °C in a humidified atmosphere containing 5% CO_2_. 100 mg/mL penicillin G and 50 μg/mL streptomycin (Biological Industries, Israel) were added. All cell lines were identified by STR and mycoplasma tests and all were used within 3 months after thawing early passage cells.

### Suspension culture

Of 3% Poly-HEMA (Sigma, MO, USA) was dissolved in ethanol for preventing HCC cell lines adhesion. Tissue culture plates were coated with 1 ml of Poly-HEMA solution and dried overnight at room temperature in a laminar flow hood. All cells were sub-cultured, re-plated in suspension condition, and harvested at indicated time points for detection.

### EGFR/MET knockdown cell construction

For EGFR/MET knockdown, siRNAs were transfected into MHCC97H/HCCLM3/PLC-PRF-5 cells using Lipofectamine 3000 (Thermo Fisher, MD, USA). Cells were cultured for further experiments after 48 h. The sequences of siRNAs were listed in Table 2.

**Table 2:** Sequences of EGFR/MET SiRNAs.

Gene	Target sequence
EGFR	GCAGUCUUAUCUAACUAUGAUGCAA
MET SiRNA1	CUGGUUUUGUCGACGUAAA
MET SiRNA2	CGAGGGAAUCAUCAUGAAA

### RTKs and Ras/MAPK inhibitors and RTKs ligands

Sorafenib (VEGFR and PDGFRβ), Lenvatinib (RET, VEGFR, PDGFRβ, and FGFR), Erlotinib (EGFR), Afatinib (EGFR), Crizotinib (MET), and AZ628 (Ras/MAPK) were purchased from Selleck (TX, USA) and dissolved in DMSO. Recombined EGF, HGF, FGF2, PDGF-BB, and VEGF were purchased from PeproTech (NJ, USA), and recombined insulin was bought from Selleck (TX, USA).

### Western blot and co-immunoprecipitation (Co-IP)

The composition of Co-IP buffer was 50 mM Tris, 150 mM NaCl, 1% NP40, 0.5% sodium deoxycholate, 1 mM EDTA, 1 mM PMSF and 1× protease inhibitor cocktail (CWBIO, China). Co-IP and Western blot were performed as described [15] and all antibodies used were listed in Table 3. Markers were used for indicating protein lower than 180 KD (PageRuler 26616, Thermo Scientific, MA, USA) and more than 180 KD (PL00003, ProteinTech, China).

**Table 3:** Purchase source and catalog ID of antibodies used.

Antibody for Western blot		
Assay	Enterprise	Detail
EGFR	Abcam	ab52894
pEGFR	CST	3777T
MET	CST/Abcam	8198T/ab51067
pMET	CST	3077T
pHer3	Abcam	ab255607
pMST1R	Abcam	Ab124671
pIGF1R/pInsR	CST	3021T
pRET	Abcam	ab51103
pFGFR	Abcam	ab173305
pVEGFR2	CST	2478T
pPDGFRβ	Abcam	ab218534
MEK	Abcam	ab178876
p-MEK	CST	9154T
ERK	Abcam	ab184699
p-ERK	CST	4370T
Ras	CST	3339T
CDK4	CST	12790T
CyclinA	CST	4656T
CyclinB	CST	12231T
CyclinD	CST	2978T
CyclinE	CST	4129T
C-PARP	Abcam	ab32064
B-Actin	ProteinTech	Cat No. 66009-1-Ig
GAPDH	ProteinTech	Cat No. 60004-1-Ig
Antibody For Co-immunoprecipitation		
Assay	Enterprise	Detail
EGFR	santa	sc-373746
MET	CST	sc-8057
IgG	santa(M)/CST(R)	sc-2025/3900S
p-EGFR	CST	3777T
pMET	CST	3077T
Antibody For Immunofluorescence		
CD45	Abcam	52894
EpCAM	Abcam	187372
Antibody For Immunohistochemistry		
EGFR	santa	sc-373746
MET	santa	sc-514148

### Luminex, ELISA, Array kit, and Ras Activation Assay Kit

Luminex Kit (R&D system, CA, USA) was coated with RTKs (VEGFR1-3, ErbB2-3, Tie, EphA2, and MCSFR) and RTKs ligands (EGF and HGF). ELISAs (EGFR, MET, PDGFRβ, IGF-1R, and InsR) were used to detect the concentration of RTKs which Luminex Kit didn’t contain. Phosphorylation of RTKs was performed by Human/Mice Phospho-RTKs Array Kit (R&D system, CA, USA) according to the manufacturer’s instructions. Image pro plus 6.0 was used for intensity quantification. Ras Activation Assay Kit (Sigma, MO, USA) was used for the detection of Ras activation.

### Flow cytometry

All HCC cell lines were harvested and resuspended at a concentration of 10^5^/ml. Trypsin digestion was performed in cells with or without suspension at the same time to dissociate clusters into single cells and reduce interference from trypsin. PI/RNAse was used for detecting cell cycle and Annexin V/PI was used for detecting apoptosis of HCC cell lines. Flow cytometry was performed according to the manufacturer’s instructions.

### Immunohistochemistry/Immunofluorescence

Paraffin specimens from patients with HCC and carcinoma tissues of mice were performed according to the manufacturer’s instructions. Immunofluorescence assays were conducted in a manner blinded to the sample identity. Cells cultured with or without suspension were harvested and dried on coverslips with Poly-L-lysine (Beyotime, China) coated. EDU assay (KeyGEN, China) was used for detecting DNA synthesis of HCC cell lines.

### Co-culture of HCC and leukocytes

Leukocytes were collected from healthy donor blood by gradient centrifugation with leukocytes separation (Solarbio Technologies, China). After being cultured with DMSO or inhibitors for 24 h, HCC cells lines were re-suspended and the supernatant was discarded. Then, equal leukocytes were added to the culture medium with MHCC97H cell line and were co-cultured for 24 h in suspension. Cell mixtures were collected, dried on coverslips with Poly-L-lysine coated, and observed by immunofluorescence with EpCAM + EGFR and CD45 staining.

### Animal xenograft model

5-week-old C57 male mice (Charles River Ltd, Beijing, China) were employed to construct xenograft tumor models. The mice were anesthetized with 50 mg/kg Zoletil®50 (VIRBAC, France) combined with 5 mg/kg Xylazine hydrochloride (Selleck, TX, USA) by intraperitoneal injection, and 10^5^ Hepa1-6 cells were injected into the left lobe of the liver in each mouse. The animals were sacrificed after 60 days and the presence of any extrahepatic metastasis except lungs was considered to be a failure, even lung metastasis model was successfully established. Tumor tissues from the liver and lung in the same mouse were collected for phosphorylation detection, immunohistochemistry, immunofluorescence, and scRNA-seq.

### Single-cell RNA sequence (scRNA-seq) and analysis

ScRNA-seq was performed by Annoroad Gene Tech (Beijing, China). Carcinoma tissues were separated from the xenograft model in 48 h and a library was constructed as manufacturer’s instructions. CellRanger was applied to identify cell barcodes in reads1 and UMI marks in different transcripts, and the corresponding reads2 were compared referring to genome through STAR to determine source gene of the reads and complete quantification of gene expression. RBC in cells containing <200 expressed genes or mitochondria UMI rate >80% were excluded. Then, mitochondrial genes were removed in the expression table. After normalizing UMI data of gene expression in each cell by Seurat analysis and UMI data dimension reduction by PCA, the top 20 principal components were selected for cell classification and UMAP construction based on graph-based clustering method. FindAllMarkers function by the Wilcox rank-sum test algorithm (lnFC > 0.25; *p*-value < 0.05; min. pct > 0.1) was used for marker gene identification.

### Clinical HCC specimens

Paraffin specimens and HCC resections of HCC tissues were collected from the Department of Hepatobiliary Surgery, Shandong Provincial Hospital, Cheeloo College of Medicine, Shandong University. Paraffin specimens of HCC resection for IHC were obtained from patients with further HCC metastasis within ten years. RTKs concentration of HCC resections was determined by Luminex and Elisa. Details of the clinicopathologic characteristics of the recruited HCC patients were shown in Table 4.

**Table 4:** Clinicopathologic characteristics of HCC patients in cohorts for IHC and RTKs concentrations.

Characteristics	Cohort 1 (*n* = 23, for IHC) No. of patients (%)	Cohort 2 (*n* = 16, for Luminex detecting RTKs and EGF/HGF) No. of patients (%)
Gender		
Male	22 (95.7%)	14 (87.5%)
Female	1 (4.3%)	2 (12.5%)
Age		
<60	13 (56.5%)	10 (62.5%)
≥60	10 (43.5%)	6 (37.5%)
HBV infection		
Yes	12 (52.2%)	12 (75%)
No	11 (47.8%)	4 (25%)
Tumor size		
<5 cm	5 (21.7%)	10 (62.5%)
≥5 cm	18 (78.3%)	6 (37.5%)
Receiving anti-HBV treatment before resection		
Entecavir	0 (0%)	2 (12.5%)
Adefovir Dipivoxil	0 (0%)	1 (6.3%)
Lamivudine	1 (4.3%)	0 (0%)
Recall lacking	2 (8.6%)	0 (0%)
No	20 (87%)	13 (81.3%)
Receiving non-resection treatment before resection		
TACE	1 (4.3%)	2 (12.5%)
Microwave Ablation	1 (4.3%)	0 (0%)
No	21 (91.4%)	14 (87.5%)
Receiving any RTKs inhibitors treatment before resection		
Yes	0 (0%)	0 (0%)
No	23 (100%)	16 (100%)
HCC metastasis existence before resection		
Intrahepatic thrombus	0 (0%)	1 (6.3%)
Retroperitoneal	0 (0%)	1 (6.3%)
Common bile duct	1 (4.3%)	0 (0%)
No	22 (95.7%)	14 (87.5%)

### Statistical analysis

GraphPad Prism 8 software (GraphPad, CA, USA) was used for data analysis. The statistical significance of differences between different experimental cell groups was determined by *t*-tests. Except results of scRNA-seq failed in conforming to Gaussian distribution, all data were estimated of variation within each group of data, and *P*-value used in all analyses was two-tailed, and the value <0.05 was considered statistically significant. As for the results of scRNA-seq, a Mann-Whitney test was used for analyzing gene expression differences among samples.

## Supplementary information

Supplement

Supplement 1

Supplemental Table 1

Supplemental Table 2

Supplemental Table 3

Supplemental Table 4

Reproducibility Checklist

Author Contribution Form

Western Blot Origin

## Data Availability

The datasets used and/or analyzed during the current study are available from the corresponding author on reasonable request.
