# Supplementary material for: EGFR/MET promotes hepatocellular carcinoma metastasis by stabilizing tumor cells and resisting to RTKs inhibitors in circulating tumor microemboli
Source: Cell Death Dis. 2022 Apr 15;13(4):351. doi: 10.1038/s41419-022-04796-8 (PMC9012802; doi:10.1038/s41419-022-04796-8)
Supplement: Supplementary file 1 — Supplement [file 41419_2022_4796_MOESM1_ESM.docx]

**Supplementary Figure 1 Expression of EGF and HGF in HCC patients and HCC cell lines.**

(A) Immunohistochemistry was used for detection of EGF and HGF expressed in liver cancer tissue of patients. The characteristics of patients were list in table 1, named ‘Cohort 1’. (B) FACS was used for finding out expression of EGF and HGF in MHCC97H and SK-hep1 cell lines. Group Ctr was used for gate setting of SSC, FSC and Fluorescence intensity.

**Supplementary Table legends**

**Supplementary Table 1 Clinicopathologic characteristics of HCC patients in cohorts for IHC and RTKs concentrations.**

**Supplementary Table 2 Expression of RTKs in carcinoma tissue.**

RTKs Ratio > 85% expression in carcinoma cells of liver and copy > 1000 was used for comparing difference in carcinoma tissue before and after metastasis. Mann-Whitney test was used for analysis.

**Supplementary Table 3 Purchase source and catalog ID of antibodies used.**

**Supplementary Table 4 Sequences of EGFR/MET SiRNAs.**
