# Supplementary figures and images for: EGFR/MET promotes hepatocellular carcinoma metastasis by stabilizing tumor cells and resisting to RTKs inhibitors in circulating tumor microemboli
Source: Cell Death Dis. 2022 Apr 15;13(4):351. doi: 10.1038/s41419-022-04796-8 (PMC9012802; doi:10.1038/s41419-022-04796-8)

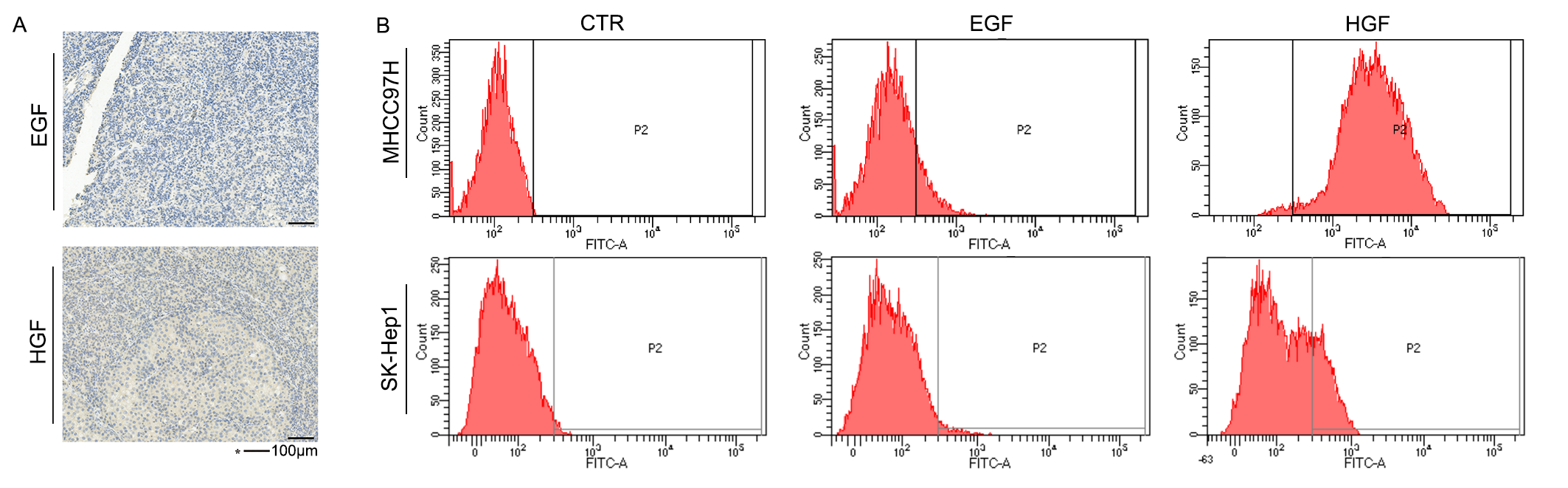

Supplement: Supplementary file 2 — Supplement 1 [file 41419_2022_4796_MOESM2_ESM.tif]
