# Supplementary material for: EGFR/MET promotes hepatocellular carcinoma metastasis by stabilizing tumor cells and resisting to RTKs inhibitors in circulating tumor microemboli
Source: Cell Death Dis. 2022 Apr 15;13(4):351. doi: 10.1038/s41419-022-04796-8 (PMC9012802; doi:10.1038/s41419-022-04796-8)
Supplement: Supplementary file 3 — Supplemental Table 1 [file 41419_2022_4796_MOESM3_ESM.docx]

| Characteristics | Cohort 1 (n = 23, for IHC) No. of patients (%) | Cohort 2 (n = 16, for Luminex detecting RTKs and EGF/HGF) No. of patients (%) |
| --- | --- | --- |
| Gender  Male  Female | 22(95.7%)  1(4.3%) | 14(87.5%)  2(12.5%) |
| Age  <60  ≥60 | 13(56.5%)  10(43.5%) | 10(62.5%)  6(37.5%) |
| HBV infection  Yes  No | 12(52.2%)  11(47.8%) | 12(75%)  4(25%) |
| Tumor size  <5 cm  ≥5 cm | 5(21.7%)  18(78.3%) | 10(62.5%)  6(37.5%) |
| Receiving anti-HBV treatment before resection |  |  |
| Entecavir | 0(0%) | 2(12.5%) |
| Adefovir Dipivoxil | 0(0%) | 1(6.3%) |
| Lamivudine | 1(4.3%) | 0(0%) |
| Recall lacking | 2(8.6%) | 0(0%) |
| No | 20(87%) | 13(81.3%) |
| Receiving non-resection treatment before resection |  |  |
| TACE | 1(4.3%) | 2(12.5%) |
| Microwave Ablation | 1(4.3%) | 0(0%) |
| No | 21(91.4%) | 14(87.5%) |
| Receiving any RTKs inhibitors treatment before resection |  |  |
| Yes | 0(0%) | 0(0%) |
| No | 23(100%) | 16(100%) |
| HCC metastasis existence before resection |  |  |
| Intrahepatic thrombus | 0(0%) | 1(6.3%) |
| Retroperitoneal | 0(0%) | 1(6.3%) |
| Common bile duct | 1(4.3%) | 0(0%) |
| No | 22(95.7%) | 14(87.5%) |
