# Supplementary material for: EGFR/MET promotes hepatocellular carcinoma metastasis by stabilizing tumor cells and resisting to RTKs inhibitors in circulating tumor microemboli
Source: Cell Death Dis. 2022 Apr 15;13(4):351. doi: 10.1038/s41419-022-04796-8 (PMC9012802; doi:10.1038/s41419-022-04796-8)
Supplement: Supplementary file 4 — Supplemental Table 2 [file 41419_2022_4796_MOESM4_ESM.docx]

|  | Liver | | | Lung | | |
| --- | --- | --- | --- | --- | --- | --- |
| GENE | Expressed in tumor | Expressed totally | Ratio | Expressed in tumor | Expressed totally | Ratio |
| **EGFR** | **4536** | **4722** | **0.96061** | **1780** | **2703** | **0.658528** |
| **MET** | **5143** | **5396** | **0.953113** | **1766** | **2174** | **0.812328** |
| AXL | 3536 | 5604 | 0.630978 | 1991 | 8497 | 0.234318 |
| **DDR1** | **2276** | **2641** | **0.861795** | **1259** | **1997** | **0.630446** |
| EPHA1 | 16 | 29 | 0.551724 | 3 | 28 | 0.107143 |
| **EPHA2** | **9029** | **9507** | **0.949721** | **3394** | **4141** | **0.819609** |
| EPHA3 | 0 | 14 | 0 | 3 | 114 | 0.026316 |
| EPHA4 | 10 | 41 | 0.243902 | 30 | 285 | 0.105263 |
| EPHA5 | 2 | 6 | 0.333333 | 5 | 8 | 0.625 |
| EPHB1 | 0 | 6 | 0 | 0 | 37 | 0 |
| EPHB2 | 148 | 159 | 0.930818 | 53 | 318 | 0.166667 |
| EPHB3 | 66 | 75 | 0.88 | 113 | 262 | 0.431298 |
| **EPHB4** | **5616** | **6007** | **0.934909** | **2236** | **2613** | **0.855721** |
| EPHB6 | 847 | 914 | 0.926696 | 378 | 485 | 0.779381 |
| **ERBB2** | **1031** | **1085** | **0.95023** | **489** | **632** | **0.773734** |
| ERBB3 | 174 | 271 | 0.642066 | 192 | 306 | 0.627451 |
| ERBB4 | 0 | 6 | 0 | 0 | 3 | 0 |
| **FGFR1** | **3603** | **4064** | **0.886565** | **1512** | **2961** | **0.510638** |
| FGFR2 | 165 | 199 | 0.829146 | 79 | 265 | 0.298113 |
| FGFR3 | 135 | 230 | 0.586957 | 96 | 280 | 0.342857 |
| **FGFR4** | **1257** | **1326** | **0.947964** | **555** | **606** | **0.915842** |
| FLT1 | 119 | 1597 | 0.074515 | 108 | 2701 | 0.039985 |
| FLT3 | 31 | 882 | 0.035147 | 26 | 890 | 0.029213 |
| FLT4 | 1415 | 2320 | 0.609914 | 916 | 1255 | 0.72988 |
| IGF1R | 20 | 761 | 0.026281 | 45 | 2199 | 0.020464 |
| IGF2R | 660 | 1477 | 0.446852 | 464 | 2272 | 0.204225 |
| INSR | 2362 | 3133 | 0.75391 | 1022 | 2073 | 0.493005 |
| KDR | 32 | 2086 | 0.01534 | 17 | 707 | 0.024045 |
| MCSFR | 186 | 3752 | 0.049574 | 228 | 7354 | 0.031004 |
| MST1R | 22 | 23 | 0.956522 | 65 | 161 | 0.403727 |
| MUSK | 11 | 12 | 0.916667 | 28 | 39 | 0.717949 |
| NGFR | 45 | 77 | 0.584416 | 431 | 499 | 0.863727 |
| PDGFRA | 191 | 235 | 0.812766 | 119 | 2844 | 0.041842 |
| PDGFRB | 5 | 200 | 0.025 | 23 | 1477 | 0.015572 |
| RET | 0 | 28 | 0 | 2 | 73 | 0.027397 |
| ROR1 | 463 | 496 | 0.933468 | 360 | 755 | 0.476821 |
| ROR2 | 41 | 47 | 0.87234 | 110 | 263 | 0.418251 |
| **RYK** | **3315** | **3880** | **0.854381** | **1766** | **2935** | **0.601704** |
| TIE1 | 11 | 345 | 0.031884 | 15 | 435 | 0.034483 |
