# Supplementary material for: EGFR/MET promotes hepatocellular carcinoma metastasis by stabilizing tumor cells and resisting to RTKs inhibitors in circulating tumor microemboli
Source: Cell Death Dis. 2022 Apr 15;13(4):351. doi: 10.1038/s41419-022-04796-8 (PMC9012802; doi:10.1038/s41419-022-04796-8)
Supplement: Supplementary file 5 — Supplemental Table 3 [file 41419_2022_4796_MOESM5_ESM.docx]

| Antibody For Western Blot | | |
| --- | --- | --- |
| Assay | Enterprise | Detail |
| EGFR | Abcam | ab52894 |
| pEGFR | CST | 3777T |
| MET | CST/Abcam | 8198T/ab51067 |
| pMET | CST | 3077T |
| pHer3 | Abcam | ab255607 |
| pMST1R | Abcam | Ab124671 |
| pIGF1R/pInsR | CST | 3021T |
| pRET | Abcam | ab51103 |
| pFGFR | Abcam | ab173305 |
| pVEGFR2 | CST | 2478T |
| pPDGFRβ | Abcam | ab218534 |
| MEK | Abcam | ab178876 |
| p-MEK | CST | 9154T |
| ERK | Abcam | ab184699 |
| p-ERK | CST | 4370T |
| Ras | CST | 3339T |
| CDK4 | CST | 12790T |
| CyclinA | CST | 4656T |
| CyclinB | CST | 12231T |
| CyclinD | CST | 2978T |
| CyclinE | CST | 4129T |
| C-PARP | Abcam | ab32064 |
| B-Actin | ProteinTech | Cat No. 66009-1-Ig |
| GAPDH | ProteinTech | Cat No. 60004-1-Ig |
| Antibody For Co-immunoprecipitation | | |
| Assay | Enterprise | Detail |
| EGFR | santa | sc-373746 |
| MET | CST | sc-8057 |
| IgG | santa(M)/CST(R) | sc-2025/3900S |
| p-EGFR | CST | 3777T |
| pMET | CST | 3077T |
| Antibody For Immunofluorescence | | |
| CD45 | Abcam | 52894 |
| EpCAM | Abcam | 187372 |
| Antibody For Immunohistochemistry | | |
| EGFR | santa | sc-373746 |
| MET | santa | sc-514148 |
