# Supplementary material for: EGFR/MET promotes hepatocellular carcinoma metastasis by stabilizing tumor cells and resisting to RTKs inhibitors in circulating tumor microemboli
Source: Cell Death Dis. 2022 Apr 15;13(4):351. doi: 10.1038/s41419-022-04796-8 (PMC9012802; doi:10.1038/s41419-022-04796-8)
Supplement: Supplementary file 6 — Supplemental Table 4 [file 41419_2022_4796_MOESM6_ESM.docx]

| Gene | Target Sequence |
| --- | --- |
| EGFR | GCAGUCUUAUCUAACUAUGAUGCAA |
| MET SiRNA1 | CUGGUUUUGUCGACGUAAA |
| MET SiRNA2 | CGAGGGAAUCAUCAUGAAA |
