# Supplementary material for: EGFR/MET promotes hepatocellular carcinoma metastasis by stabilizing tumor cells and resisting to RTKs inhibitors in circulating tumor microemboli
Source: Cell Death Dis. 2022 Apr 15;13(4):351. doi: 10.1038/s41419-022-04796-8 (PMC9012802; doi:10.1038/s41419-022-04796-8)
Supplement: Supplementary file 8 — Author Contribution Form [file 41419_2022_4796_MOESM8_ESM.pdf]

DECLARATION OF CONTRIBUTIONS TO ARTICLE

ADMC

Manuscript Number: CDDIS-21-4457

Journal Name: *Cell Death & Disease* (the 'Journal')

Proposed Title of the Contribution:

EGFR/MET promotes hepatocellular carcinoma metastasis by stabilizing tumor cells and resisting to RTKs inhibitors in circulating tumor microemboli (the 'Contribution)

Author(s):

Shouyang Song, Zhen Yu, Yajing You, Chenxi Liu, Xiaoyu Xie, Huanran Lv, Feng Xiao, Qiang Zhu*, Chengyong Qin* (the 'Authors')

For all *CDDis* articles, each person named as an author in the published version must be able to show he or she has contributed substantially to the article.

Authorship credit should be based on 1) substantial contributions to conception and design, acquisition of data, or analysis and interpretation of data; 2) drafting the article or revising it critically for important intellectual content; and 3) final approval of the version to be published. Authors should meet conditions 1, 2 and 3.

Any person who cannot be shown to have made a substantial contribution to the article cannot be listed as an author in the final version. The name of any person who is deemed to have made a minor contribution can, however, appear in the Acknowledgments section of the article.

Please complete the table below to indicate the contributions of all named authors to the manuscript.

Author Full Name:	Specification of Contribution to the Manuscript:
Shouyang Song	performed the major experiment and statistical analysis.
Zhen Yu	established mice xenograft model.
Yajing You	technical and material support.
Chenxi Liu	technical and material support.
Xiaoyu Xie	technical and material support.
Huanran Lv	technical and material support.
Feng Xiao	technical and material support.
Qiang Zhu	performed methodology and guided writing, review of the paper.
Chengyong Qin	performed study concept and design.

Please complete the table below to indicate the contributions of all named authors to the figures.

Figure 1:

SY.S performed Western Blot, LumineX, Elisa, and statistical analysis. YJ.Y F.X and XY.X provided patients HCC tissues.

Figure 2:

Z.Y established mice model, CX.L contected sq-RNA for RNA sequence. SY.S performed IHC, IF and statistical analysis.

Figure 3:

HR.L established low-attactment culture model and provided donor blood sample, SY.S performed Western Blot, Elisa and IF.

Figure 4:

HR.L contacted for Ras-GTP assays. SY.S performed all Western Blot.

Figure 5:

SY.S performed Western Blot, FACS, and IF.

Figure 6:

SY.S performed all Western Blot.

Signed for and on behalf of the Author(s):	Print Name:	Date:
	Chengyong Qin	15 Feb, 2022
