# Supplementary material for: EGFR/MET promotes hepatocellular carcinoma metastasis by stabilizing tumor cells and resisting to RTKs inhibitors in circulating tumor microemboli
Source: Cell Death Dis. 2022 Apr 15;13(4):351. doi: 10.1038/s41419-022-04796-8 (PMC9012802; doi:10.1038/s41419-022-04796-8)
Supplement: Supplementary file 9 — Western Blot Origin [file 41419_2022_4796_MOESM9_ESM.docx]

Origin Western Blot

Note:

-The pictures are sorted by figure, and in each figure, they are sorted from top to bottom, left to right.


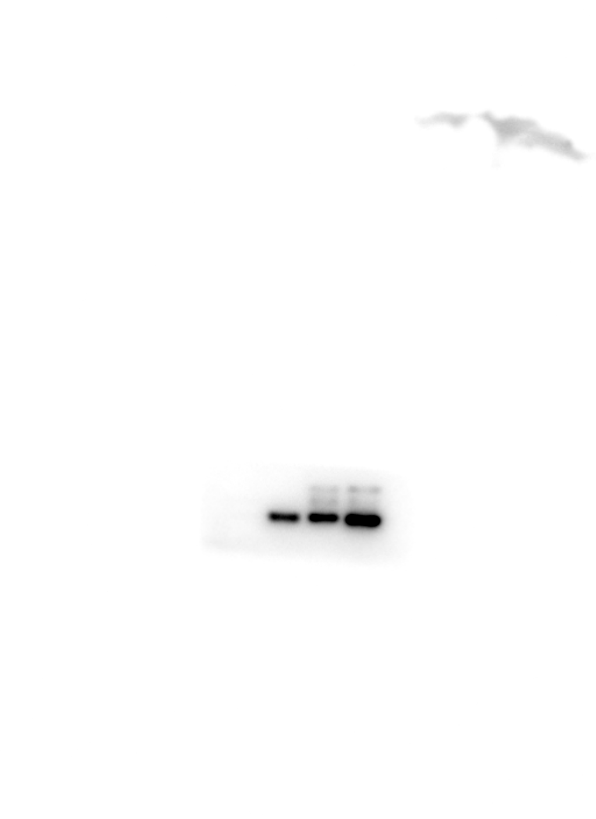

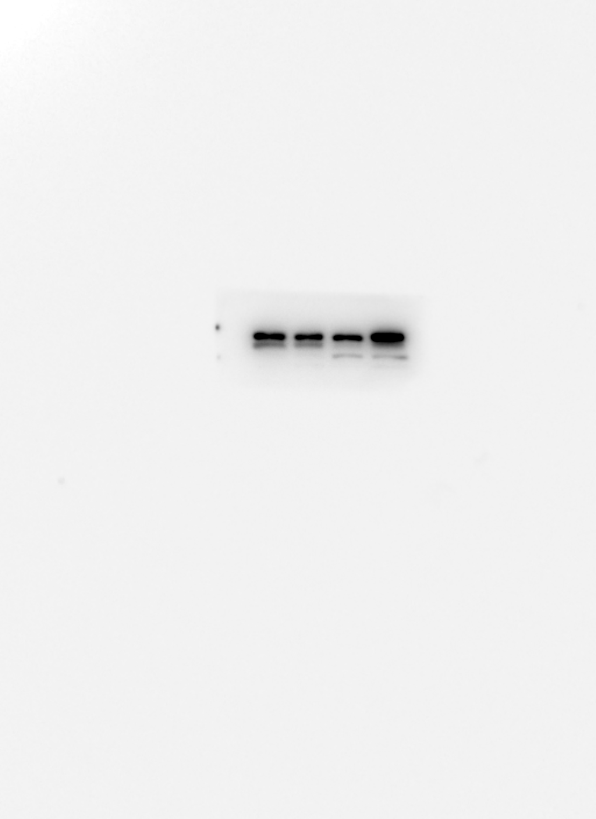

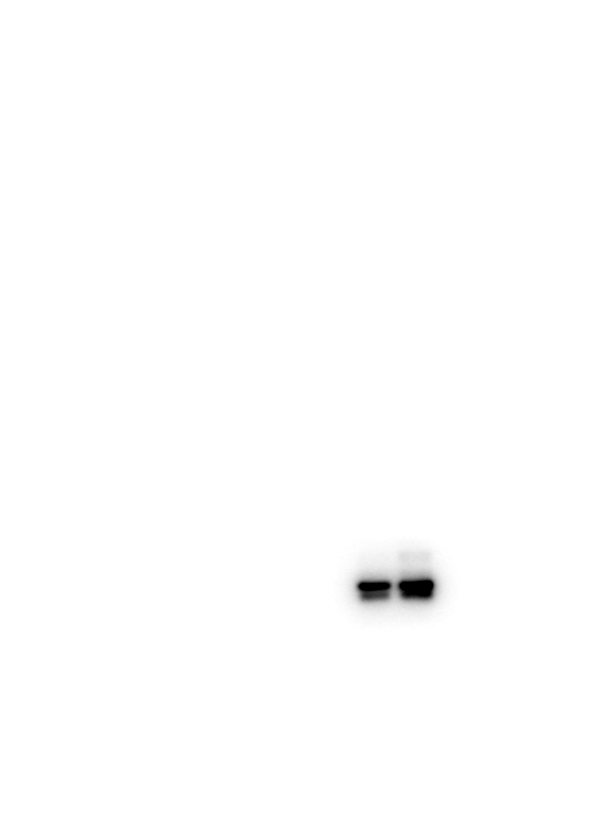

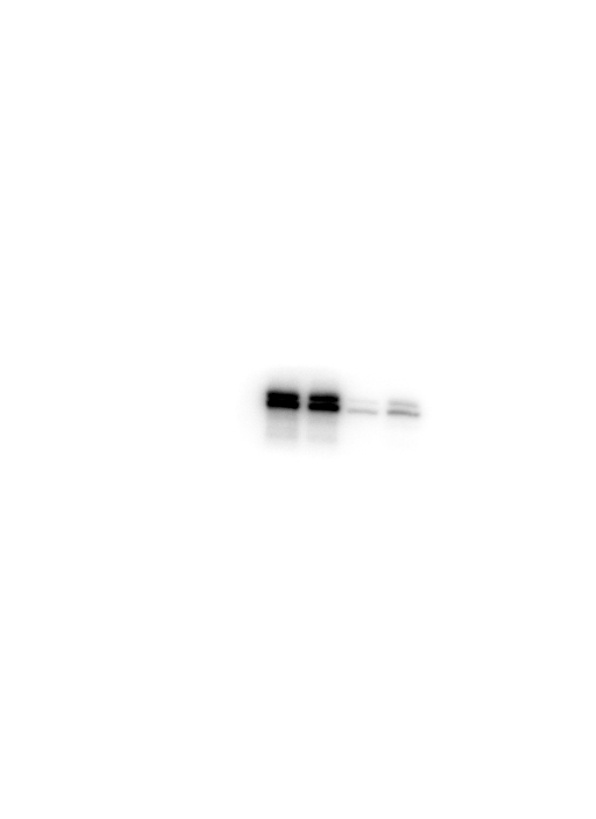

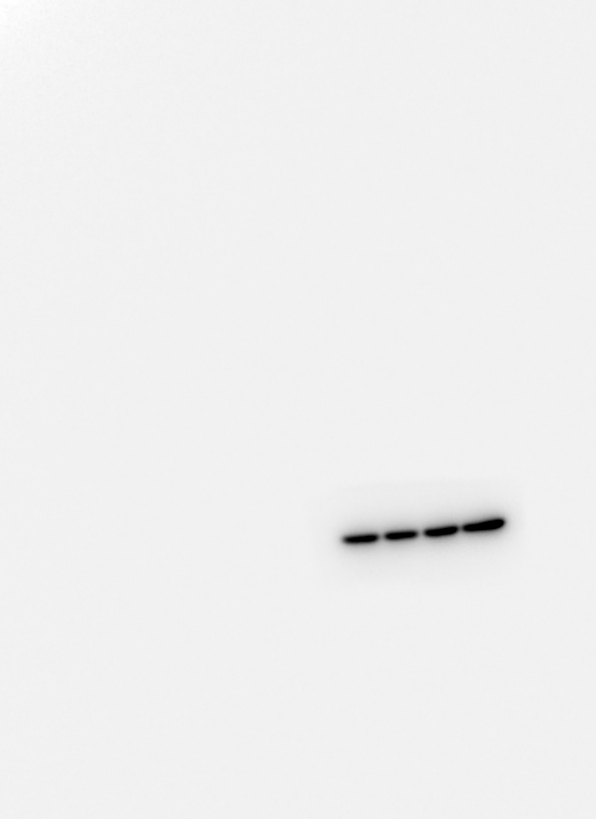


Figure 1.B


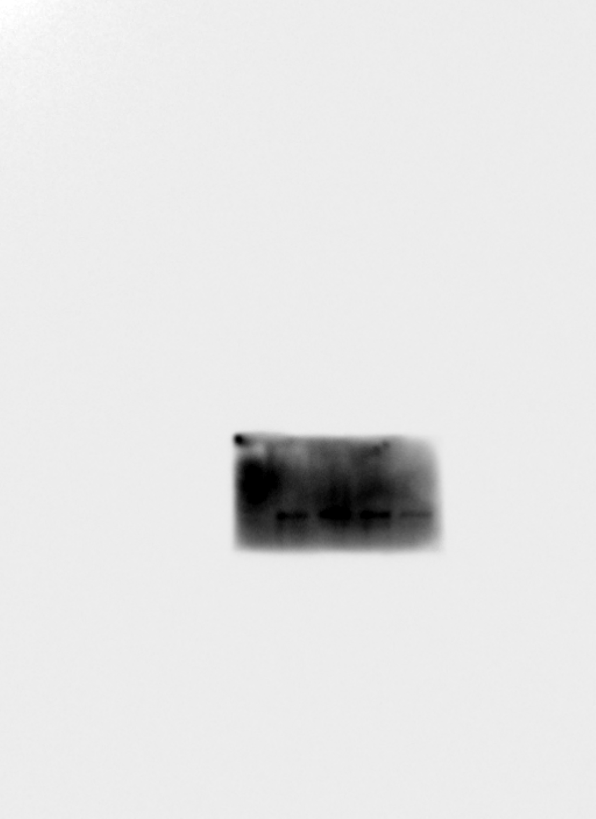

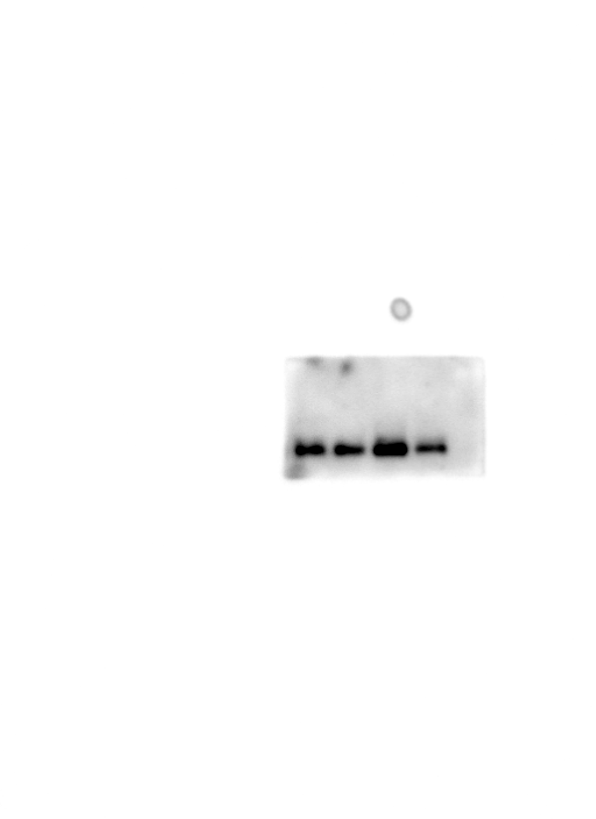

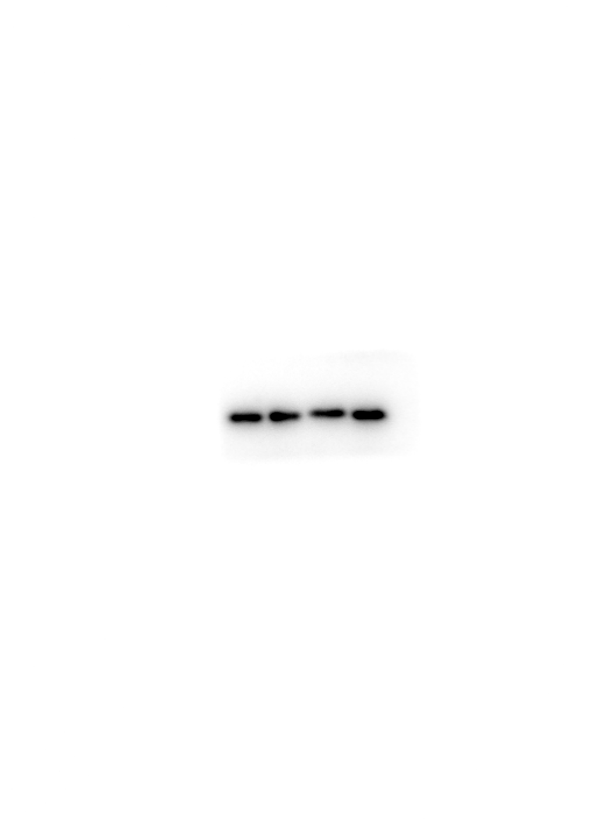

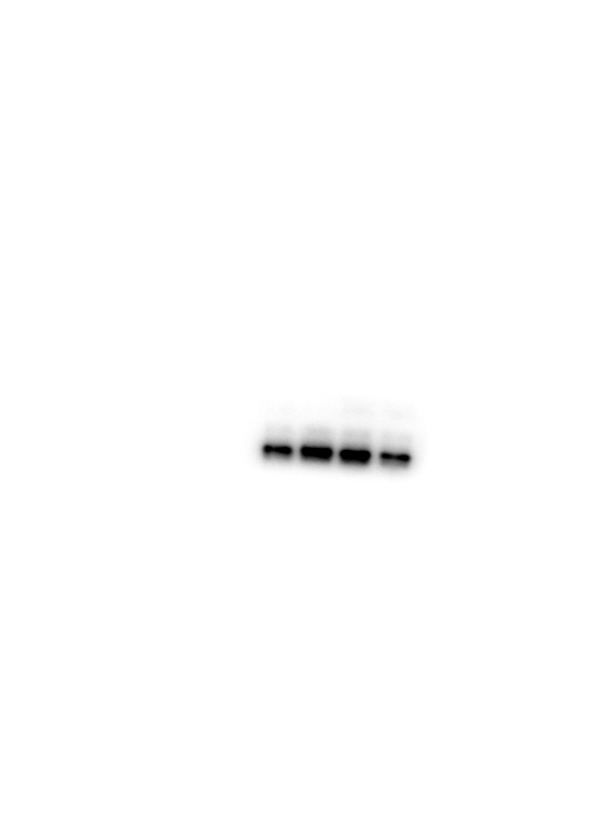

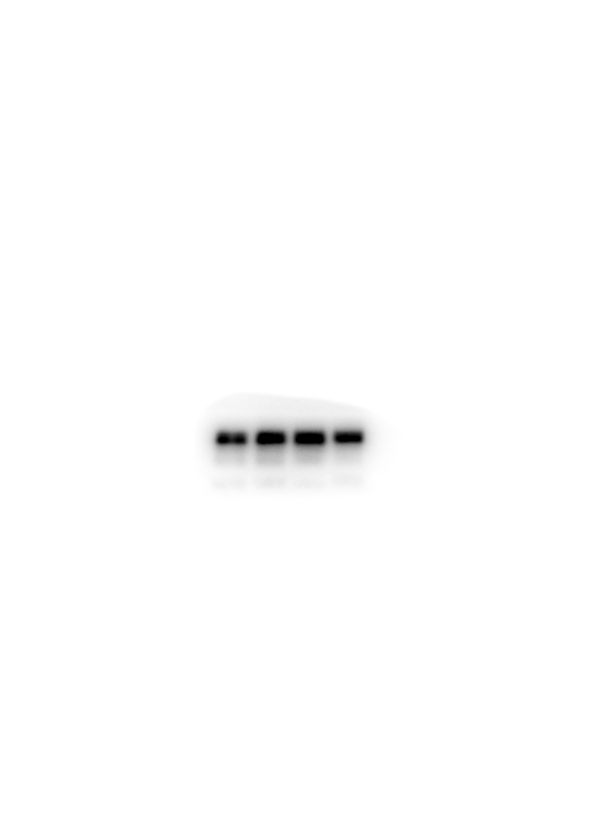

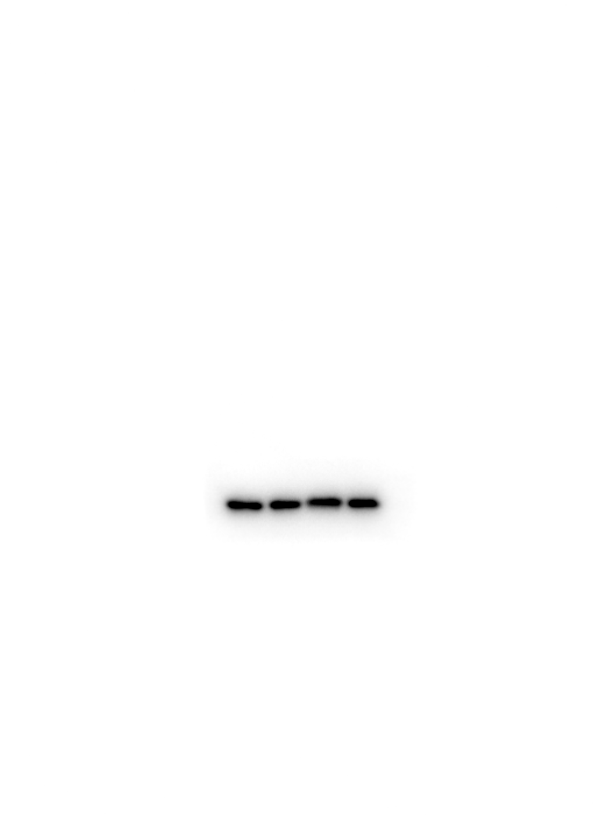

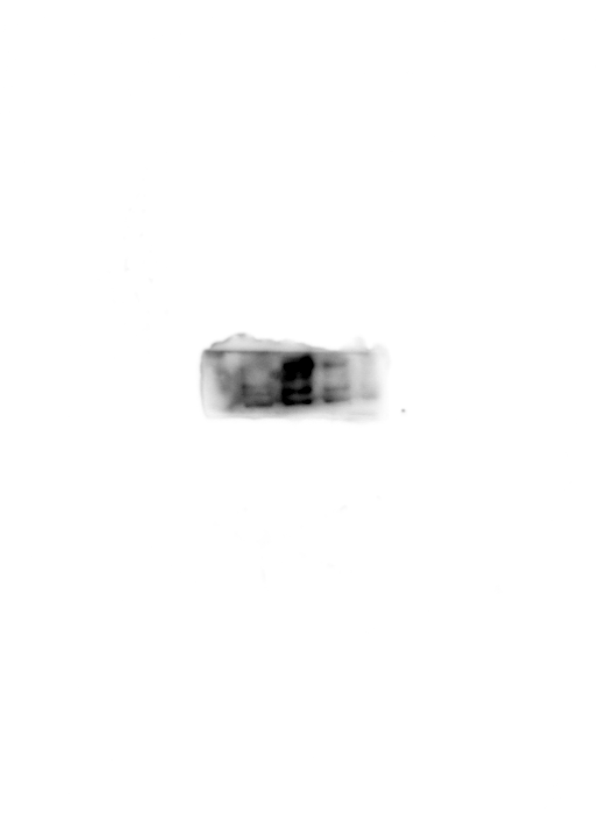

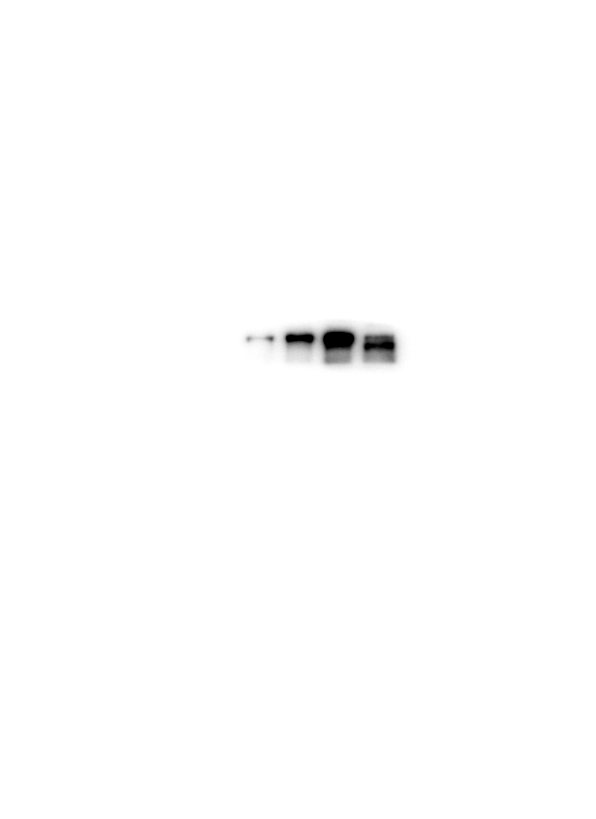

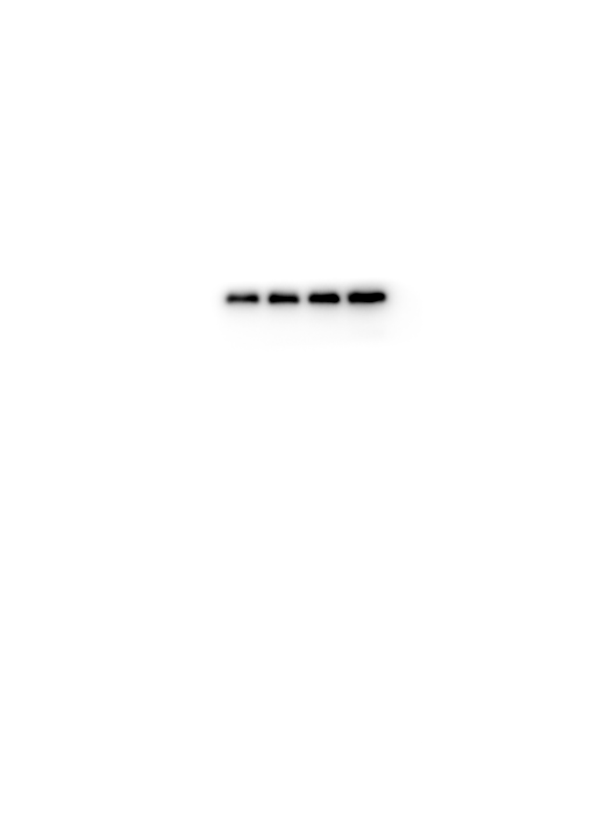

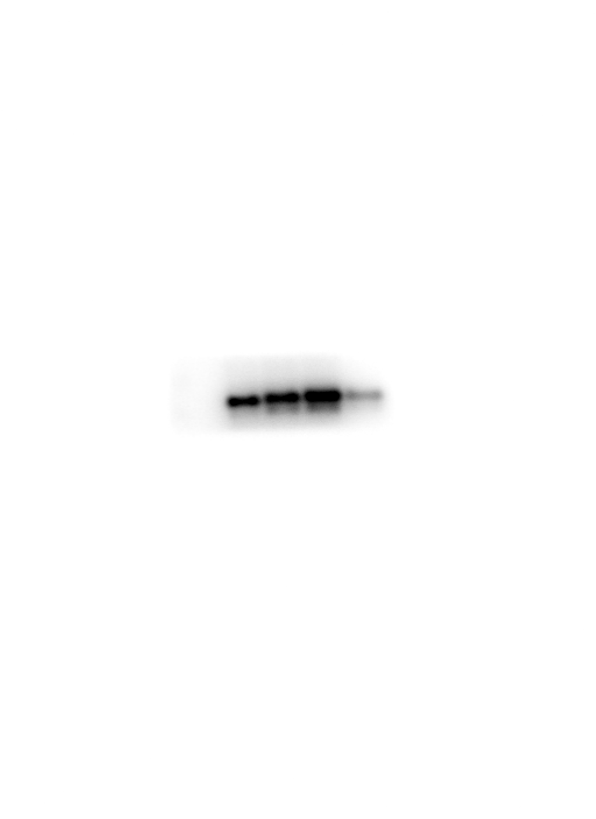

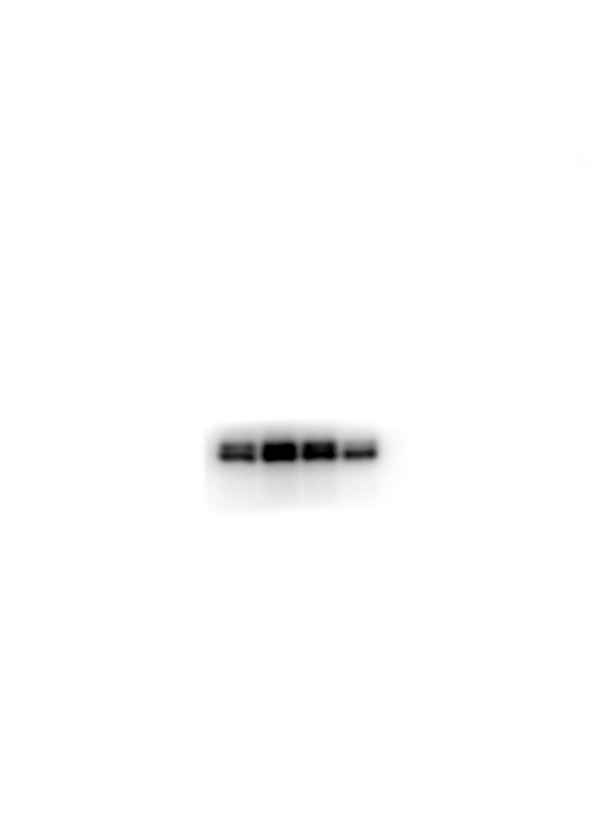

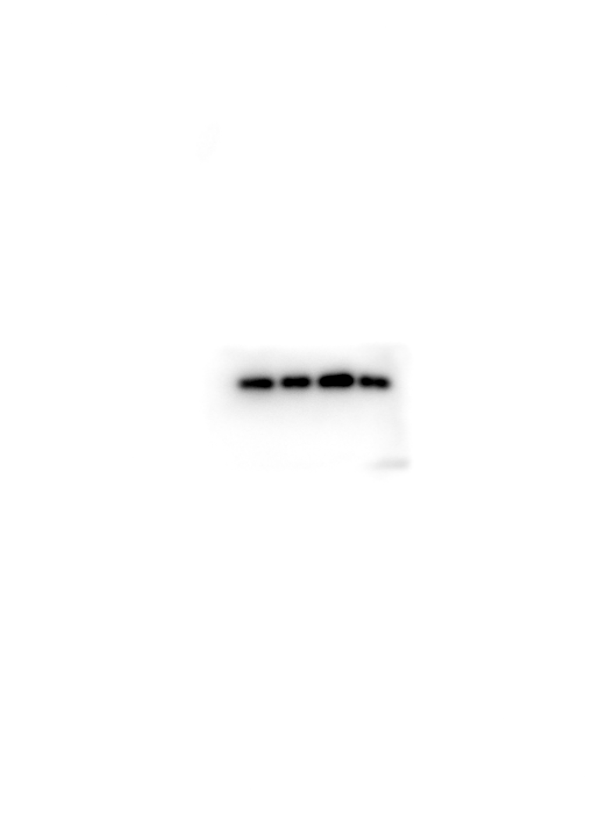

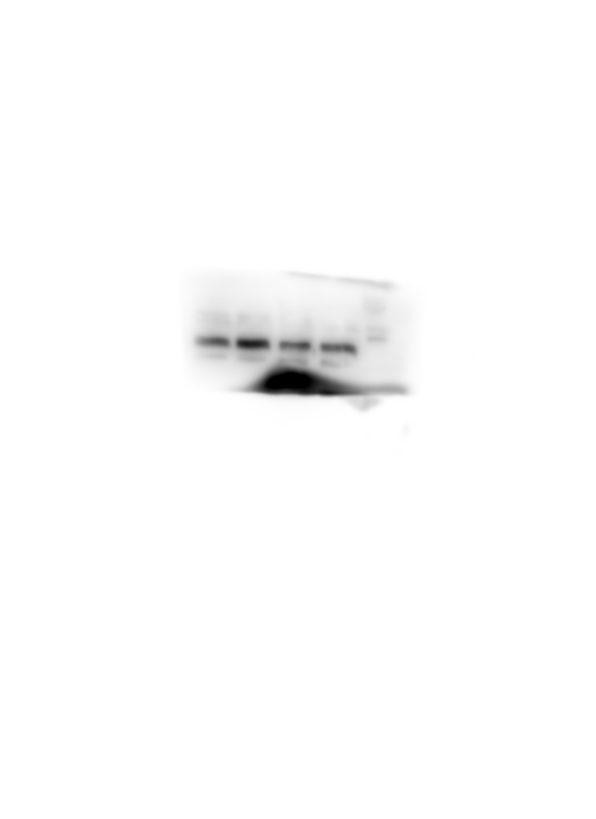

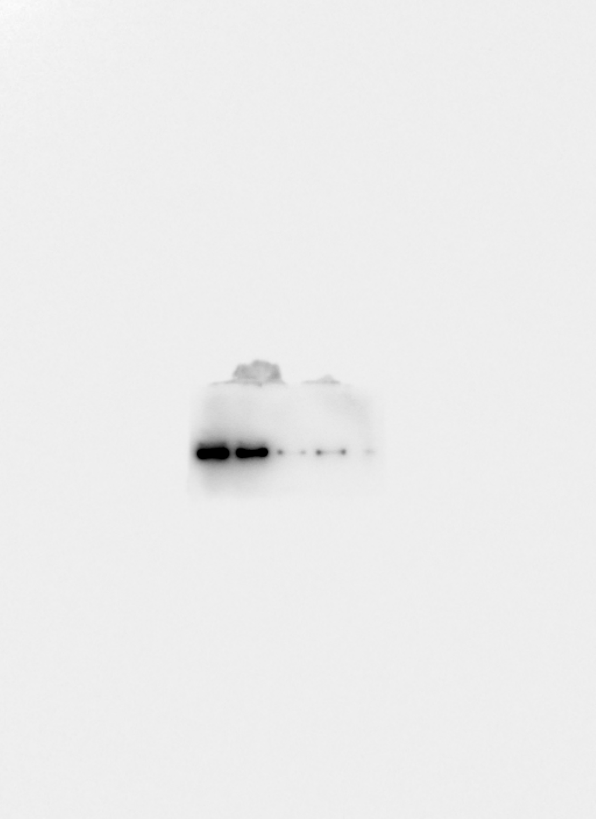

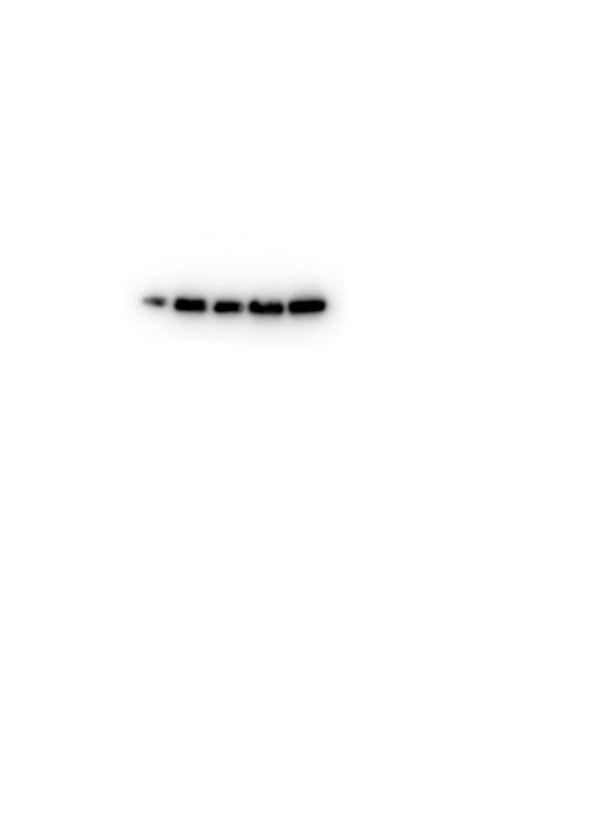

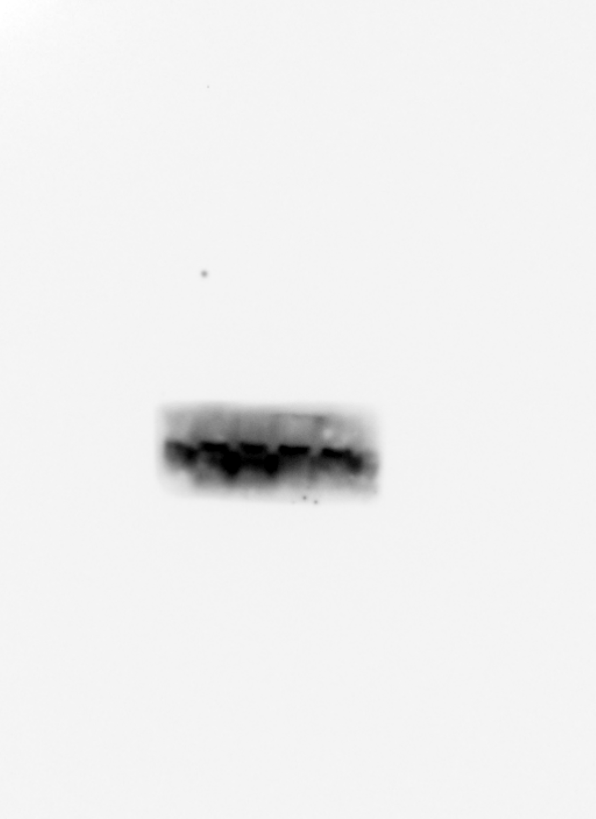

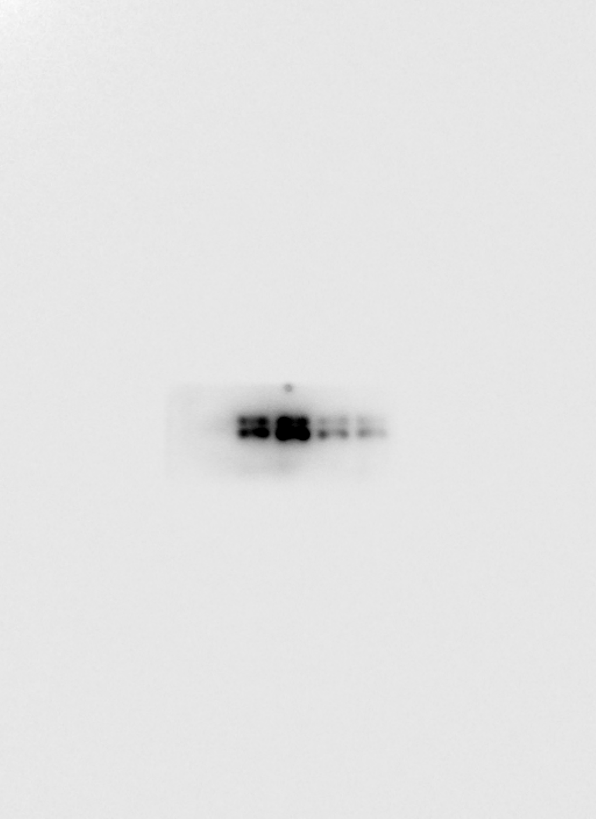

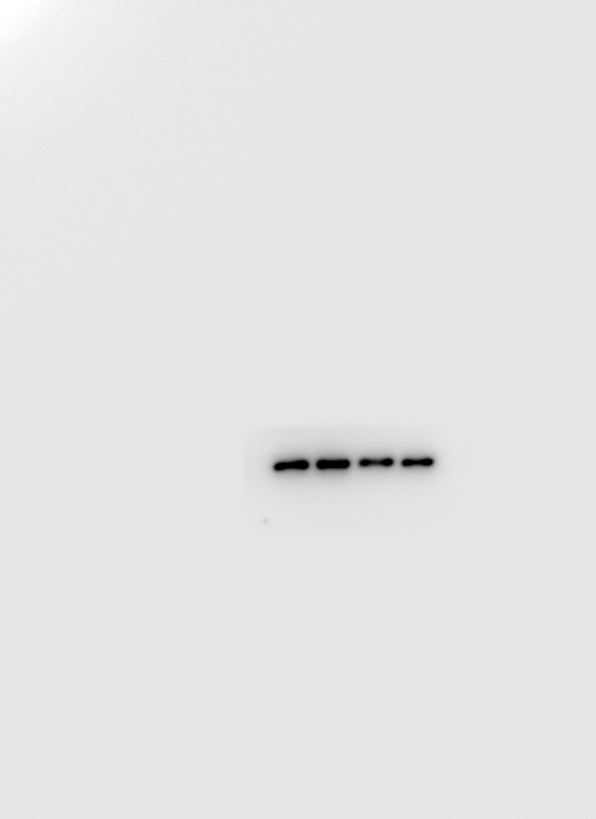

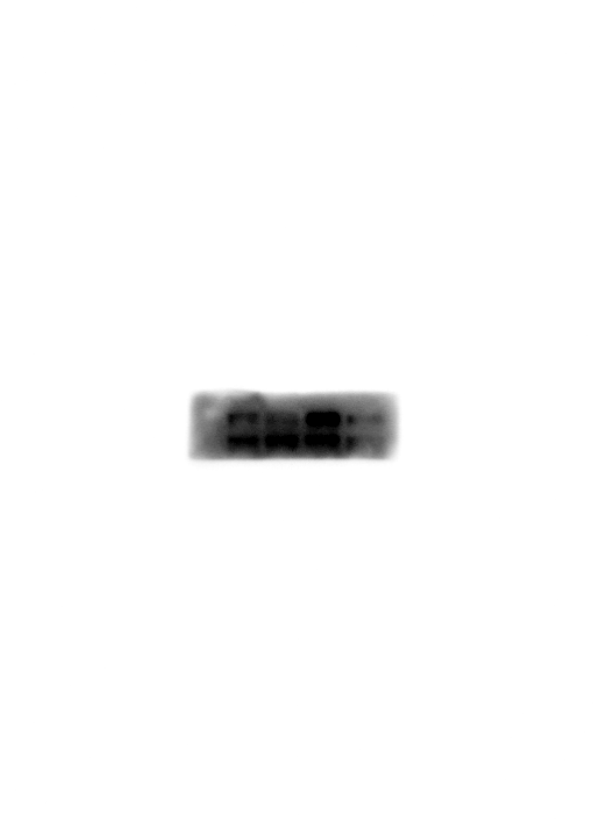

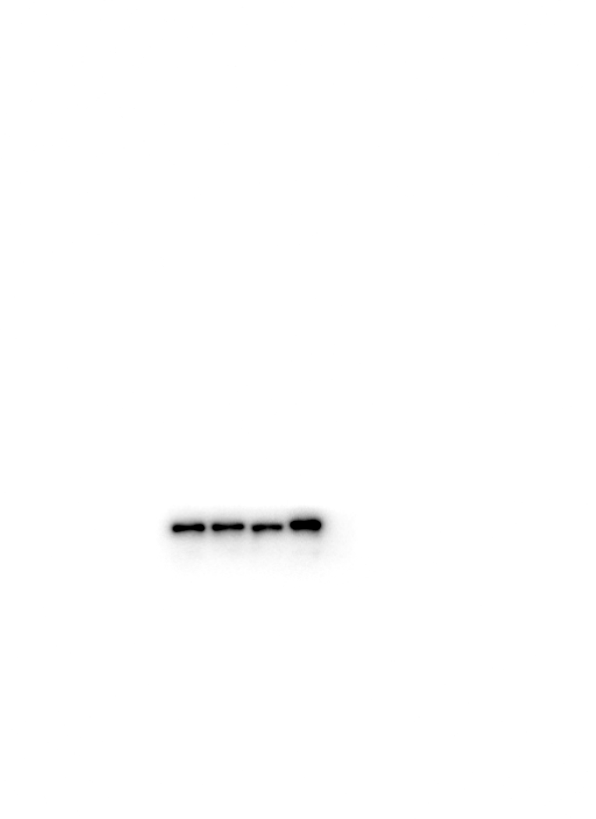

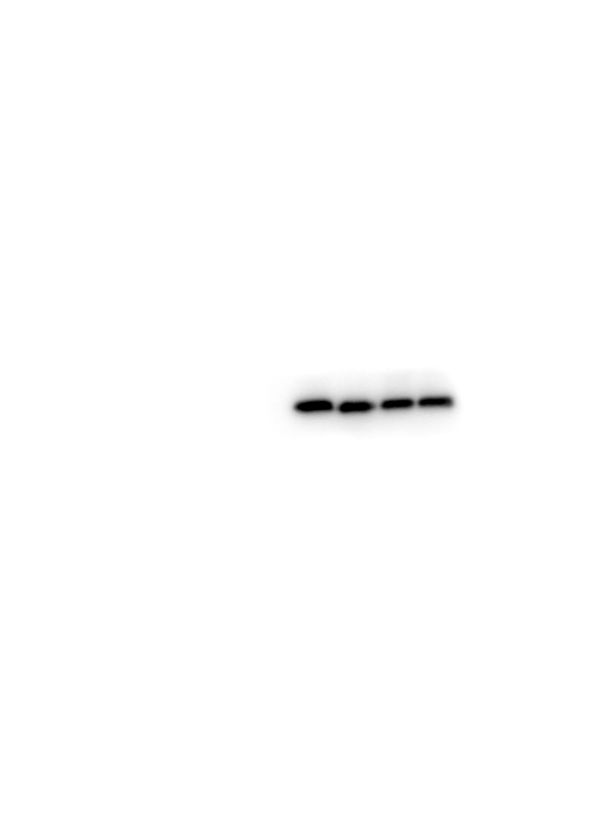

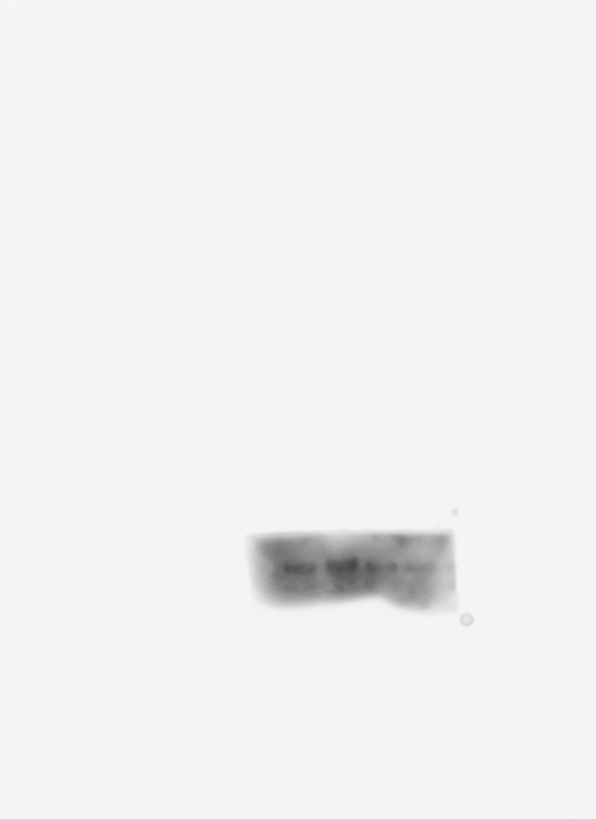

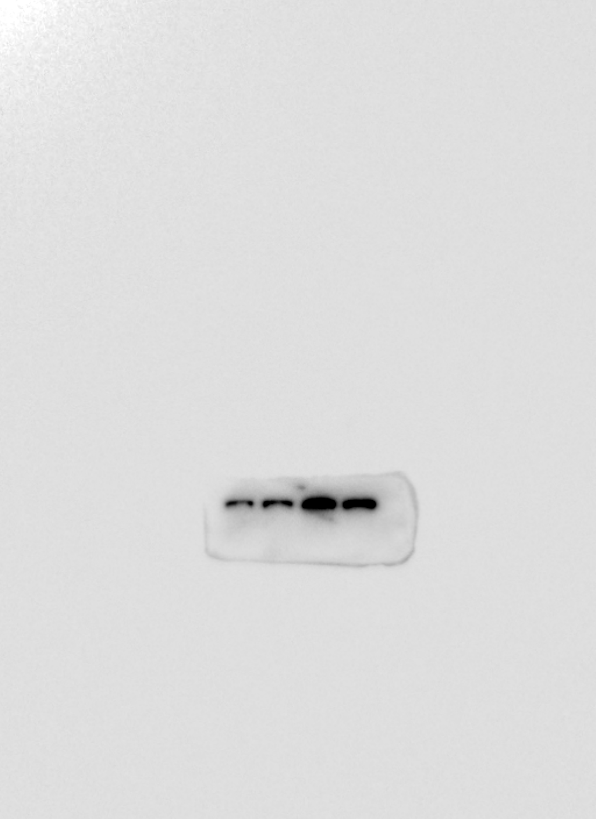

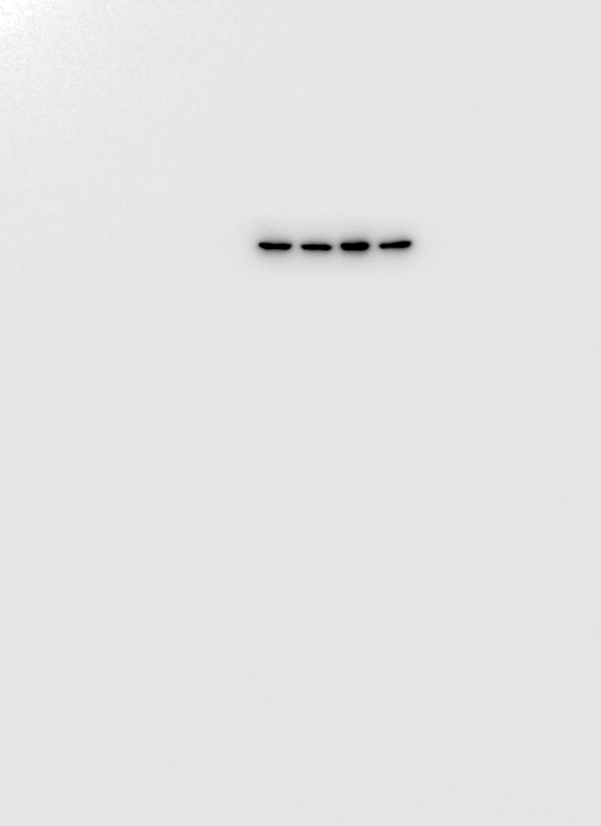


Figure 3.B


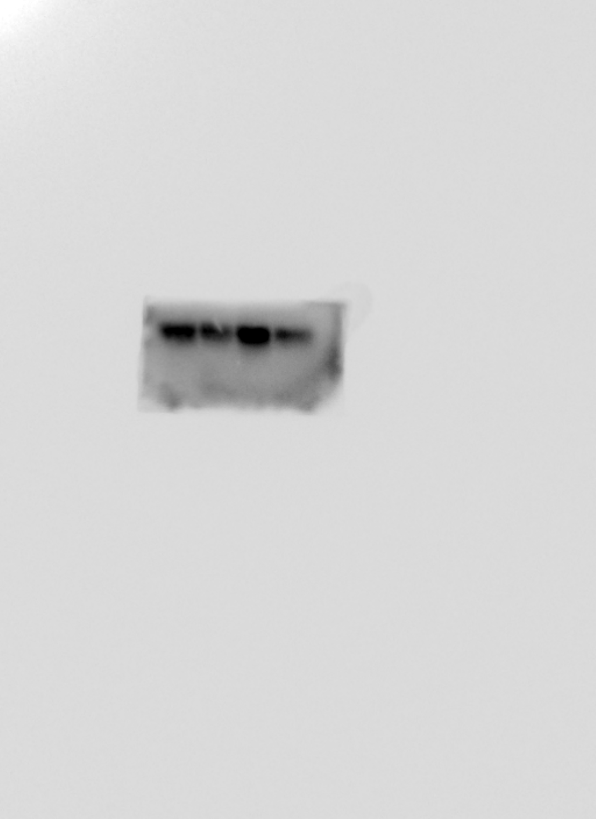

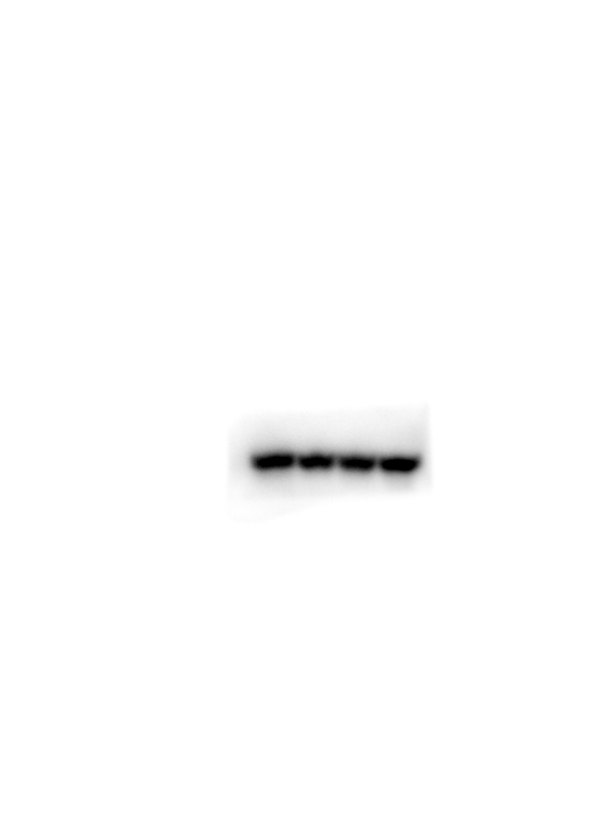

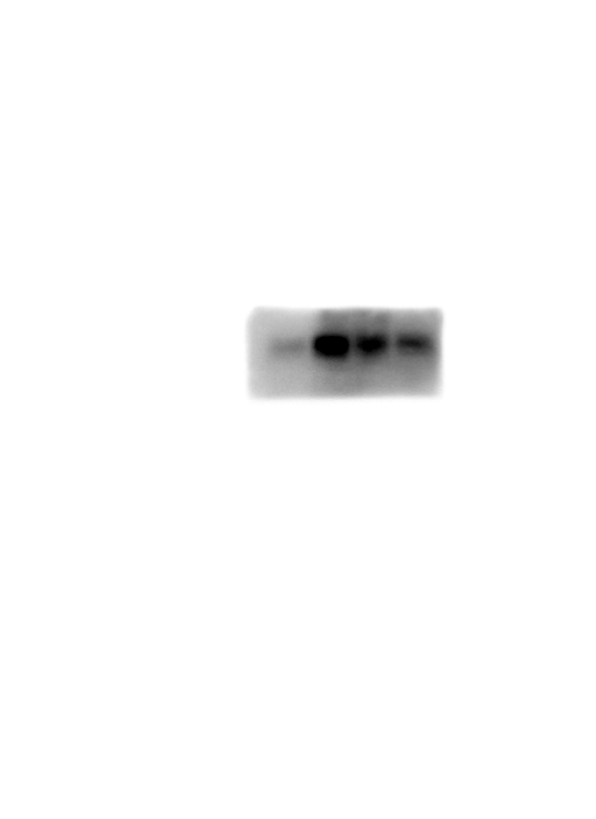

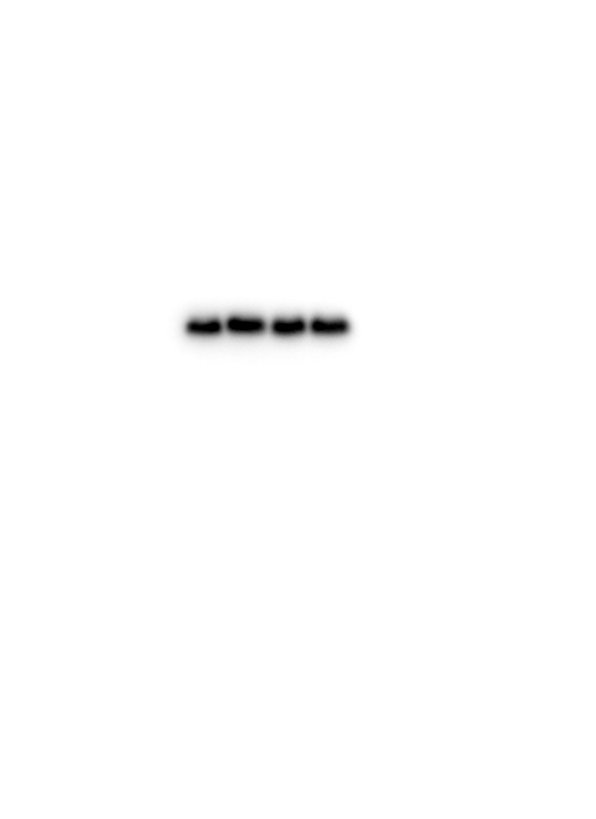

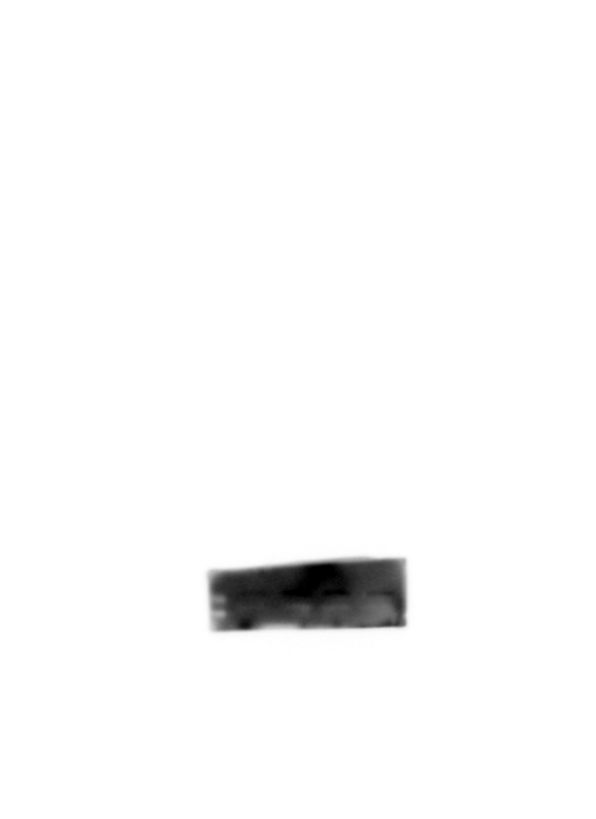

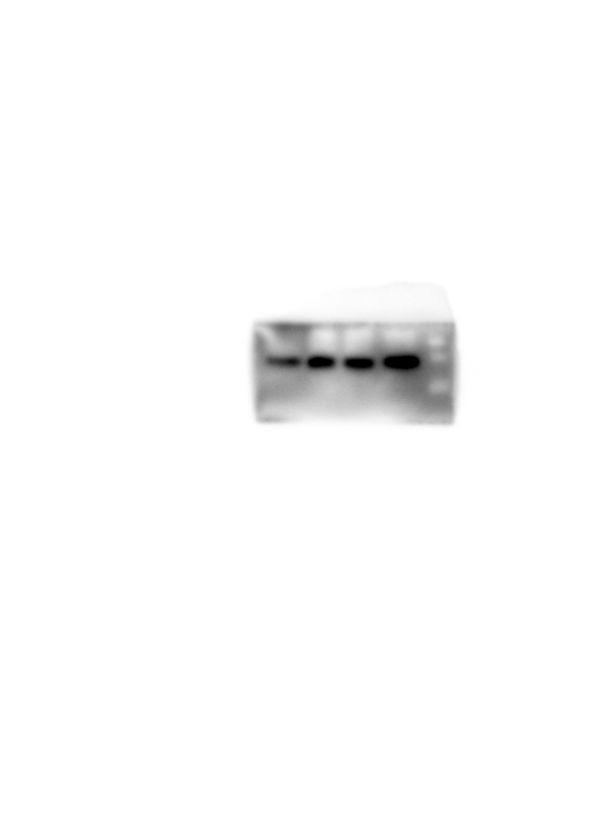

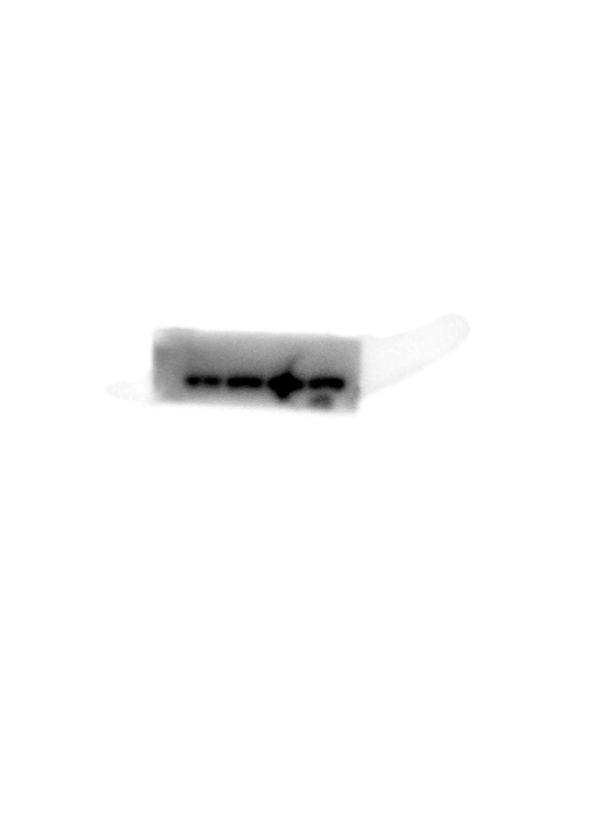

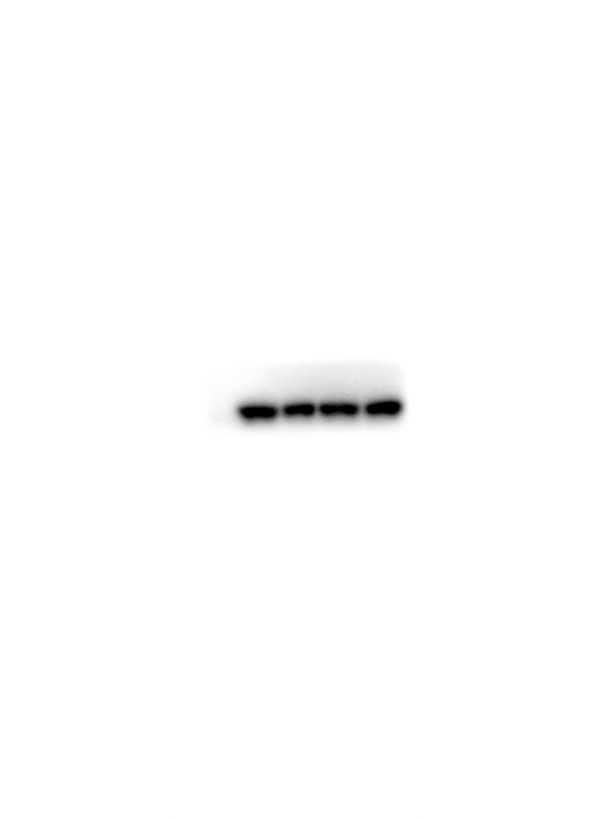


Figure 4.A


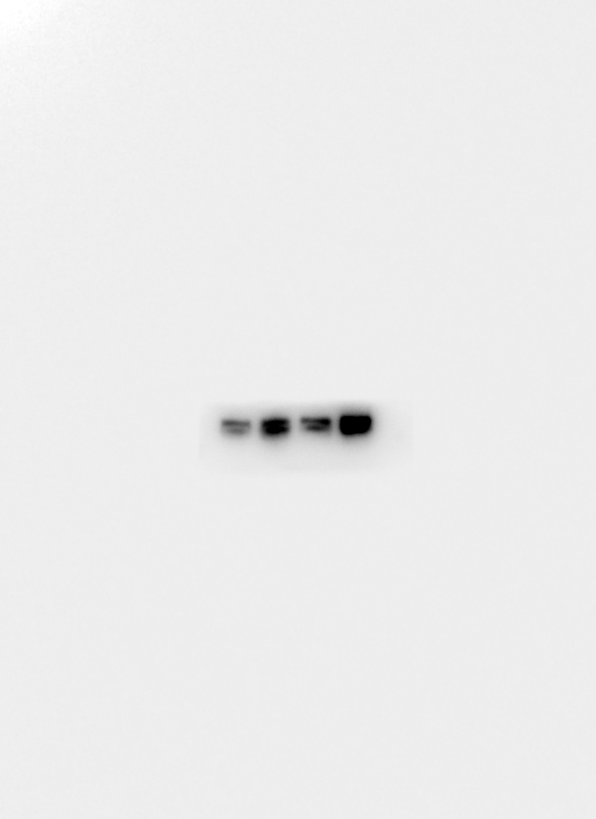

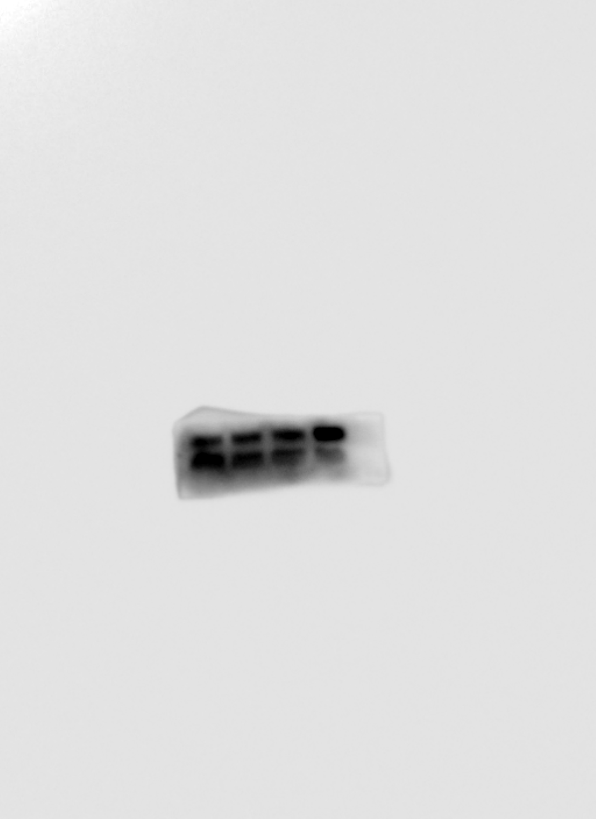

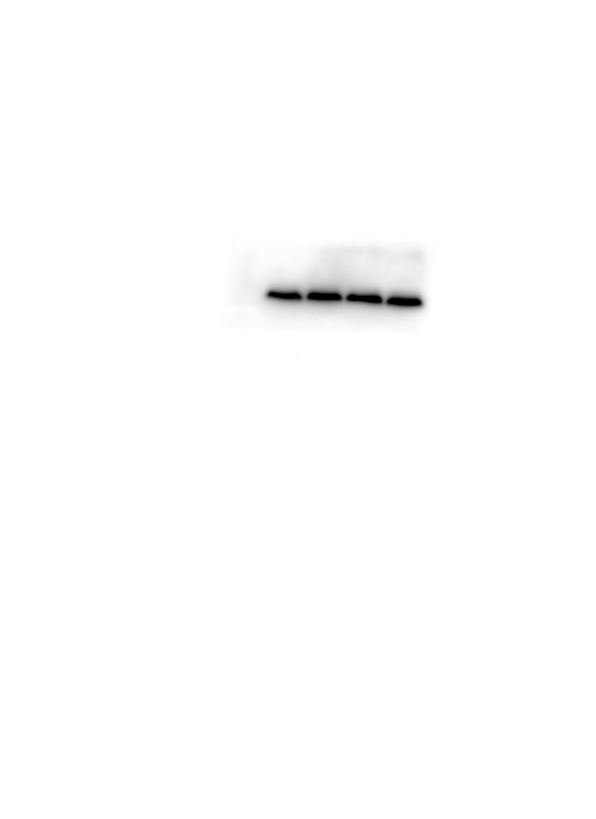

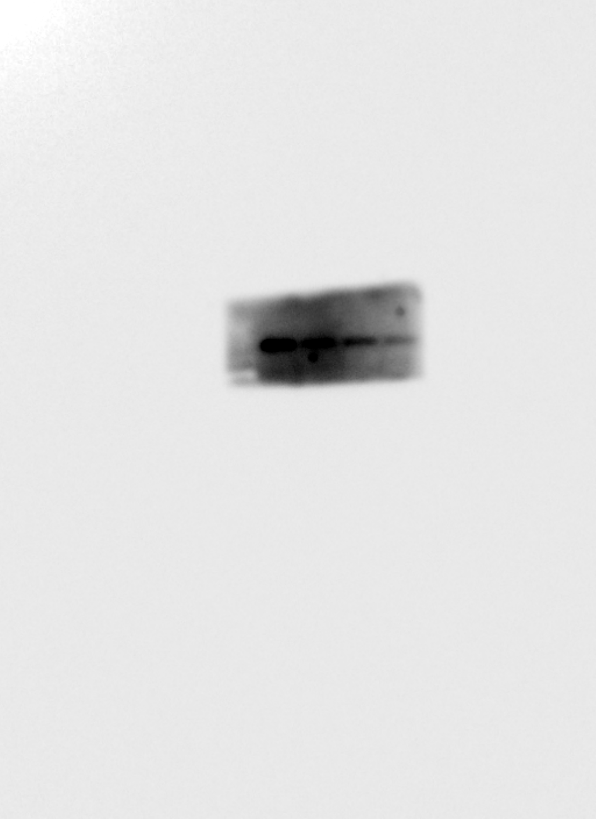

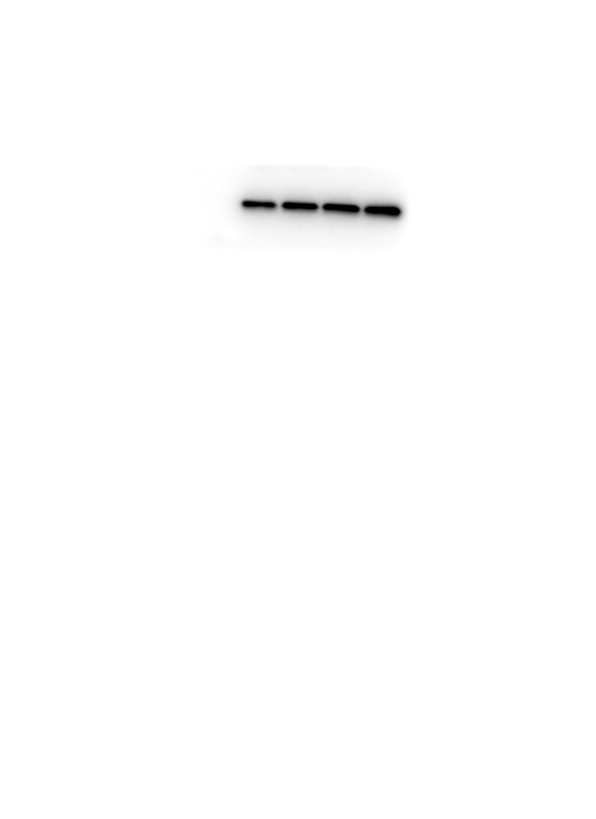

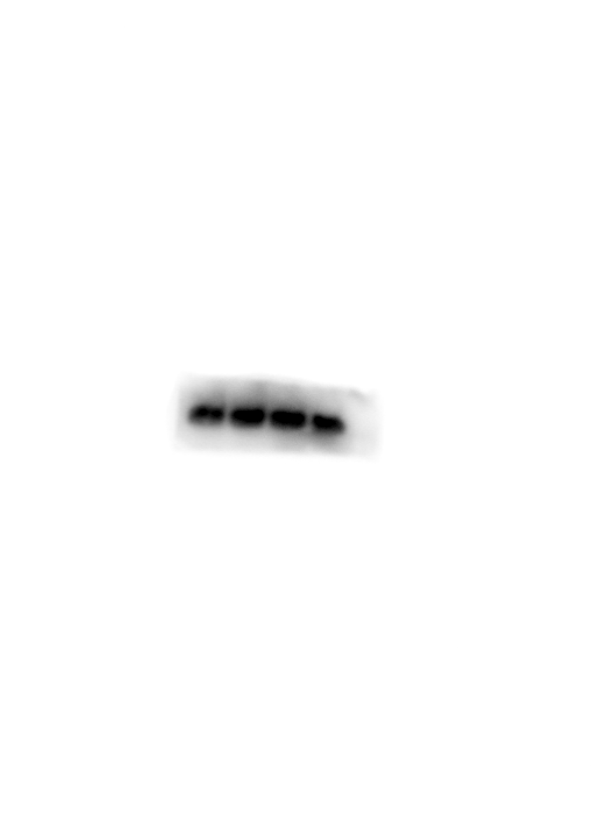

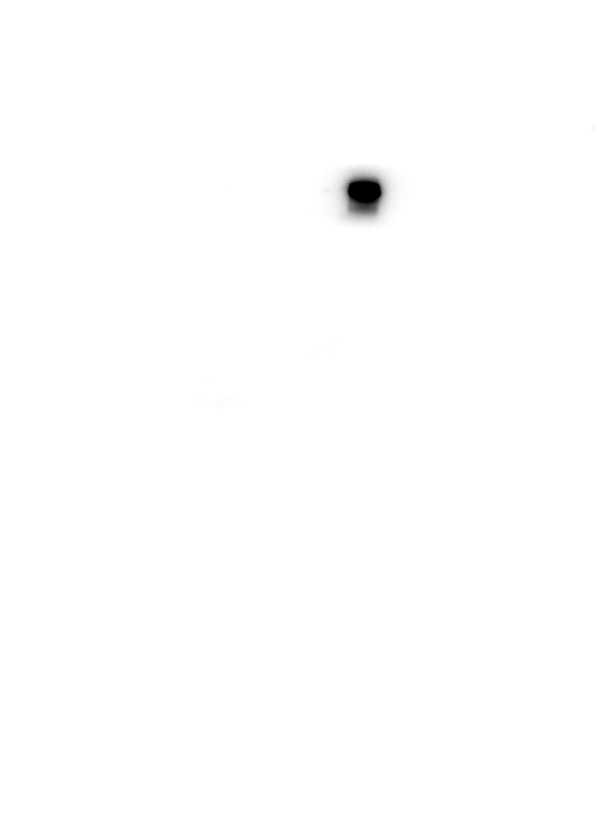

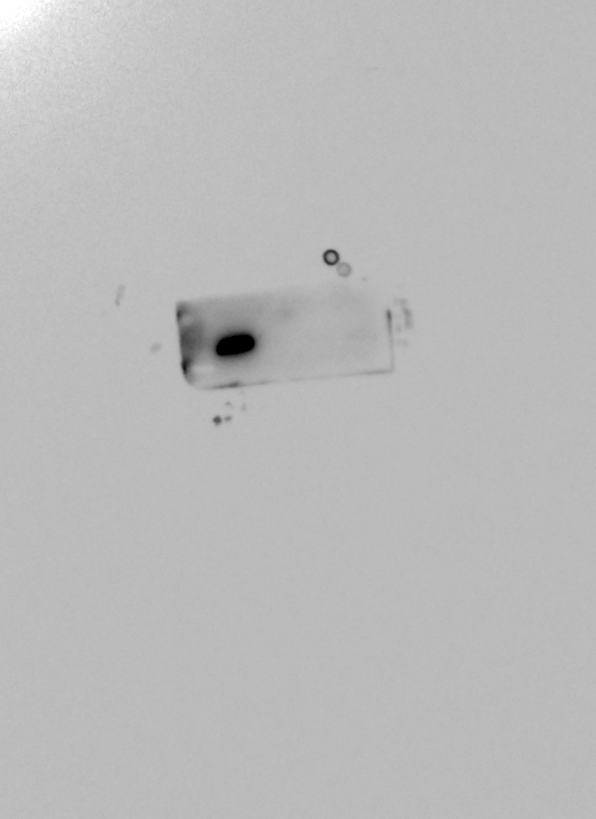

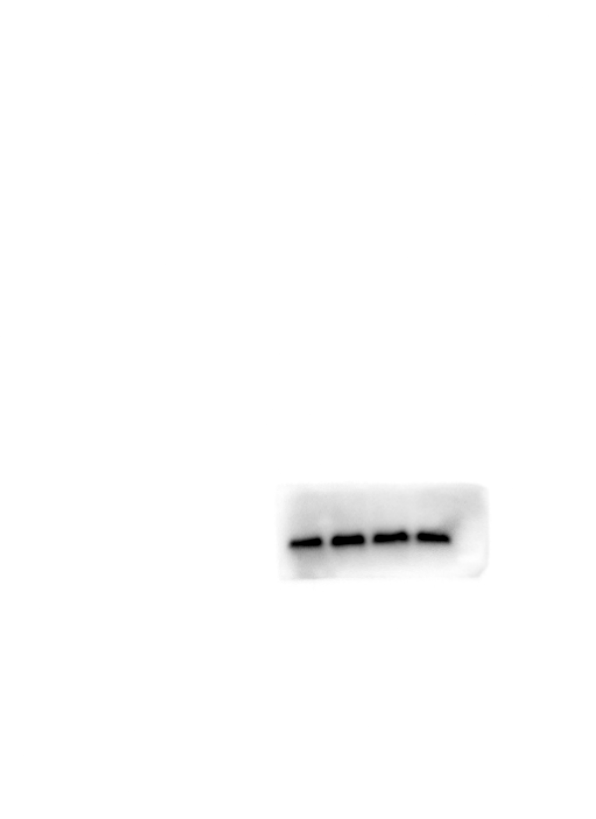

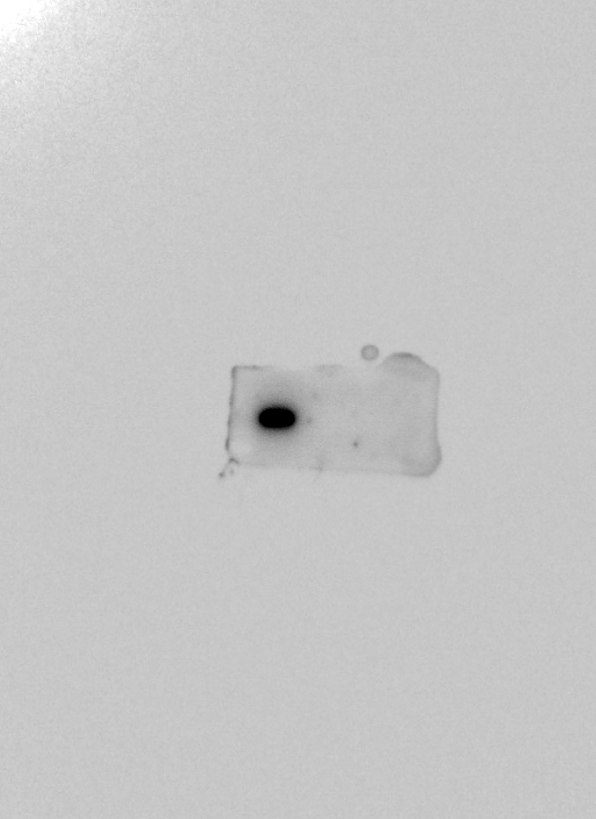

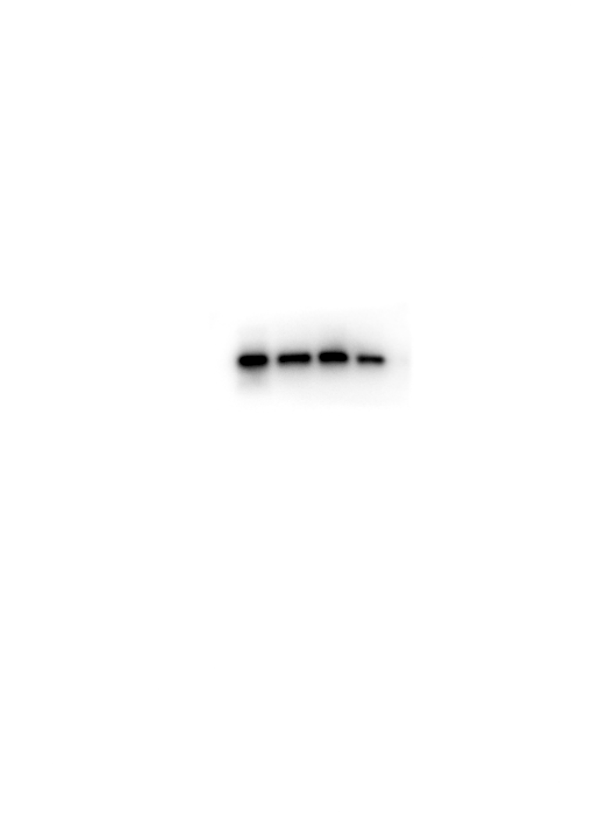

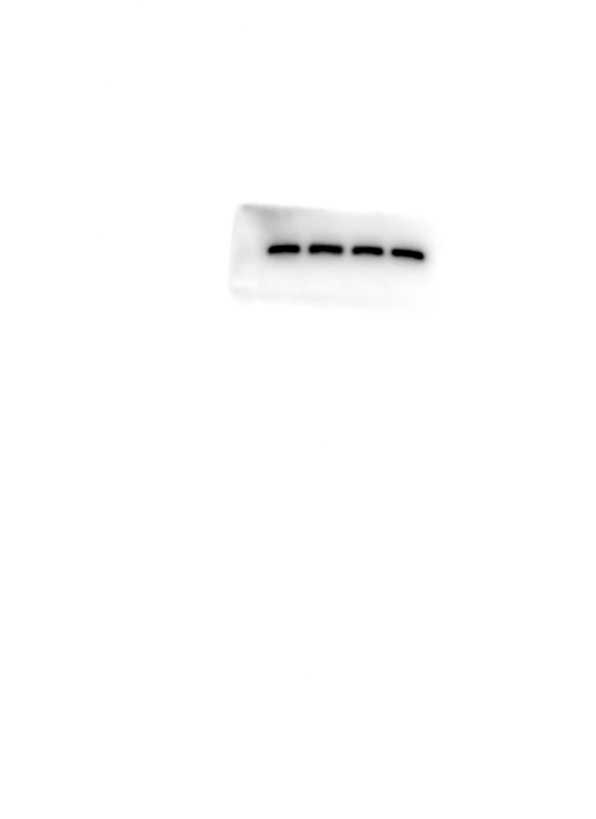

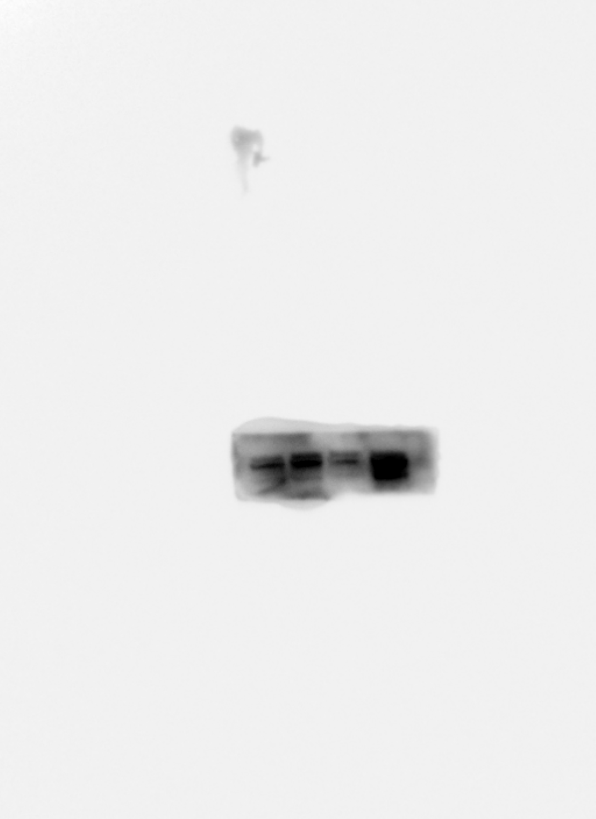

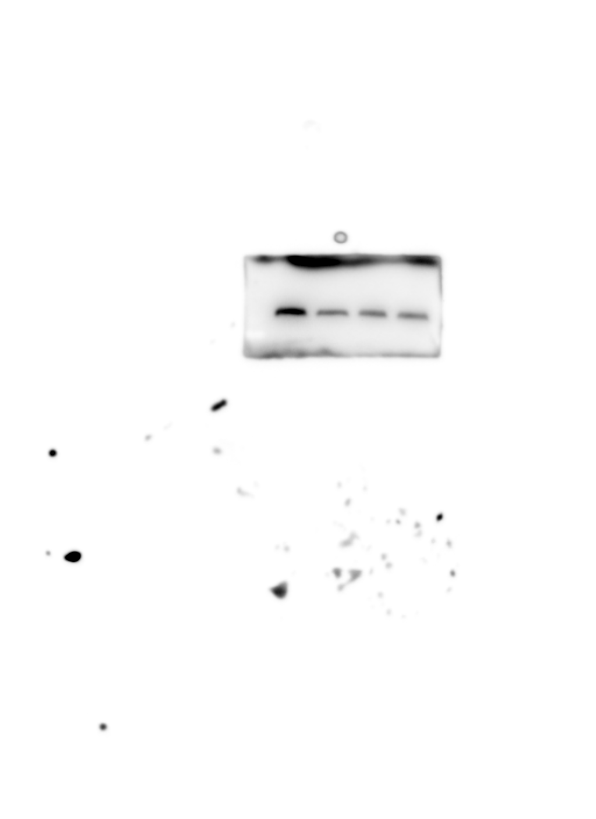

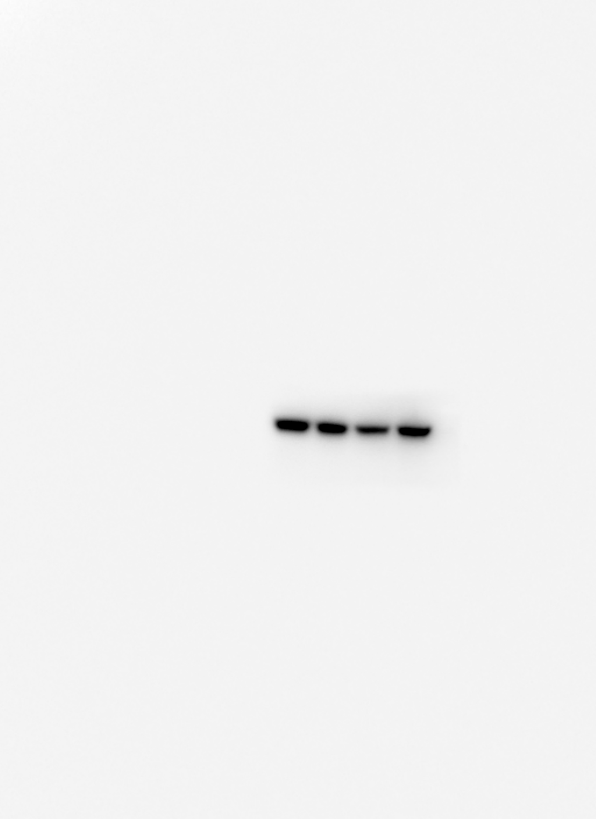

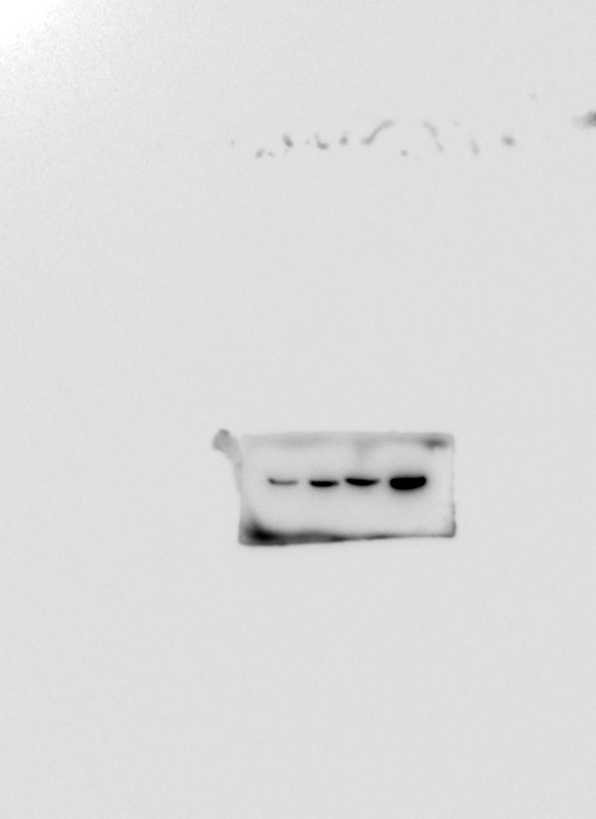

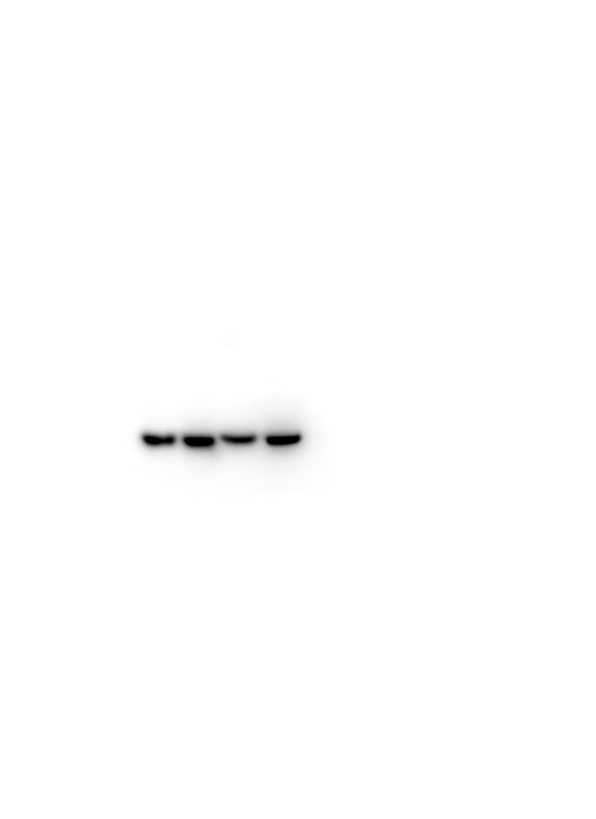

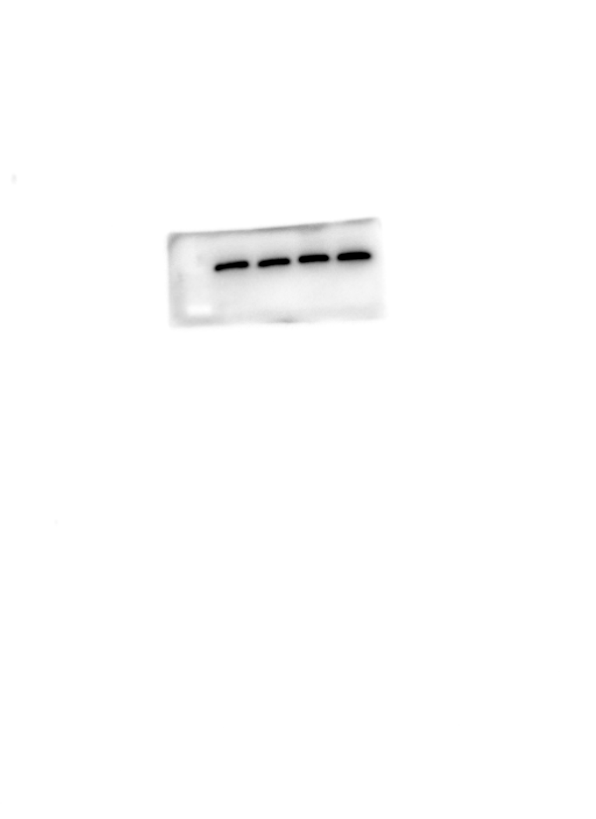

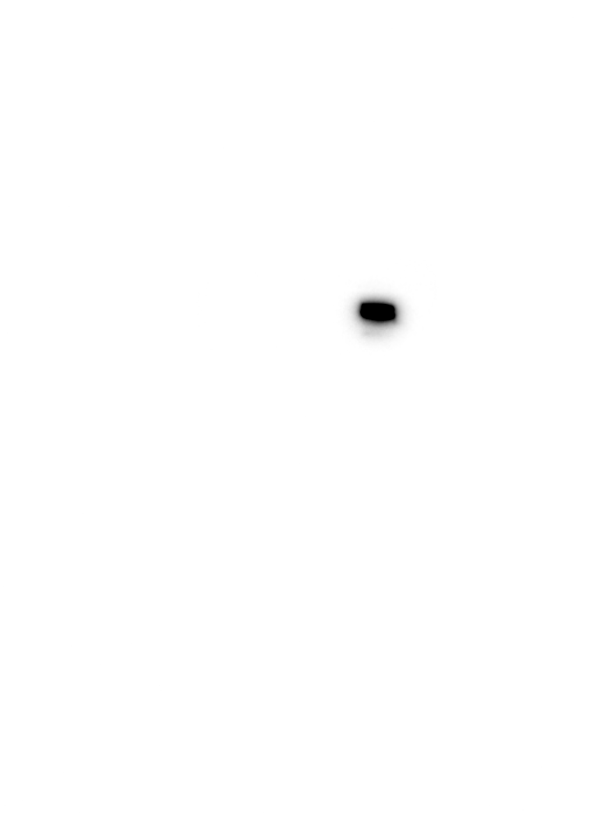

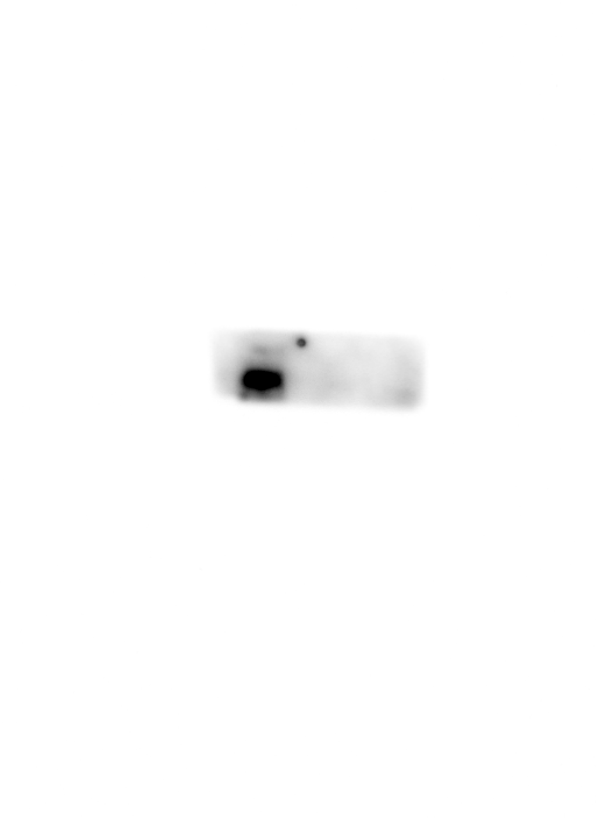

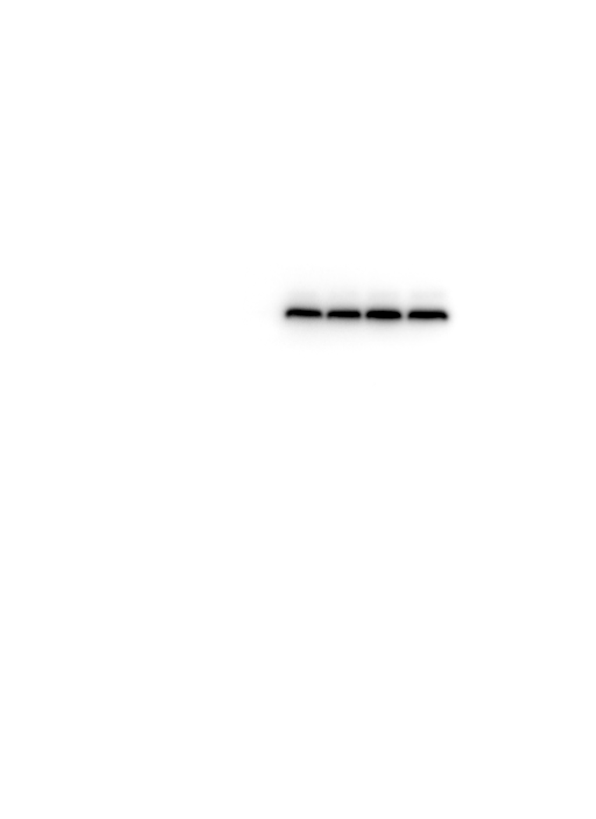

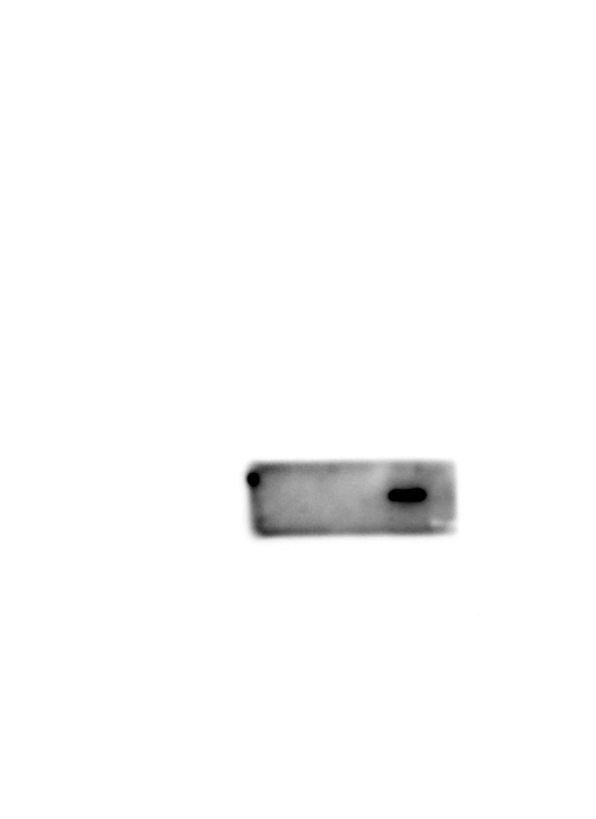

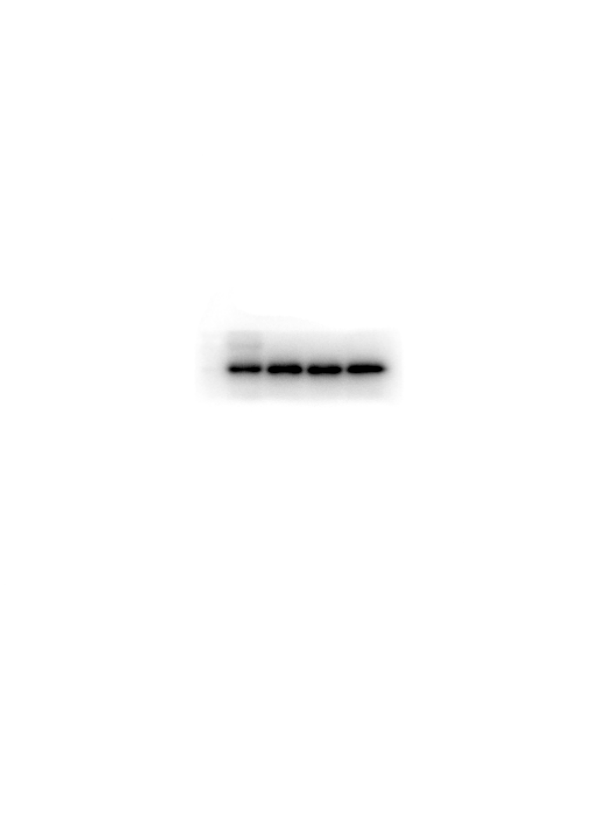

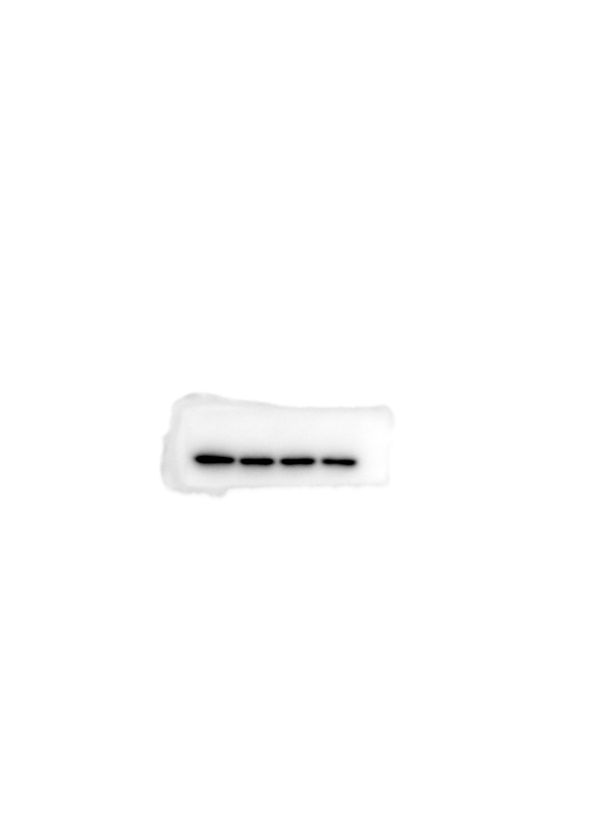

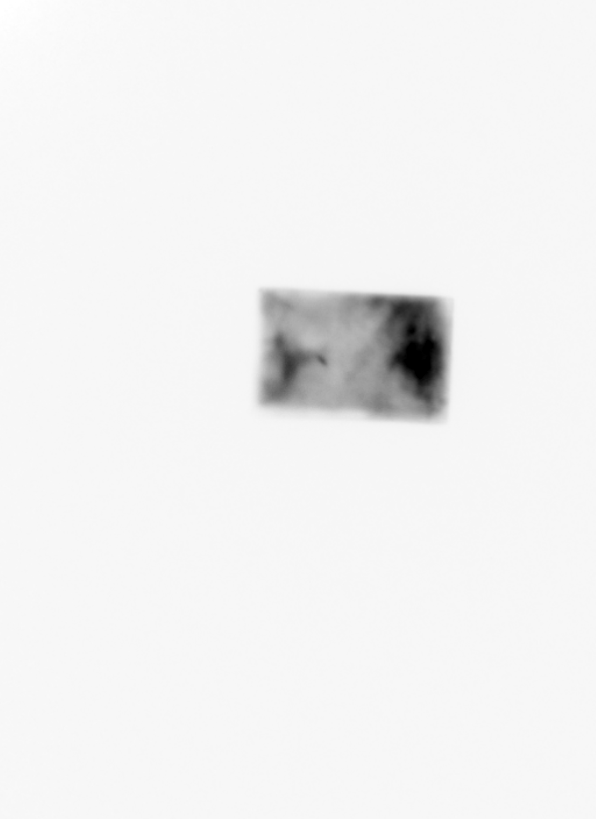

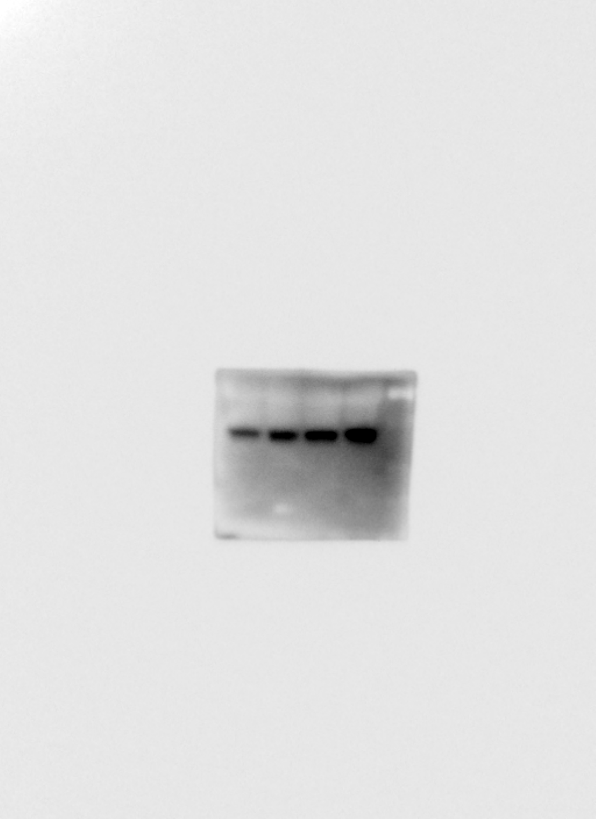

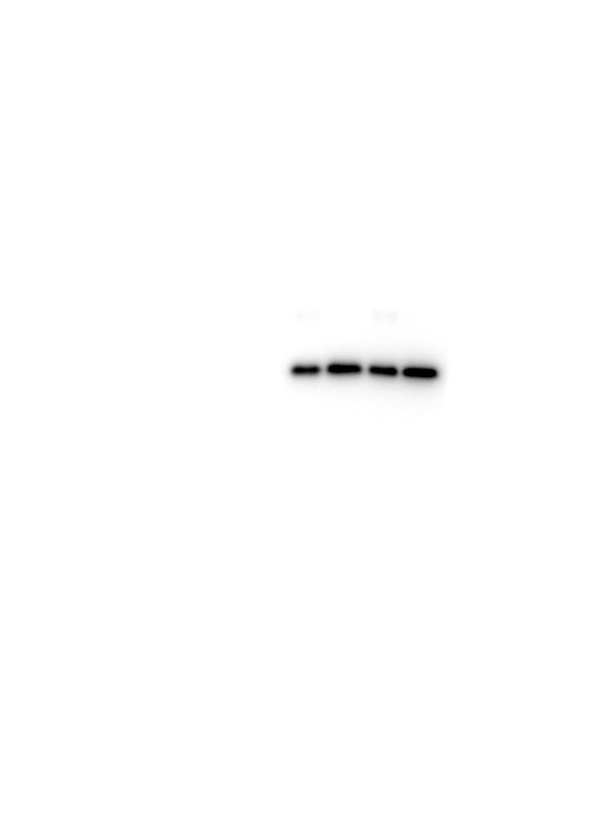

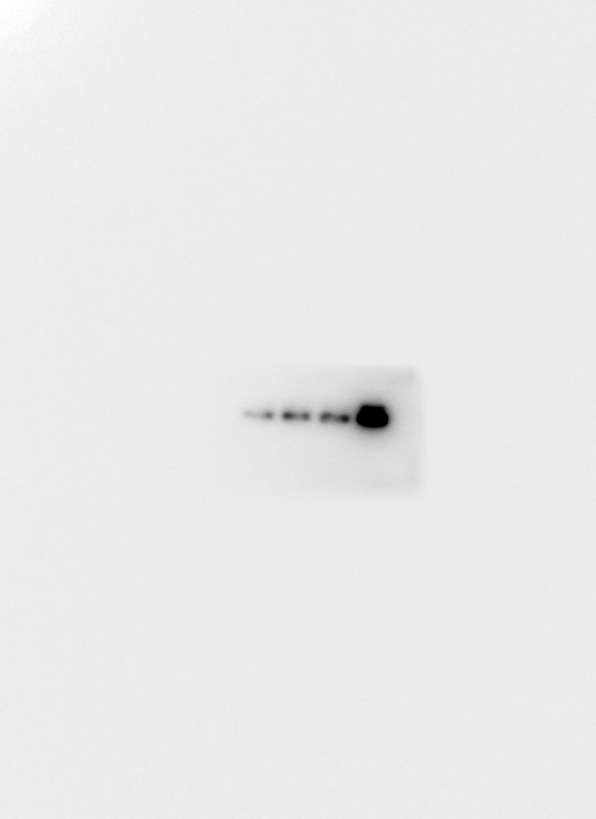

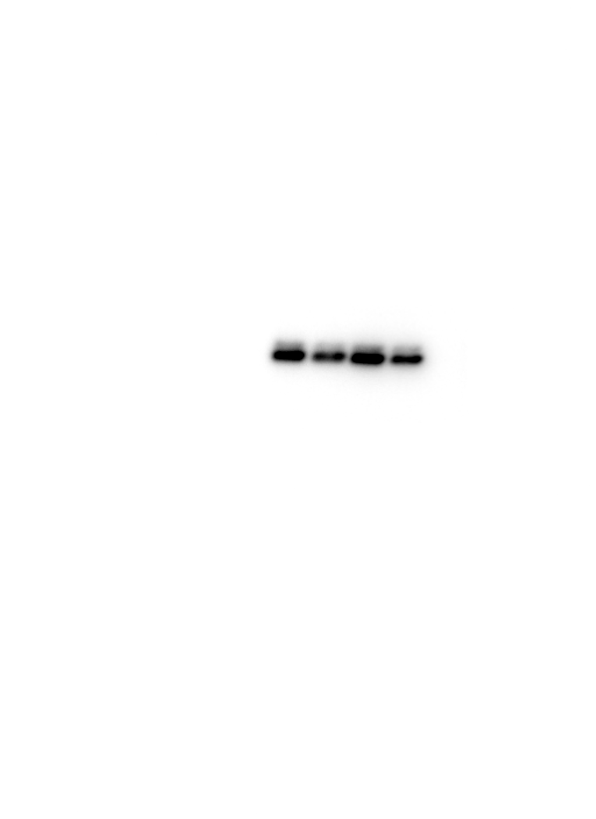

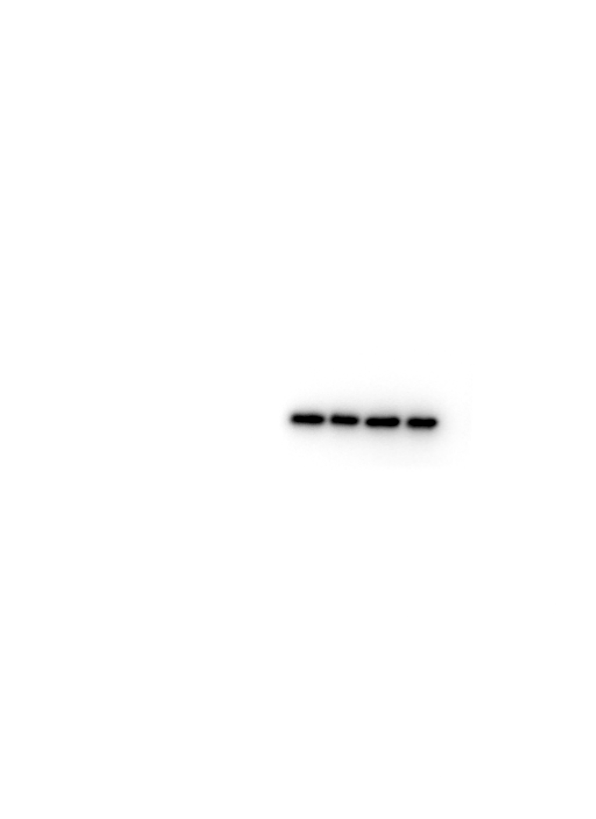

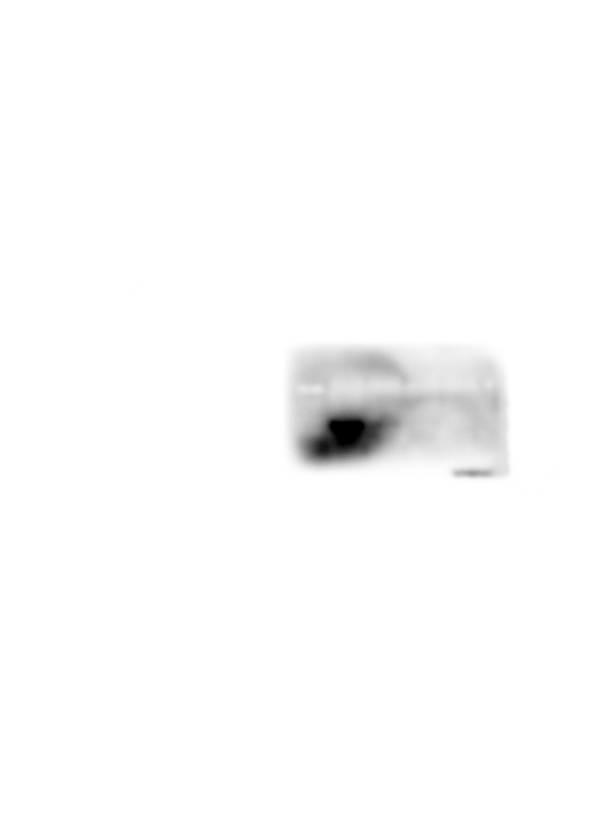

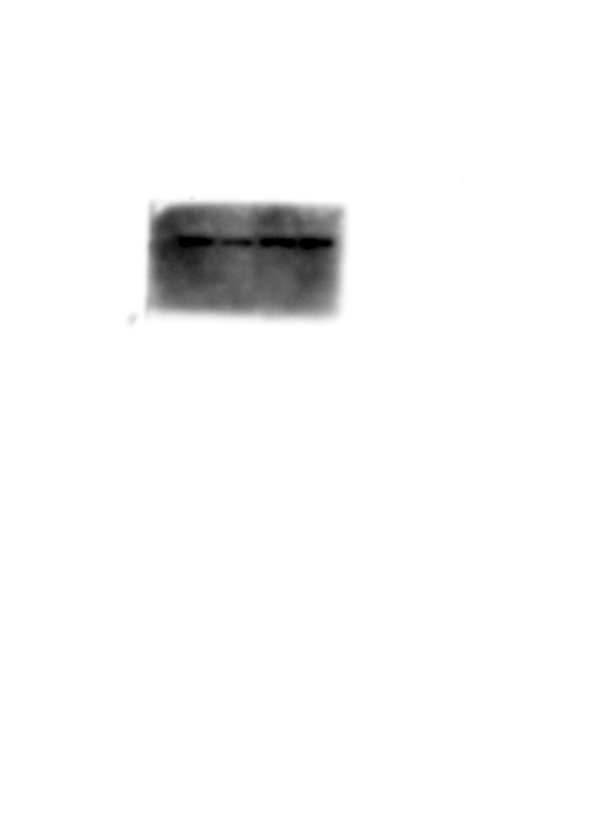

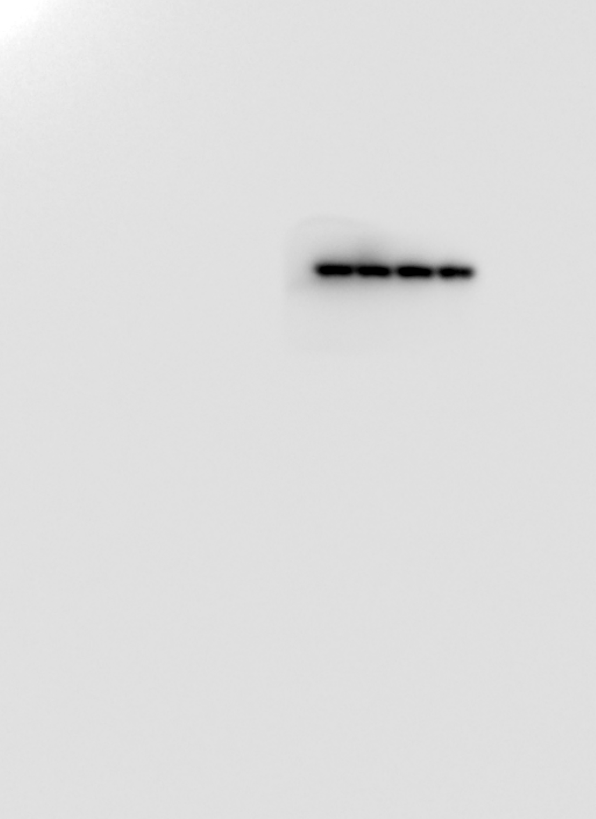

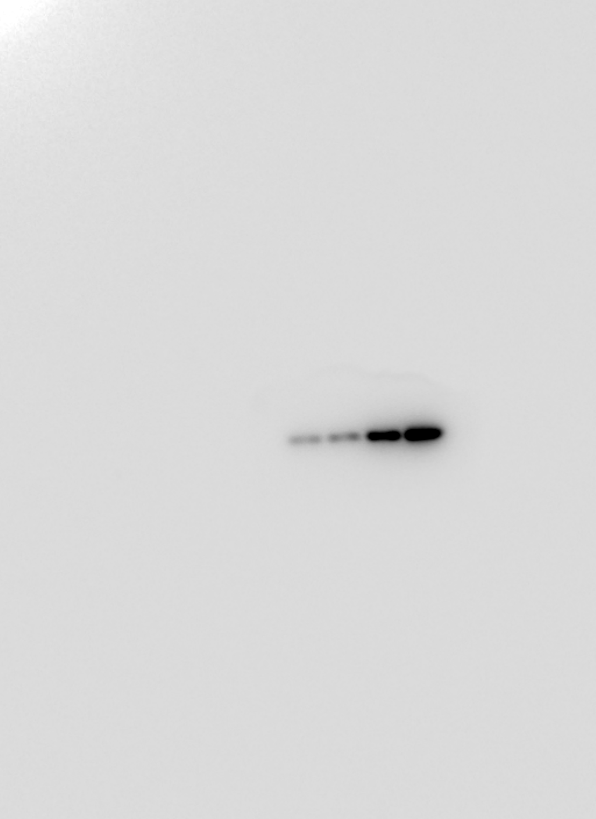

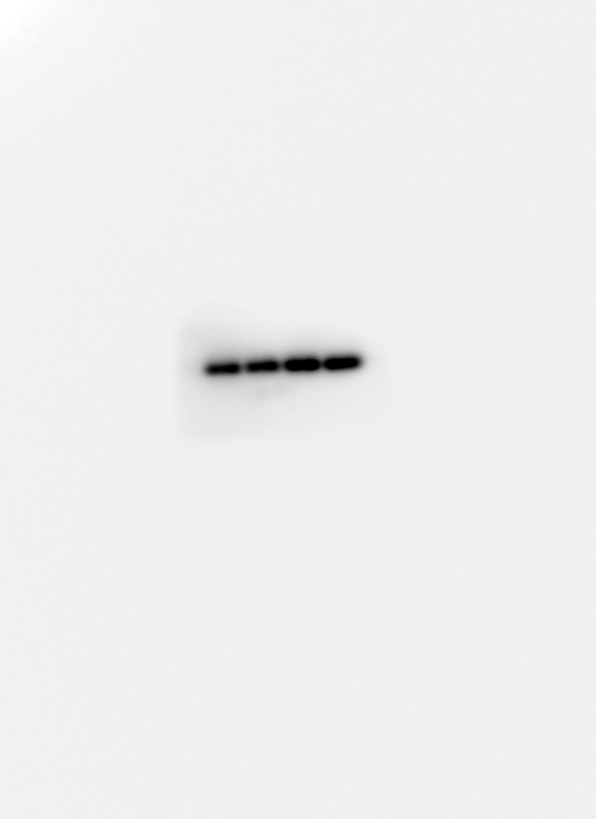

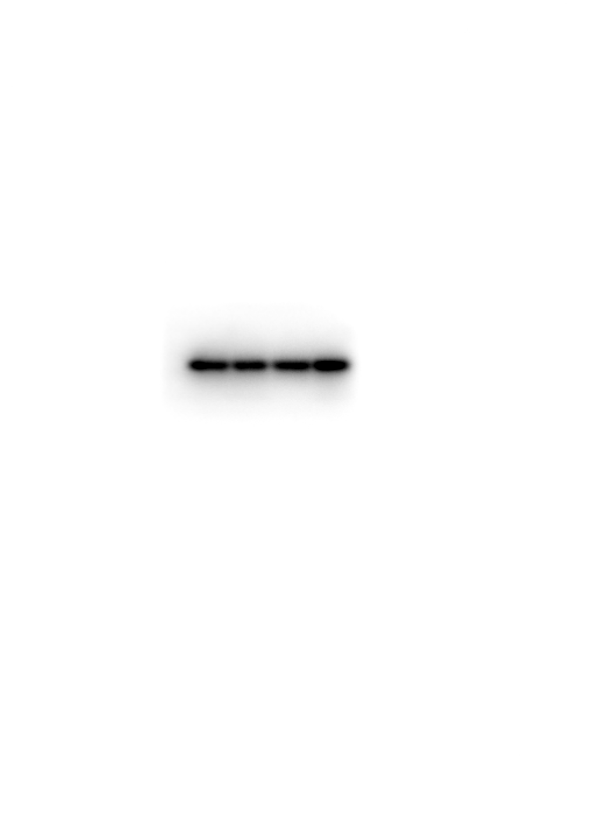

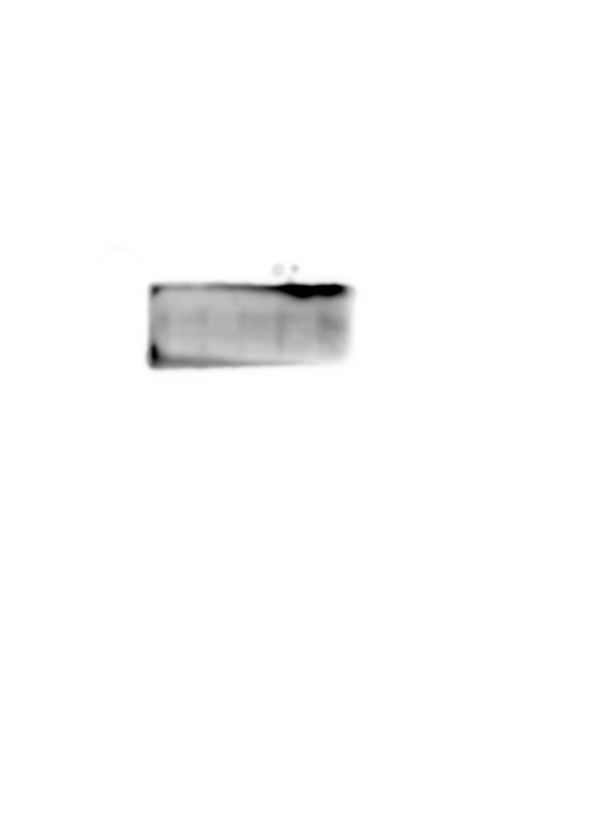

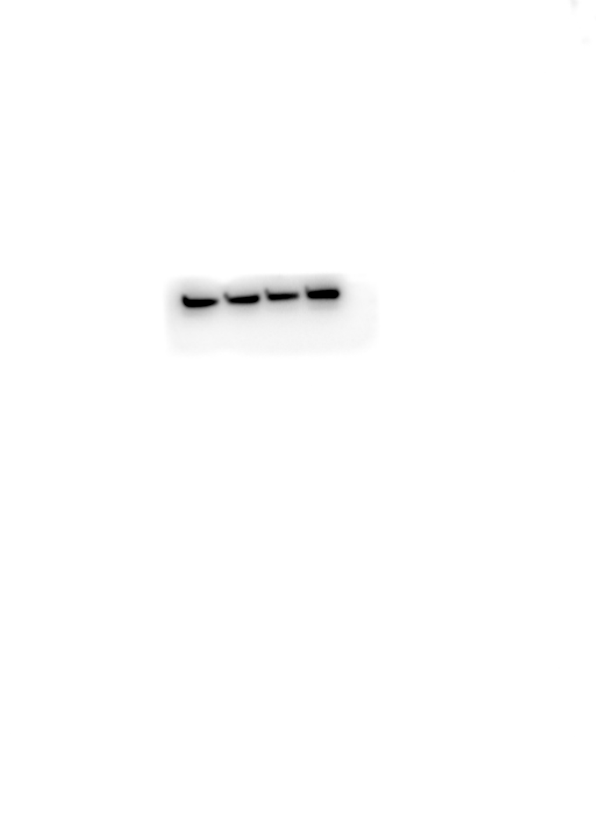

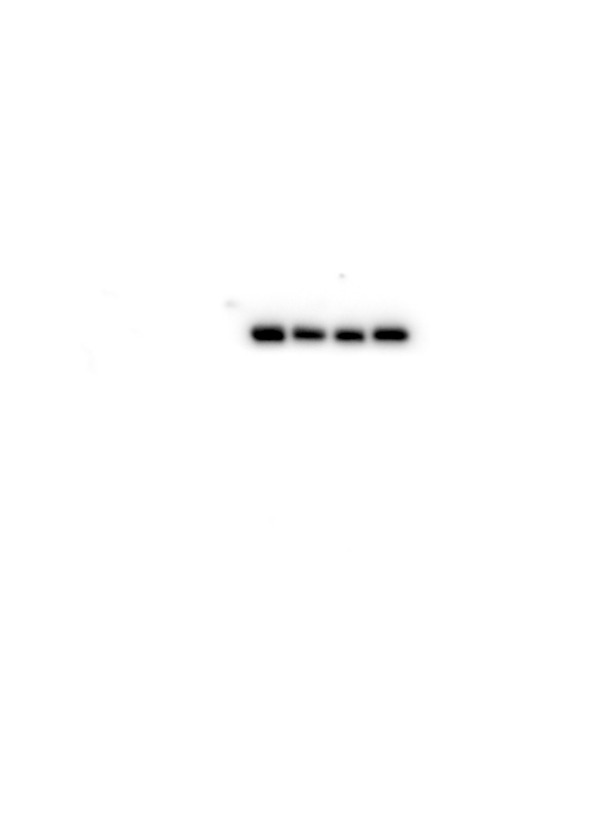

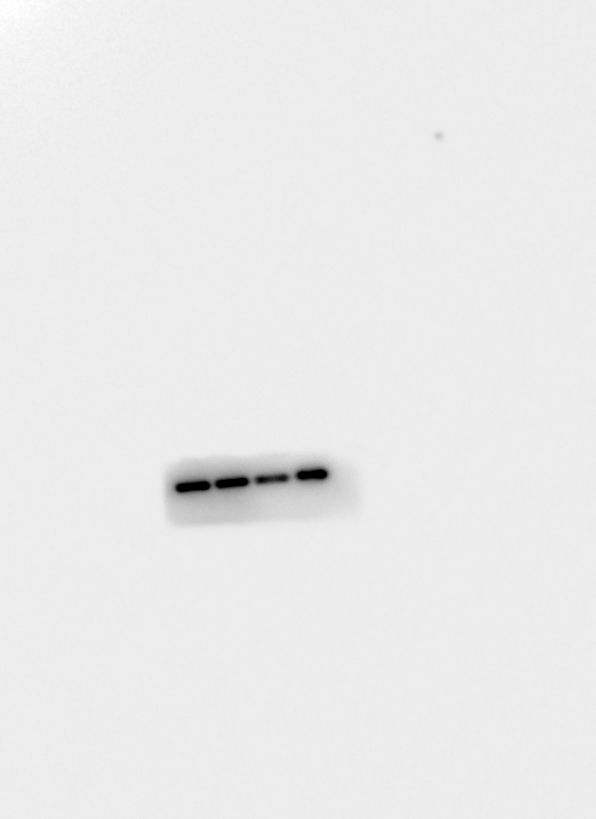

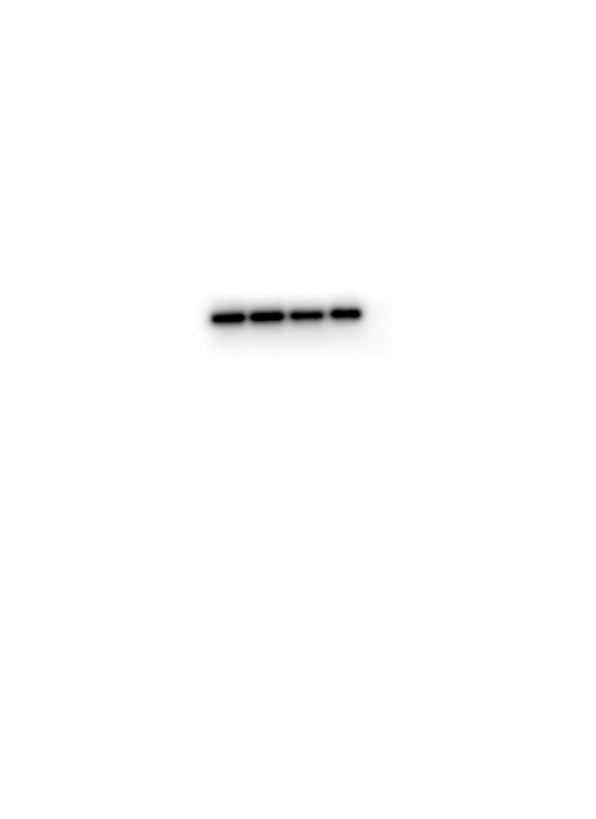

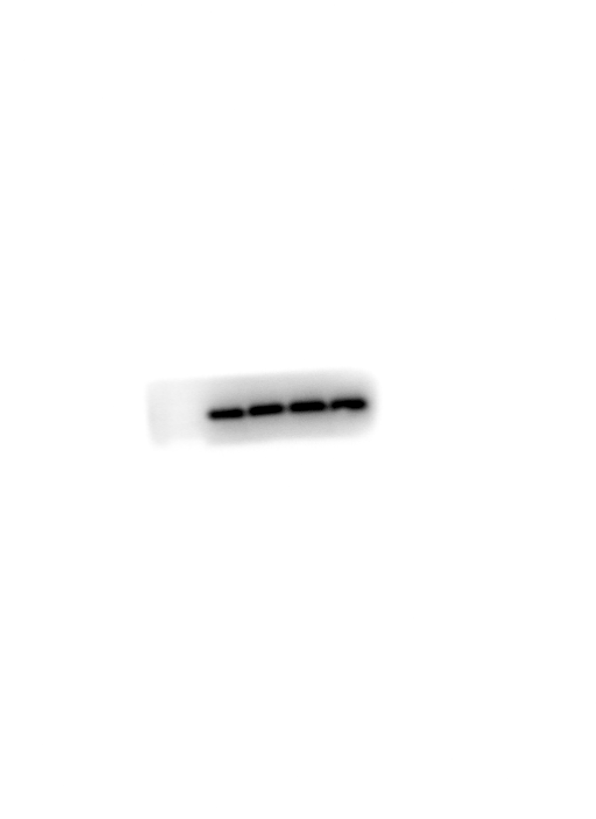

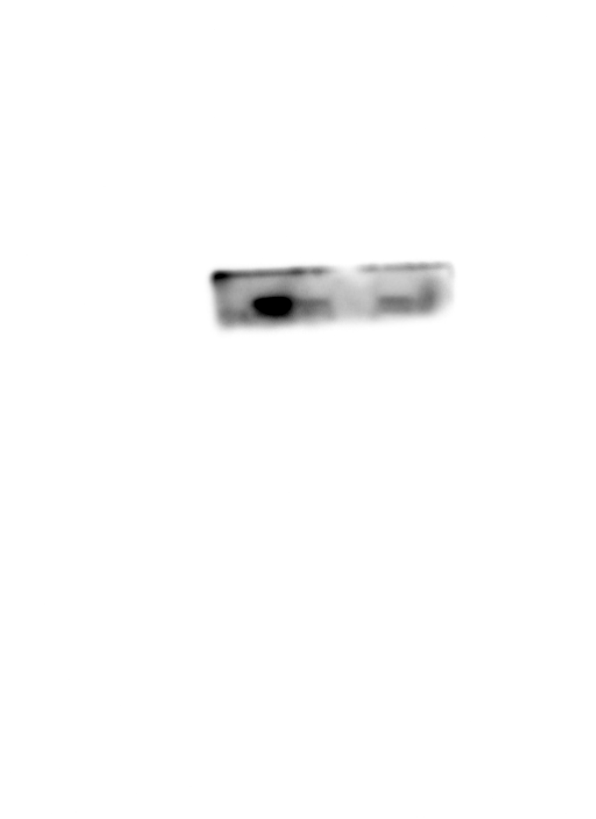

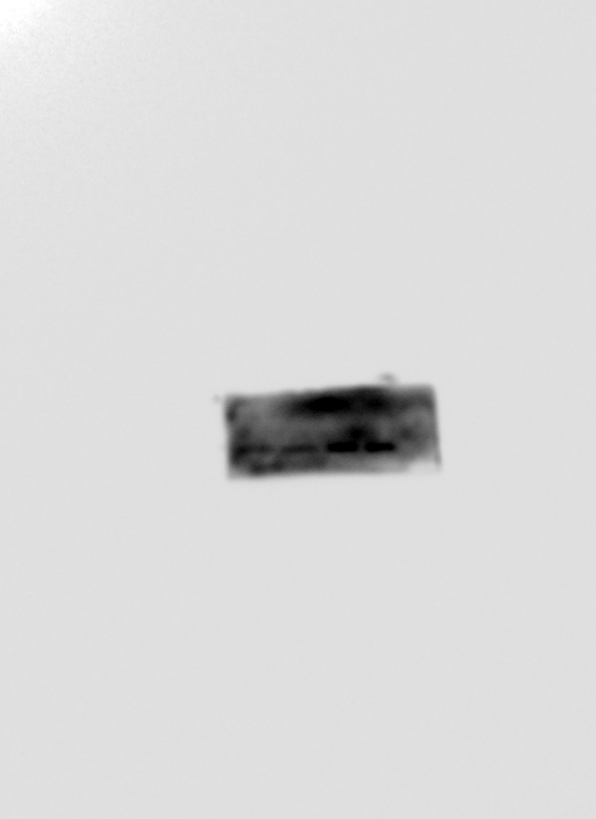

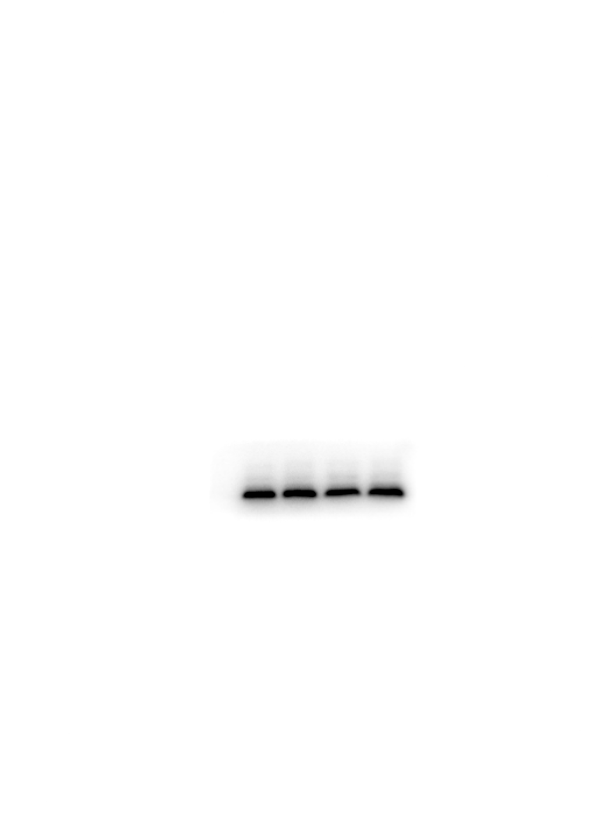

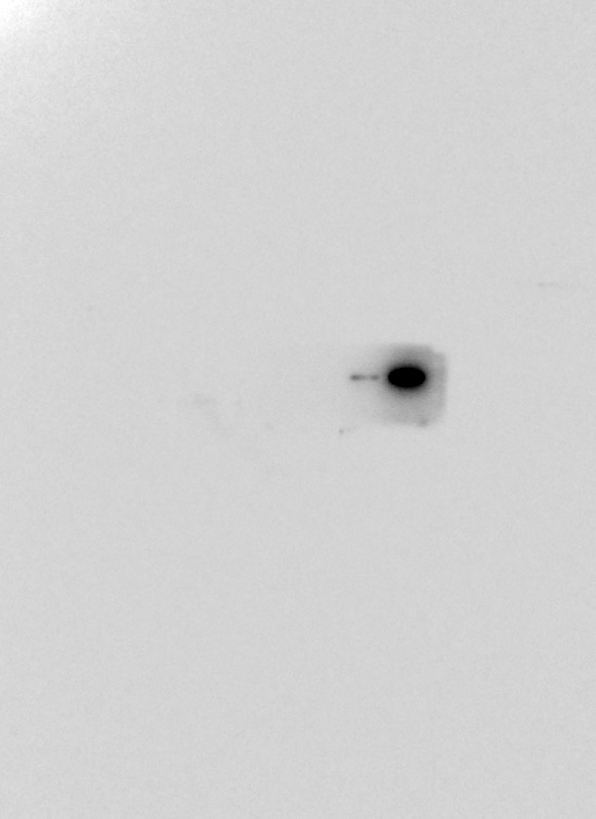

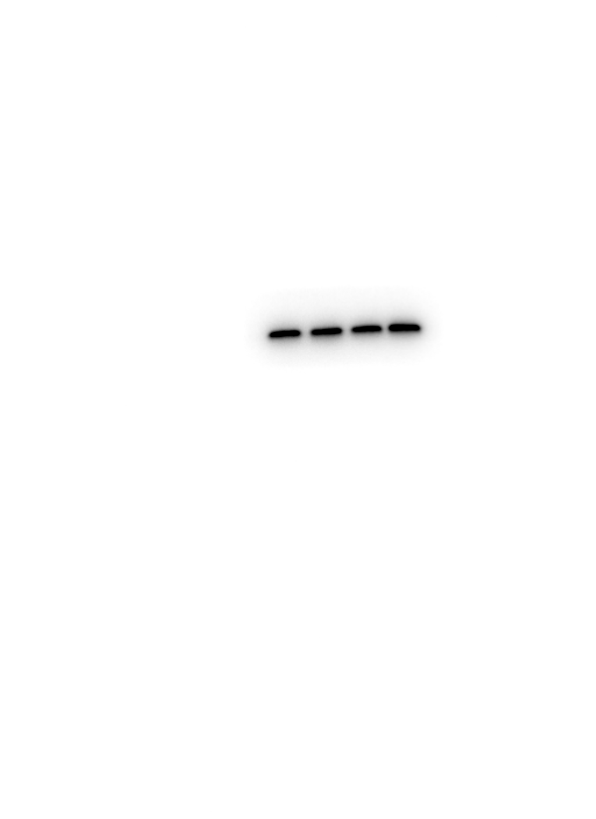

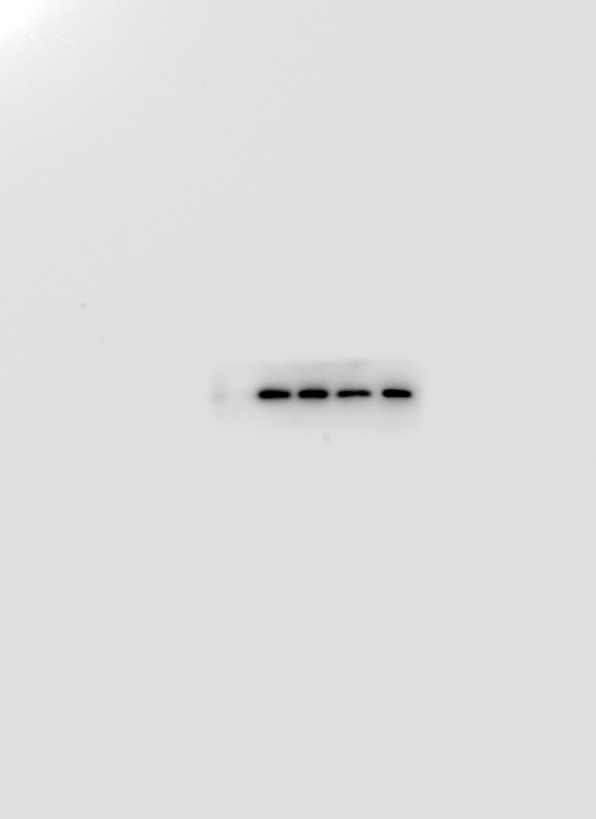


Figure 4.B


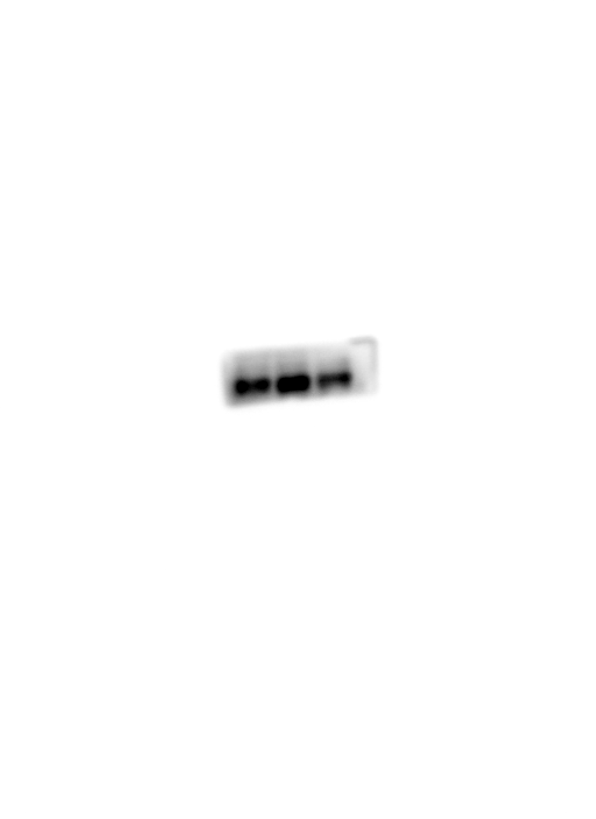

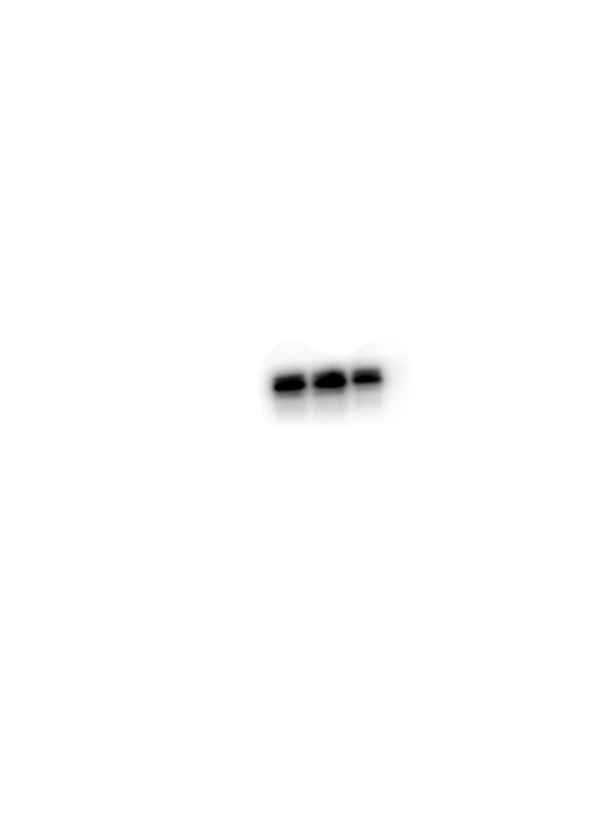

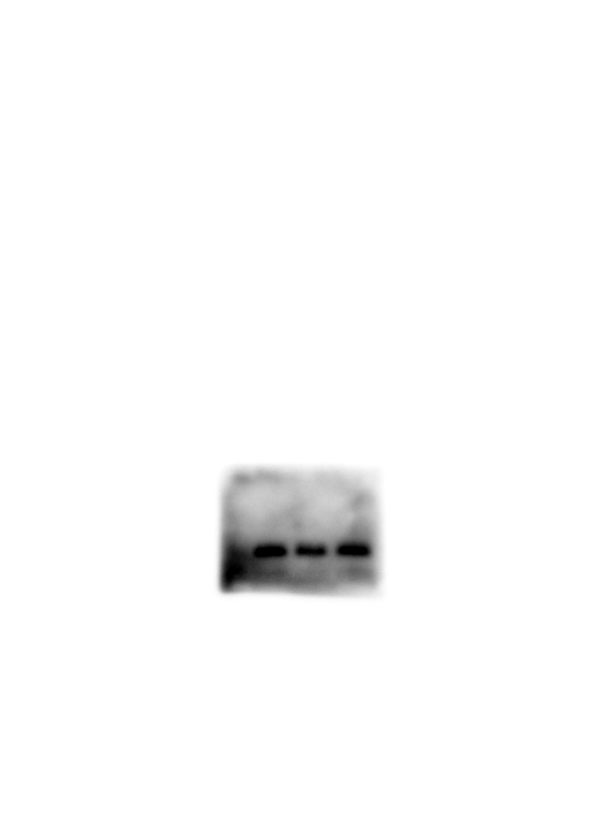

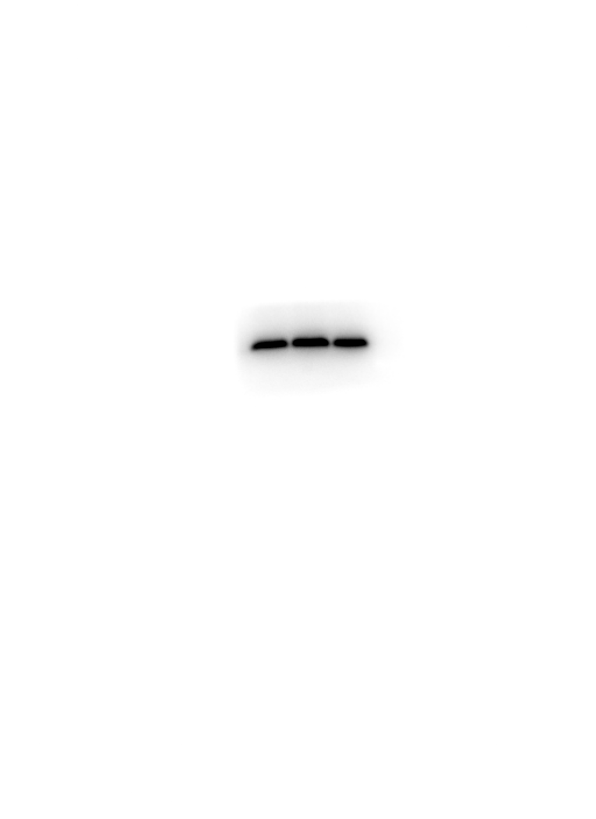

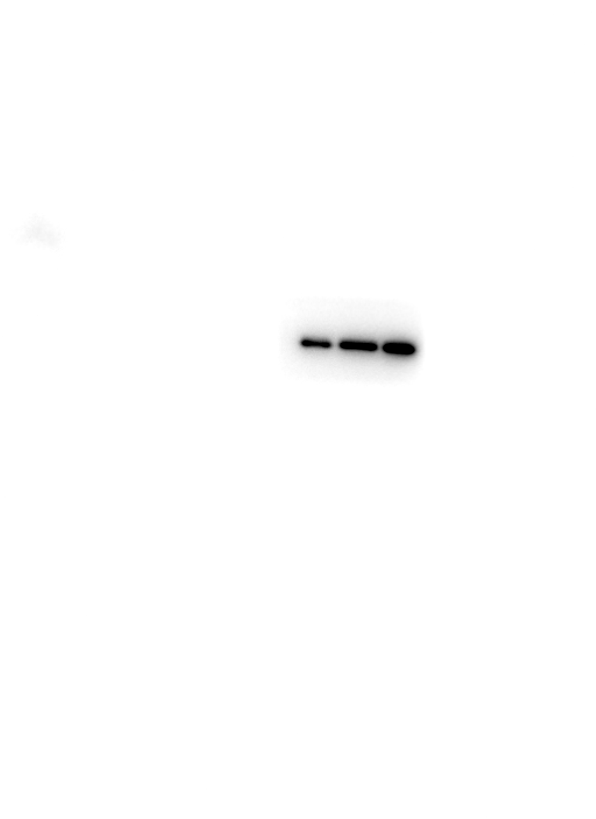

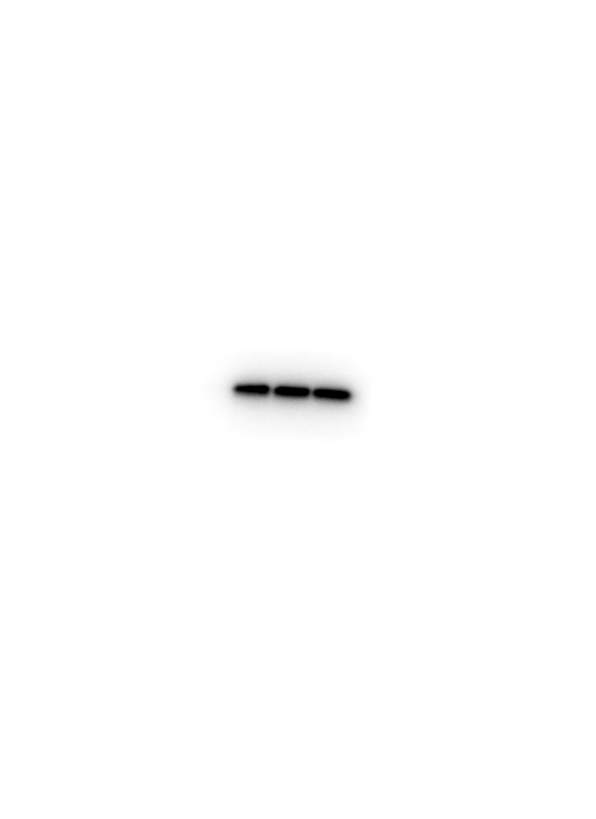

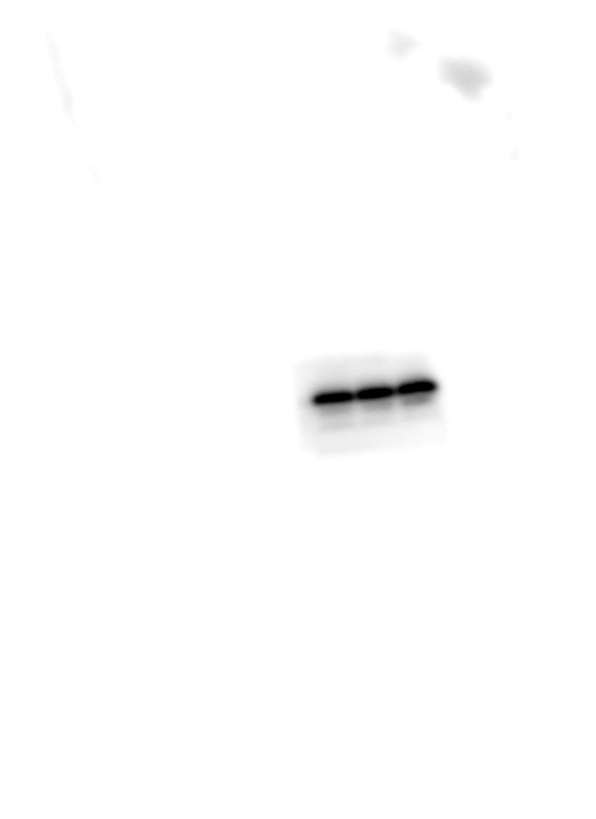

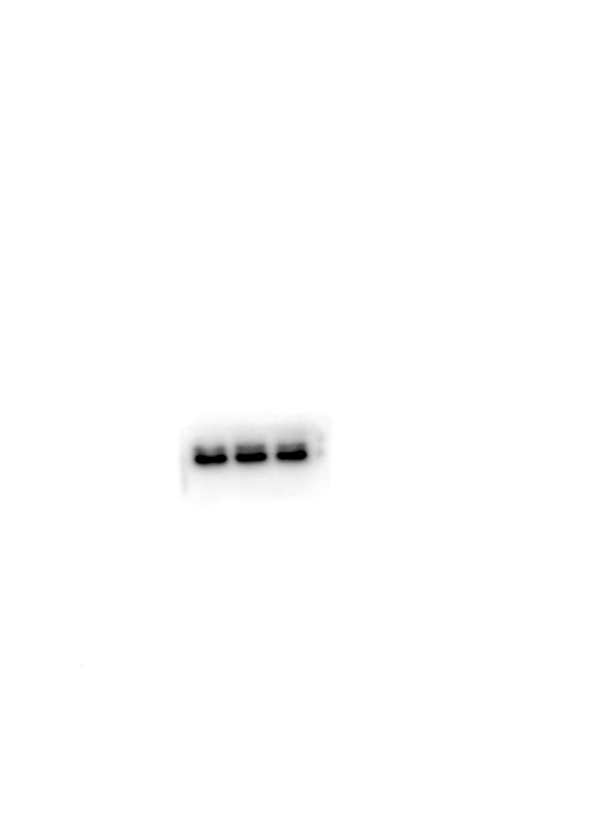

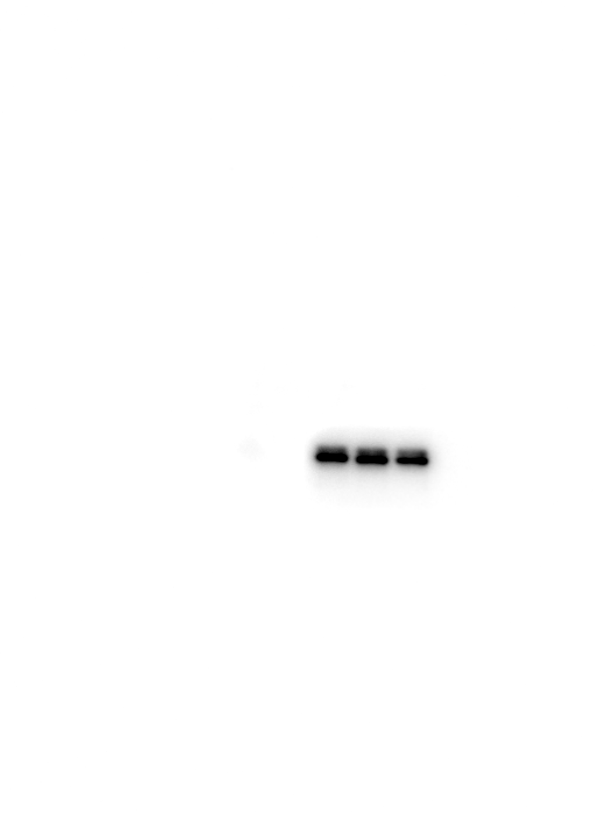

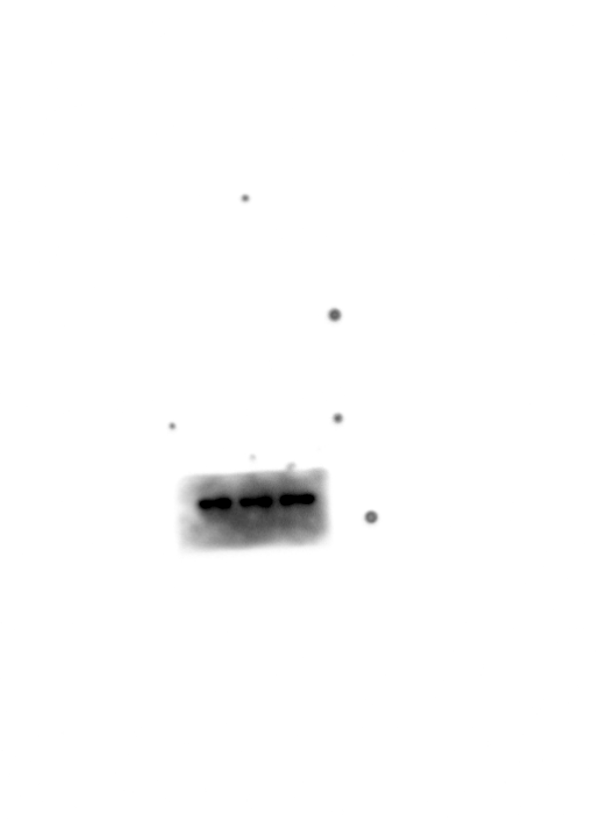

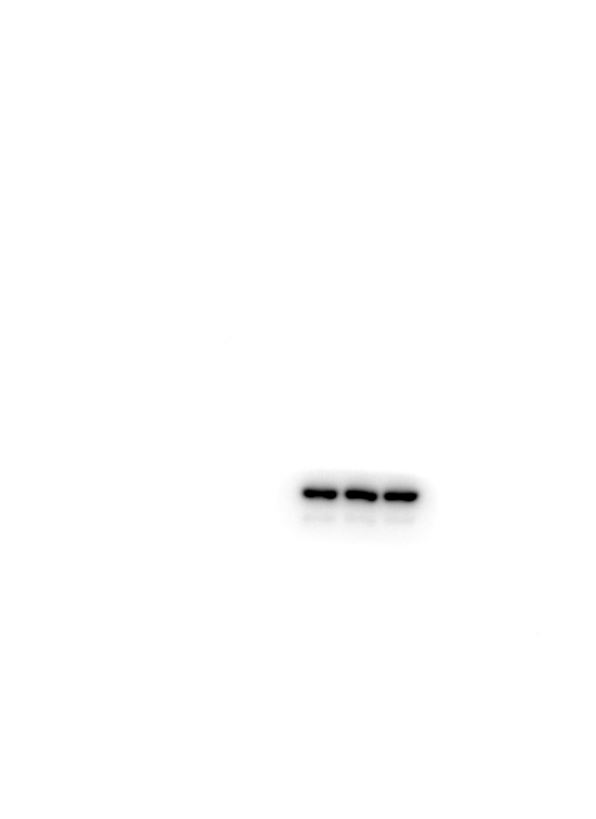

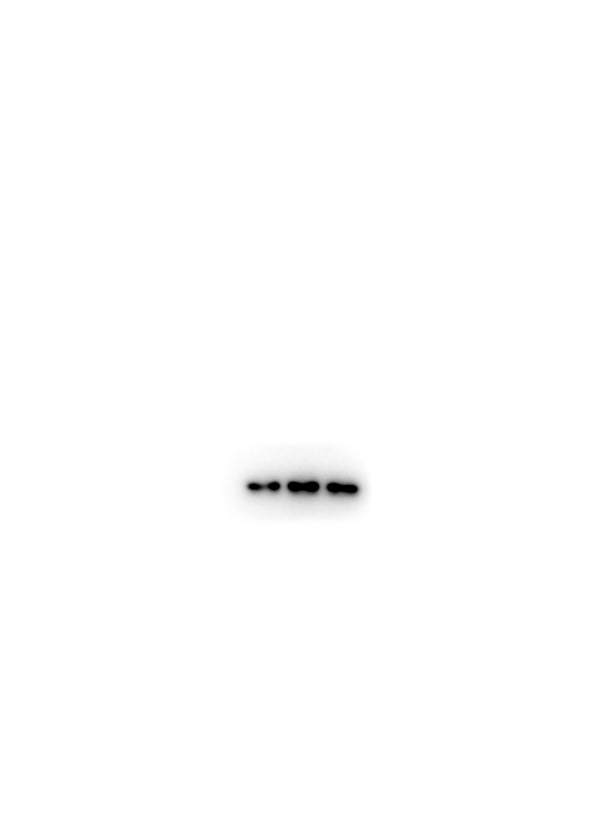

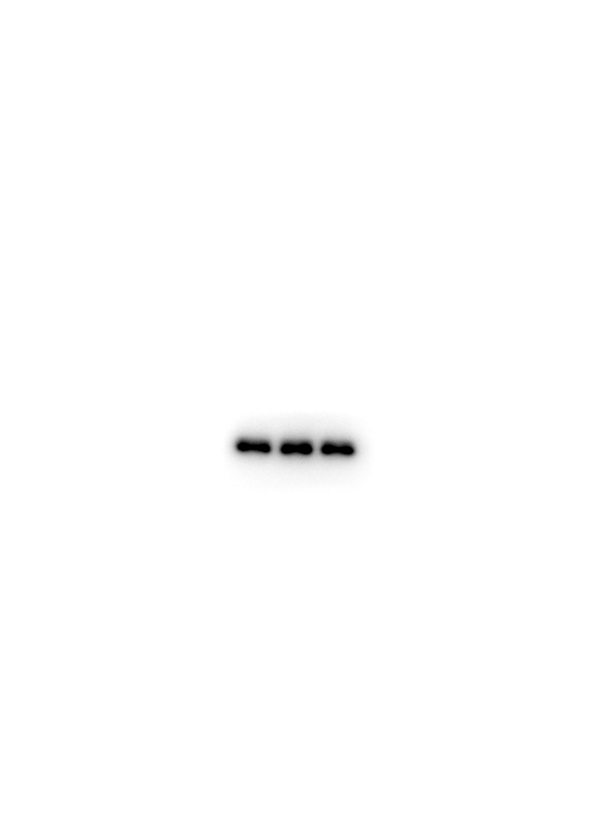

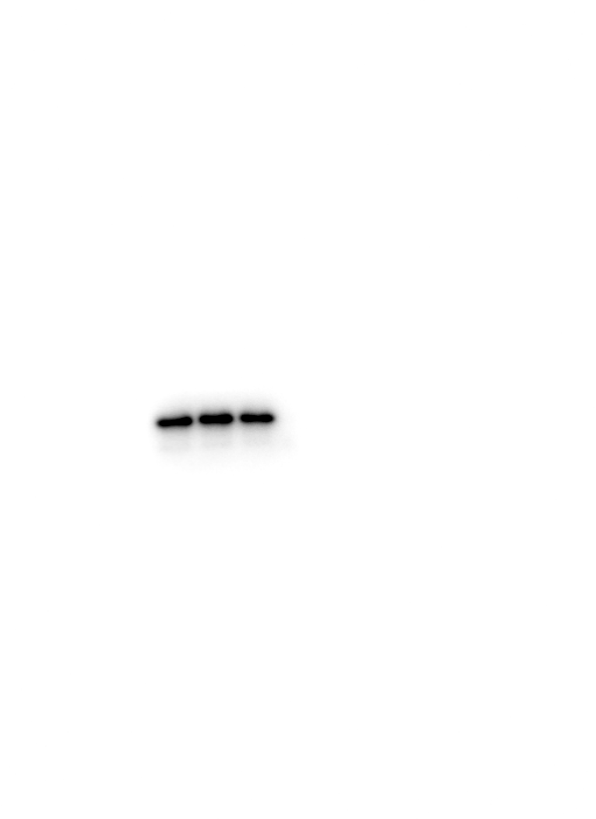

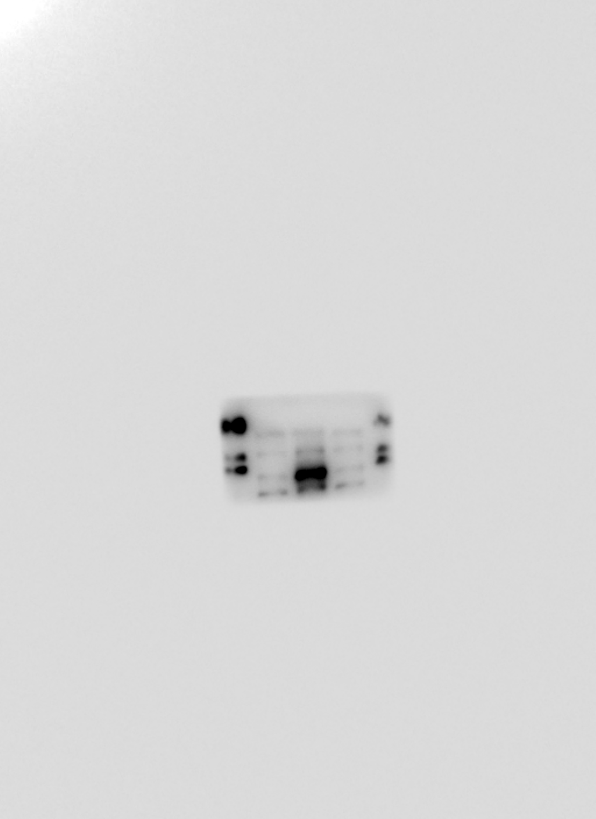


Figure 4.C

Figure 4.D

Figure 4.E, 4.F and 4.G

Figure 4.H and 4.I

Figure 4.J

Figure 4.K

Figure 4.L

Figure 5.F

Figure 6.A

ss

Figure 6.B

Figure 6.C

Figure 6.D

Figure 6.D

Figure 6.E

Figure 6.F

Figure 7.A

Figure 7.B

Figure 7.C

Figure 7.E

Figure 7.F
